# Euodiae Fructus: a review of botany, application, processing, phytochemistry, quality control, pharmacology, and toxicology

**DOI:** 10.3389/fphar.2025.1509032

**Published:** 2025-01-29

**Authors:** Yule Hao, Jiawen Qi, Xinggui Huang, Chenhao Liu, Yi Liu

**Affiliations:** School of Basic Medicine, Chengdu University of Traditional Chinese Medicine, Chengdu, China

**Keywords:** Euodiae Fructus, traditional uses, processing, phytochemistry, quality control, pharmacology, toxicology

## Abstract

Euodiae Fructus (EF) is the dried and nearly ripe fruit of *Euodia rutaecarpa*, first recorded in *Shen Nong’s Herbal Classic*. EF is a versatile Traditional Chinese Medicine (TCM) known for the effects of dispelling colds and alleviating pain, suppressing adverse qi to relieve vomiting, and boosting yang to mitigate diarrhea. However, it should be noted that EF possesses mild toxicity. In TCM prescriptions, EF is employed to treat various ailments, including abdominal pain, diarrhea, chronic non-atrophic gastritis, irritable bowel syndrome, and primary dysmenorrhea. This review collected the literature published before September 2024 on EF. An exhaustive analysis of EF literature was conducted utilizing multiple sources, namely classic TCM books and various scientific databases like Web of Science, PubMed, Elsevier, ACS, ResearchGate, Google Scholar, and Chinese National Knowledge Infrastructure. So far, more than 300 metabolites have been extracted and identified from EF, exhibiting various pharmacological effects, such as cardiovascular protection, gastrointestinal protection, neuroprotection, anti-inflammation, analgesia, anti-tumor, glucose and lipid metabolism regulation, etc. It also exhibits diverse toxicological properties and poses specific toxic risks to the liver, heart, and kidney. Nonetheless, research is scarce regarding the toxicology of EF, especially on its cardiotoxicity and nephrotoxicity. Further in-depth research is necessary to explore the mechanisms underlying EF’s pharmacological and toxicological mechanisms and to develop strategies for quality control and toxicity mitigation. The toxicity of EF can be reduced by processing, but this aspect is rarely discussed, and the quality control needs to be further standardized. Evodiamine, rutaecarpine, and limonin are the effective metabolites of EF and are also one of the causes of EF toxicity. The pharmacological effects of evodiamine and rutaecarpine have been intensely studied, but there are few studies on limonin and other metabolites of EF. Therefore, this paper focuses on the botanical characteristics, traditional applications, processing methods, phytochemistry, quality control, pharmacology, and toxicology of EF. We hope this paper provides a theoretical basis for the future high-value and high-connotation development of EF.

## 1 Introduction

EF is a dry, nearly ripe fruit of the genus *Euodia rutaecarpa*, first recorded in the top grade of *Shen Nong’s Herbal Classic* (Dong Han Dynasty, A.D. 25–220). It relieves cold and pain, suppresses adverse qi to relieve vomiting, and enhances yang to stop diarrhea. Nonetheless, it is essential to acknowledge that EF has a slight toxic effect. EF ranks among the most prevalent botanical drugs clinically in TCM, boasts a history of more than 2000 years, and has been formally listed in various editions of Chinese Pharmacopoeia (ChP) (https://www.nmpa.gov.cn/). Lately, numerous studies have concentrated on examining the metabolites, pharmacological effects, clinical function, and toxicology of EF. Up to this point, more than 300 metabolites have been extracted and pinpointed from EF (Xiao et al., 2023). Contemporary research indicates that EF’s primary active elements comprise alkaloids, terpenoids, flavonoids, volatile oils, and other compounds (Liu L. et al., 2020). Among them, evodiamine, rutaecarpine, and limonin are characteristic metabolites (Tian et al., 2019). Research in pharmacology reveals that EF, along with its raw extract and refined form, offers a range of pharmacological effects, such as cardiovascular protection, gastrointestinal protection, neuroprotection, anti-inflammation, analgesia, anti-tumor, and glucose and lipid metabolism regulation. In clinical settings, this medication serves as both a supplement and a substitute treatment for conditions like abdominal pain, vomiting, diarrhea, indigestion, hypertension, eczema, and oral ulcers (Huang et al., 2021). EF, combined with various botanical drugs, is effective in gastrointestinal diseases, headaches, vomiting, skin diseases, dysentery, menorrhagia, and postpartum hemorrhage (Li D. et al., 2022).

However, it is important to recognize that excessive use of EF may lead to stomach pain, vomiting, blurred vision, and other toxic symptoms (Cai et al., 2006; Ma et al., 2018). As EF’s clinical application has expanded, its toxicity has become increasingly apparent. It is widely accepted among scholars that the toxicity of EF may be attributed to reactive metabolites (RMs) produced by the metabolic activation of evodiamine, rutaecarpine, and limonin (He et al., 2024). Studies have demonstrated that different parts of EF can induce varying degrees of hepatic injury in rats (Liu et al., 2015), and EF has obvious toxic damage to the human liver (Teschke et al., 2014). Some studies have also highlighted the heart and kidney as possible focal points for EF toxicity. Yet, investigations into EF cardiotoxicity and nephrotoxicity processes are scarce and insufficient for the specific toxic risks and potential disadvantages of EF. Exploring the toxic metabolites of EF and methods for mitigating its toxicity is crucial to guiding safe clinical use. This paper reviews the plant morphology, traditional application, processing, phytochemistry, quality control, pharmacology, toxicology, monitoring, and prevention of EF. Particular emphasis is placed on discussing the mechanisms of EF-induced cardiotoxicity and nephrotoxicity, strategies for reducing and controlling EF’s toxicity, and preventive measures for clinical monitoring.

## 2 Botany

**FIGURE 1:**
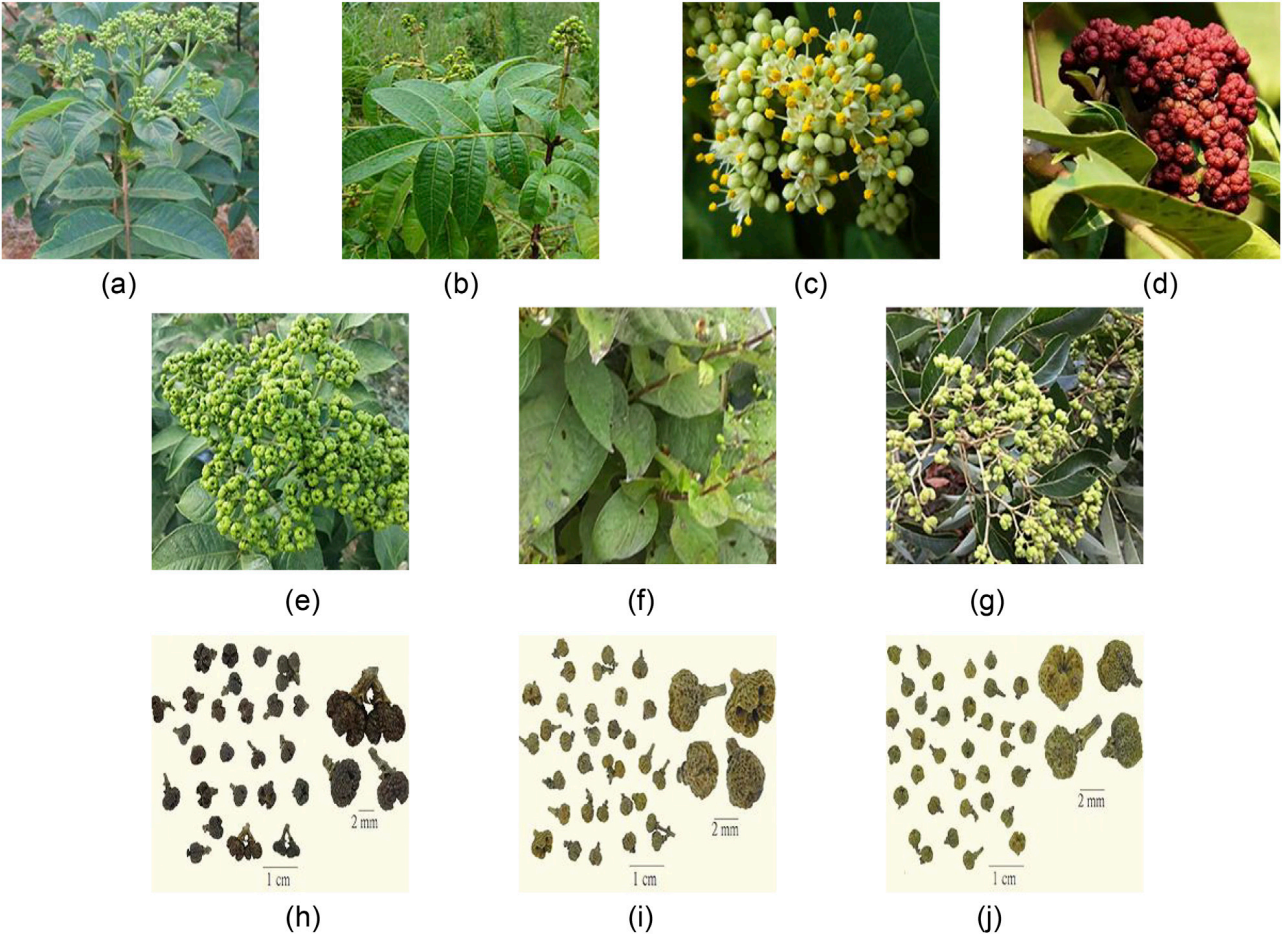
The above-ground portion **(A)**, leaves **(B)**, flowers **(C)**, fruits **(D)**, ER **(E)**, ERO **(F)**, ERB **(G)**, LEF **(H)**, MEF **(I)** and SEF **(J)** of *Euodia rutaecarpa*.

The ChP recorded the dried and nearly ripe fruit of three plants of the genus *Euodia rutaecarpa* (Juss.) Benth. (ER), *Euodia rutaecarpa* (Juss.) Benth. var. *officinalis* (Dode) Huang (ERO), *Euodia rutaecarpa* (Juss.) Benth. var. *bodinieri* (Dode) Huang (ERB). *Euodia rutaecarpa* is also divided into large grains and small grains. ER primarily supplies large grains, categorized into large EF flowers (LEF) and medium EF flowers (MEF). LEF has reached full ripeness, the fruit shows cracks, and its effectiveness is subpar. Approximately seven mature MEFs, characterized by a yellowish-green and potent odor, are commonly utilized in medical treatments. ERO and ERB primarily supply diminutive grains, predominantly consisting of small EF flowers (SEF), often immature and green. The diminutive size of ERO and ERB fruits typically classifies them as ER varieties, distinct from ER due to their unique, strong smells. The botanical characteristics of ER, ERO, and ERB are similar to those of dried fruits, as shown in Figure 1.

The *Euodia rutaecarpa* are shrubs or trees, standing 3–5 m high, and are thickly adorned with grayish yellow, rust-red downy hair or have few hairs and dark purplish-red shoots. The leaves have 5–11 leaflets, ovate, elliptic, or lanceolate, 6–18 cm long and 3–7 cm wide. Inflorescences are terminal and dioecious. Male inflorescence flowers are separated from each other, with petals measuring 3–4 mm long. Female inflorescences are dense or distant, and petals measure 4–5 mm long. Most of the sepals and petals are 5 pieces, occasionally 4 pieces, arranged in a pincer pattern. The fruit is spherical or slightly pentagonal oblate, and the surface is dark greenish, yellow, or brown. The outer pericarp has oil spots. The inner pericarp is a thin shell or woody, waxy yellow or brown, and the ovary can be seen as 5-located with 1 seed per mericarp. At its peak is a star-shaped fissure with five points, while its base features a calyx and a fruit stalk and is adorned with yellow hairs. The quality is hard and crisp, with a full green color and rich aroma is better. However, the botanical characteristics of ER, ERO, and ERB are different in growth form, maturity period, ecological environment, and resource distribution, as shown in Table 1.

**TABLE 1:** The differences in botanical descriptions between three plants.

Plant	Botanical morphology	Mature period	Ecological environment	Resource distribution
ER	Odd-pinnate compound leaves opposite, leaflets thin to thick papery, ovate or elliptic, apex abruptly narrowed into mucronate, base cuneate to broadly cuneate or rounded, entire margin or indistinctly serrate, lateral veins indistinct, both sides are yellowish brown puberulent, especially on veins, with distinct large and numerous oil spots. The fruit is single or several together, spherical or slightly pentagonal and oblate, about 3–6 mm in diameter	The flowering period is from June to August, and the fruiting period is from September to October	It grows at low elevations under or on the margins of open forests facing the sun	It is mainly distributed in Shaanxi, Gansu, Anhui, Zhejiang, Fujian, Taiwan, Hubei, Hunan, Guangdong, Guangxi, Sichuan, Guizhou, and Yunnan
ERO	The leaves are narrow, oblong to narrowly lanceolate, apex acuminate or long acuminate, and the leaflets are distant. Both sides are densely villous, the veins are the densest, and the oil glands are thick. The inflorescence rachis is often covered with yellowish villous hairs. The ripe inflorescence is not as dense as the orthodox. The fruit is smaller and less than 3.5 mm in diameter. The seeds are bluish-black	The flowering period is from July to August, and the fruiting period is from September to October	Born in the hillside grass	It is mainly distributed in Zhejiang, Jiangxi, Hubei, Hunan, Guangxi, Sichuan, and Guizhou
ERB	Branchlets are sparsely hirsute with yellow rust or silky color, leaf rachis villous. The leaf shape is oblong, lanceolate, ovate-lanceolate; the upper surface midvein is slightly sparsely pubescent, the lower vein is pubescent, the lateral vein is precise, and the oil gland is small. The fruit is small, mung bean-colored in appearance, and less than 3.5 mm in diameter	The flowering period is from July to August, and the fruiting period is from September to October	Born on the village side of the road and hillside grass	It is mainly distributed in Jiangxi, Hunan, Guangdong, Guangxi, and Guizhou

**FIGURE 2:**
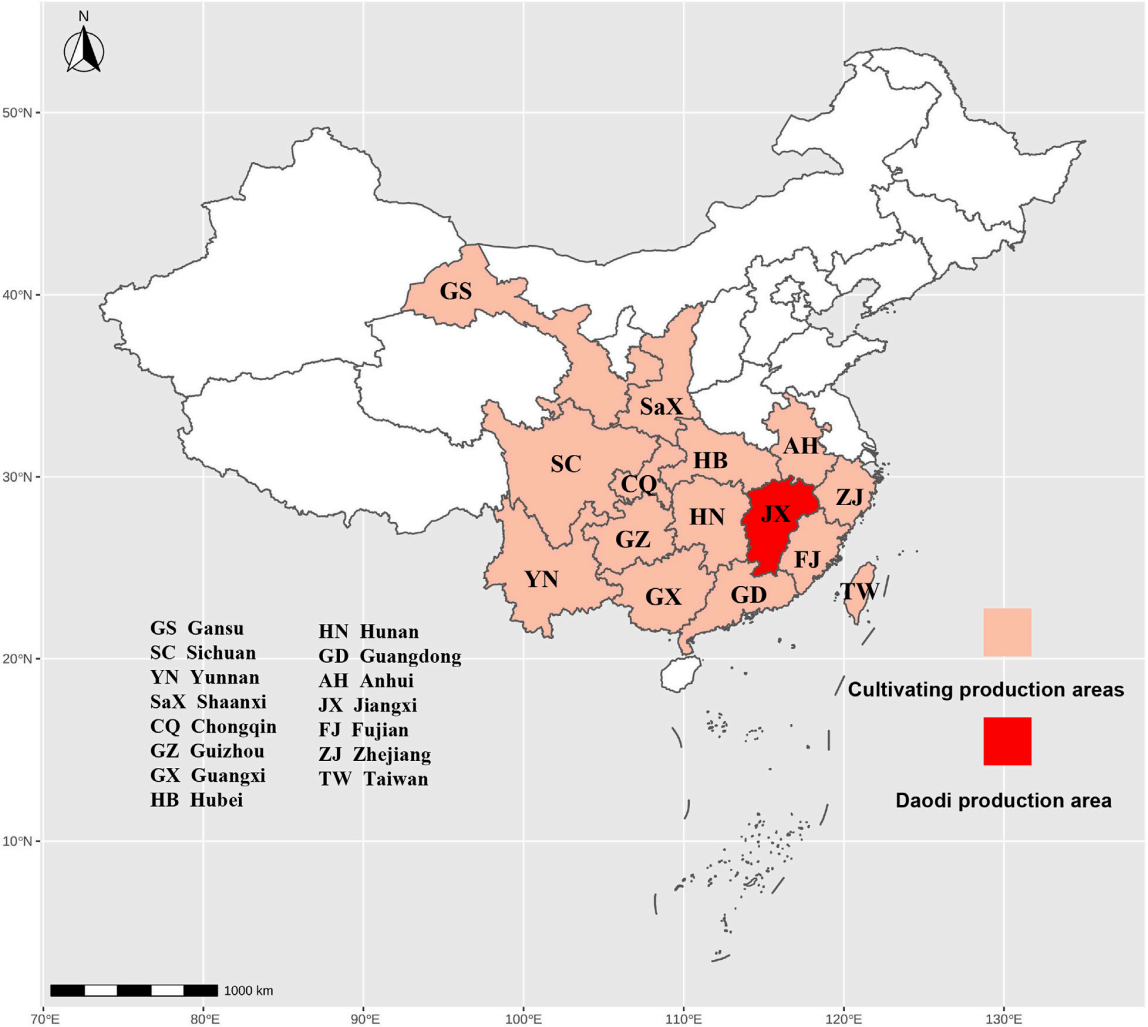
Cultivation and distribution of *Euodia Rutaecarpa* in China.

*Euodia rutaecarpa* cultivation began at the end of the Eastern Han Dynasty. *Miscellaneous Records of Famous Physicians* (Wei and Jin Dynasties, A.D. 220–450) first recorded that “*Euodia rutaecarpa* is grown in the valley, picked on September 9, kept cool and dry, and kept as long as possible”. EF had become a widely used medicine during the Tang Dynasty, with renowned poet Wang Wei noting that *Euodia rutaecarpa* could be seen throughout mountains during the Double Ninth Festival. Contemporary research indicates that *Euodia rutaecarpa* thrives in sunlit, warm environments, typically flourishing in thinly spread forests or shrubs in mountainous areas ranging from flat to 1,500 m above sea level, predominantly on sunlit inclines. It is relatively cold resistant, but in cold, windy, and dry areas in winter, and in areas with many diseases, the results are low, and the growth is poor. *Illustrated Classic of Materia Medica* (Song Dynasty, A.D. 960–1,279) recorded that “*Euodia rutaecarpa* can be found everywhere, especially in Jiangsu, Zhejiang, and Sichuan. Jiangxi is EF’s the authentic origin, rich in high-quality MEF. It is distributed in the north and south of Jiangxi Province, mainly in urban Zhangshu, Fengcheng, Gaoan, Xingan, Xiajiang, Xinyu, and Jishui. As clinical needs swiftly rose, numerous provinces introduced *Euodia rutaecarpa*. Now, it is distributed in the south of the Qinling Mountains in China, mainly in Guizhou, Guangxi, Hunan, and Yunnan Provinces (Figure 2). Nowadays, *Euodia rutaecarpa* is predominantly thrived in Asia, East Africa, and Oceania, with extensive cultivation in ancient Japan and Korea. *Euodia rutaecarpa* was introduced in Korea during The Three Kingdoms Period, and the *Goryeo Master Fang* (Wei and Jin Dynasties, A.D. 220–420) recorded the treatment of beriberi with EF (Nam et al., 2016). *Euodia rutaecarpa* was introduced in Japan during the Edogawa period. EF and its namesake, Goshuyuto (known as Wuzhuyu decoction in Chinese), are frequently utilized in clinical settings (Hibino et al., 2009a).

## 3 Traditional applications

### 3.1 The drug application of EF

EF was first recorded in *Shen Nong’s Herbal Classic*, which records the nature, taste, meridian tropism, and efficacy of EF, laying the foundation for the modern clinical application of EF. Over time, the therapeutic impact of EF has evolved through extensive research. It is pointed out that EF can treat many diseases, such as Jueyin headache, hernia, abdominal pain, beriberi, vomiting blood, acid regurgitation, and diarrhea (Table 2). Concurrently, *Miscellaneous Records of Famous Physicians* initially documented EF’s mild toxicity, with ongoing research enhancing EF’s toxicology. *Illustrated Classic of Materia Medica* recorded that EF harms the eyes and hair. *Amplified Herbology* (Song Dynasty, A.D. 960–1,279) recorded that EF damages the intestines and stomach. *Correlation between Materia Medica Companion* (Ming Dynasty, A.D. 1,368–1,644) recorded that EF damages the healthy atmosphere. Mouth ulcers, tongue sores, and dizziness caused by excessive consumption of EF are recorded in *Compendium of Materia Medica* (Ming Dynasty, A.D. 1,368–1,644). The ChP recorded the minor toxicity of EF and stipulated that the dosage of EF was 2–5 g.

**TABLE 2:** The traditional uses of EF in China.

Traditional uses	Dynasty/Years	References
Warming the middle to dispel colds, relieve cough, remove dampness, and stop arthralgia	Eastern Han Dynasty	*Shen Nong’s Herbal Classic* 《神农本草经》
Dispelling phlegm, relieving heartache, and reconciling the five internal organs	Wei and Jin Dynasties	*Miscellaneous Records of Famous Physicians* 《名医别录》
Killing insects and warming Yang	Tang Dynasty	*A Supplement to Materia Medica* 《本草拾遗》
Warming the spleen, promoting digestion and defecation. Treatment of cold pain in the heart and abdomen, vomiting and diarrhea	Tang Dynasty	*Theory of Medicinal Properties* 《药性论》
Strengthening the spleen, promoting joint function, eliminating phlegm, dispelling wind, treating abdominal pain, athlete’s foot, edema, and postpartum blood stasis	Five Dynasties	*Rihuazi Bencao* 《日华子本草》
Relieving sore throat and chest-relaxing	Jin and Yuan Dynasties	*Properties and Actions of Medicinals* 《药类法象》
Relieving heart, abdominal pain and alcohol	Jin and Yuan Dynasties	*Medical Origins* 《医学启源》
Treatment of acid regurgitation, abdominal pain, hernia, dysentery, mouth sores	Ming Dynasty	*Compendium of Materia Medica* 《本草纲目》
Relieving cough, expelling wind, eliminating food, dispelling arthralgia	Ming Dynasty	*Explain of Medicinal Properties* 《药性解》
Moistening the liver, invigorating the spleen, relieving depression, removing phlegm, killing insects, and dispelling cold. Treatment of Jueyin headache, hemorrhoids, and dysentery	Qing Dynasty	*Bencao Beiyao* 《本草备要》
Descending qi, dispelling cold, eliminating abdominal distension. Treatment of acid regurgitation, diarrhea, abdominal pain, beriberi, edema, and aphtha	Qing Dynasty	*Bencao Qiuzhen* 《本草求真》
Dispelling cold, tonifying the lung, relieving pain, activating blood circulation, and dispelling arthralgia	Qing Dynasty	*Notes on Shen Nong’s Herbal Classic* 《神农本草经读》
Warming the stomach, dispelling cold, relieving pain, stopping cough, promoting blood circulation	Qing Dynasty	*Record of One Hundred Species of Shen Nong’s Herbal Classic* 《神农本草经百种录》
Treatment of Jueyin headache, hernia, abdominal pain, beriberi, hematemesis, acid regurgitation, and diarrhea	2020	Chinese pharmacopoeia 《中国药典》

### 3.2 The classic prescription application of EF

EF has been a staple in clinical prescriptions since antiquity (Table 3). Wuzhuyu decoction, named after the monarch medicine EF in *Treatise on Febrile Diseases* (Dong Han Dynasty, A.D. 25–220), has the effect of warming the middle to replenish deficiency, lowering qi and stopping vomiting. It was the earliest record of the use of EF in clinical treatment. *Synopsis of the Golden Chamber* (Dong Han Dynasty, A.D. 25–220) mentioned twice the EF of warming channels, dispelling cold, and stopping vomiting, which is an important medicine for tonifying the spleen and stomach. Among the over 5,000 prescriptions in *Thousand-Gold Essential Formula for Emergency* (Tang Dynasty, A.D. 618–907), there were 143 prescriptions mentioned EF. The Essential Secrets from the *Imperial Library* (Tang Dynasty, A.D. 618–907) had over 6,000 prescriptions, and the number of prescriptions contained EF reached 176. *Formula of Peaceful Beneuolence Pharmacy* (Song Dynasty, A.D. 1,078–1,085) was the first official preparation standard. This book recorded a total of 788 prescriptions, and 13 referred to EF. In the clinical realm, essential formulas featuring EF encompass the Wuzhuyu Decoction, Zuojin Pill, Wenjing Decoction, and Sishen Pill, among others.

**TABLE 3:** Traditional application of EF in classic prescriptions.

Classification	Formula name	Main compositions	Dosage form	• Traditional efficacy ▪ Clinical applications	EF’s function	References
Classic prescription	Wuzhuyu Tang	Euodiae Fructus, Ginseng Radix Et Rhizoma, Zingiberis Rhizoma Recens, Jujubae Fructus	Decoction	• Tonifying deficiency in warming and stopping vomiting ▪ Chronic gastritis, nervous vomiting, and otogenic vertigo	Warming liver for dispelling cold	*Treatise on Febrile Diseases* 《伤寒论》
	Wenjing Tang	Euodiae Fructus, Cinnamomi Ramulus, Angelicae Sinensis Radix, Chuanxiong Rhizoma, Moutan Cortex, Asini Corii Colla, Paeoniae Radix Alba, Ophiopogonis Radix, Ginseng Radix et Rhizoma, Glycyrrhizae Radix et Rhizoma, Pinelliae Rhizoma, Zingiberis Rhizoma Recens	Decoction	• Warming channels and dispelling cold ▪ Functional uterine bleeding and chronic pelvic inflammatory	Disperse cold and alleviate pain	*Synopsis of the Golden Chamber* 《金匮要略》
	Wu Ji Wan	Coptidis Rhizoma, Euodiae Fructus, Paeoniae Radix Alba	Pill	• Soothing liver and regulating spleen ▪ Abdominal pain, stomachache, and chronic diarrhea	Harmonizing stomach for descending adverse qi	*Formula of Peaceful Beneuolence Pharmacy* 《太平惠民和剂局方》
	Ai Fu Nuan Gong Wan	Artemisiae Argyi Folium, Cyperi Rhizoma, Euodiae Fructus, Cinnamomi Cortex, Angelicae Sinensis Radix, Chuanxiong Rhizoma, Paeoniae Radix Alba, Rehmanniae Radix, Astragalus Radix, Dipsaci Radix	Pill	• Warming uterus and regulating menstruation ▪ Irregular menstruation, and dysmenorrhea	Warming channel, dispelling cold and promote blood circulation	*Standards for Diagnosis and Treatment* 《仁斋直指方论》
	Zuo Jin Wan	Coptidis Rhizoma, Euodiae Fructus	Pill	• Purging fire and soothing the liver ▪ Esophagitis, gastritis and peptic ulcer	Lowering adverse flow of qi and arresting vomiting	*Danxi’s Mastery of Medicine* 《丹溪心法》
	Sishen Wan	Psoraleae Fructus, Euodiae Fructus, Myristicae Semen, Schisandrae Chinensis Fructus, Jujubae Fructus	Pill	• Warming the kidney and dispelling cold ▪ Chronic diarrhea and irritable bowel syndrome	warming spleen and stomach for dispelling cold	*Standards for Diagnosis and Treatment* 《证治准绳》
	Ji Ming San	Arecae Semen, Citri Reticulatae Pericarpium, Chaenomelis Fructus, Euodiae Fructus, Platycodonis Radix, Zingiberis Rhizoma Recens, Perillae Folium	Decoction	• Regulating the qi flowing in the channels ▪ Beriberi and rheumatoid arthritis	dissipating cold and eliminating dampness	*Standards for Diagnosis and Treatment* 《证治准绳》
TCM preparation	Changkang Tablets	Berberine hydrochloride, Euodiae Fructus, Aucklandiae Radix	Pill	• Clearing heat and dampness, regulating qi and relieving pain ▪ Diarrhea, dysentery and abdominal pain	dissipating cold and eliminating dampness	Chinese pharmacopoeia 《中国药典》
	Huatuo Zaizao Wan	Concentrated water-honeyed pill composed of Chuangxiong Rhizoma, Euodiae Fructus, Borneolum Syntheticum, etc	Pill	• Promoting blood circulation, resolving phlegm and dredging collaterals ▪ Stroke and its sequelae	Warming channel, dispelling cold and move qi to relieve pain	Chinese pharmacopoeia 《中国药典》
	Jiawei Zuo Jin Wan	Coptidis Rhizoma, Euodiae Fructus, Scutellariae Radix, Bupleuri Radix, Aucklandiae Radix, Cyperi Rhizoma, Curcumae Radix, Paeoniae Radix Alba, Citri Reticulatae Pericarpium Viride, Aurantii Fructus, Citri Reticulatae Pericarpium, Corydalis Rhizoma, Angelicae Sinensis Radix, Radix Glycyrrhizae Preparata Radix et Rhizoma	Pill	• Soothing the liver and relieving depression to relieve pain ▪ Acute or chronic hepatitis or gastritis	Lowering adverse flow of qi and arresting vomiting	Chinese pharmacopoeia 《中国药典》
	Compound Berberine Tablets	Berberine hydrochloride, Euodiae Fructus, Aucklandiae Radix, Paeoniae Radix Alba	Pill	• Heat-clearing, damp-drying, stopping dysentery and diarrhea ▪ Acute gastroenteritis, dysentery and chronic diarrhea	Warming channel, dispelling cold and promote blood circulation	Chinese pharmacopoeia 《中国药典》

In addition to examining EF in traditional medical texts, the study of EF in botanical drugs has been extensively explored in contemporary medical settings. Wuzhuyu Decoction can treat chronic non-atrophic gastritis (Hu et al., 2023), chronic migraine (Pan et al., 2015; Nan et al., 2022), alcoholic gastric ulcer (Wang X. et al., 2023), and atherosclerosis (Li C. et al., 2022). Zuojin Pill has pharmacological effects such as anti-tumor (Peng et al., 2015), protection of gastric mucosa, anti-inflammation, anti-ulcer, and so on (Wang et al., 2015; Zhang J. et al., 2022). It can treat bile reflux gastritis (Li Y. Y. et al., 2022) and septic lung injury (Yin et al., 2021). Wenjing Decoction can treat primary dysmenorrhea (Gao et al., 2017) and endometriosis (Huang et al., 2023). Sishen Pill has anti-inflammatory and anti-tumor pharmacological effects (Zhang B. et al., 2024), can treat abdominal pain, diarrhea (Li et al., 2024), irritable bowel syndrome (Zhang X. Y. et al., 2021; Zhao et al., 2024), and insomnia (Wang L. X. et al., 2023). EF is recommended as the primary treatment in Japan for ailments like cold headaches, dysmenorrhea, and inflammatory pain in rheumatoid joints (https://www.mhlw.go.jp/index.html). Certain EF-containing prescriptions, including Changkang Tablets, Huatuo Zaizao Wan, Compound Berberine Tablets, and Jiawei Zuo Jin Wan, have undergone extensive research and clinical application. Changkang Tablets are used in the treatment of dysentery, abdominal pain, and tenesmus (Shi et al., 2013; Guan et al., 2020), Huatuo Zaizao Wan can treat Alzheimer’s disease (Jiang et al., 2023) and stroke (Duan et al., 2017).

## 4 Phytochemistry of EF

Presently, about 300 metabolites of EF have been isolated and purified, which are mainly divided into alkaloids (1–148), terpenoids (149–184), flavonoids (185–213), volatile oils (214–283), and others (284–299). These metabolites are summarized in Supplementary Table S1, and their structures are shown in Supplementary Figures S1–S8. The alkaloids are primarily categorized into indoles and quinolones (Li D. W. et al., 2020). Evodiamine and rutaecarpine in indole are the index metabolites of EF. The limonin in terpenoids plays a crucial role in EF.

### 4.1 Alkaloids

Alkaloids are the metabolites of EF, mainly composed of indoles (Supplementary Figure S1) and quinolones (Supplementary Figure S2). Alkaloids fundamentally possess a circular form and are non-soluble in water, resulting in a higher concentration of EF alkaloids in ethanol. Indoles are synthesized mainly through methanesulfonic acid and amino acids. Over ten different compounds, including evodiamine, rutaecarpine, and dehydroevodiamine, were extracted from EF (Zuo et al., 2000; Wang Q. Z. et al., 2010; Wang T. Y. et al., 2010; Wang X. X. et al., 2013; Li et al., 2014; Zhao N. et al., 2015; Ma et al., 2021; Qin et al., 2021; Zhao X. M. et al., 2021). Evodiamine and rutaecarpine are the most important metabolites, and their contents are also the highest in EF (Huang et al., 2019; Li D. W. et al., 2020). Dihydroevocarpine and evocarpine are the crucial metabolites of quinolones found in EF (Li Y. H. et al., 2020). Furthermore, the group includes quinolines, organic amines, acridone, and purines (Minh et al., 2003; Kim et al., 2022) (Supplementary Figure S3). Research revealed a reduction in the levels of evodiamine, rutaecarpine, and carpine in EF correlating with the fruit’s diminution. Some commercial evodiamine and rutaecarpine in SEF are below the content specified in ChP (Cao et al., 2019).

### 4.2 Terpenoids

Typically, terpenoids (Supplementary Figure S4) originate from methylpentanedioic acid, with isoprene forming the fundamental structural component of the molecular framework (Zhao H. et al., 2021; Qian et al., 2014). Limonin is an oxidized tetracyclic triterpene with a distinctive furan ring, and it is a terpenoid extracted and recognized from EF. They constitute the material basis of the bitter properties of EF (Bae et al., 2020). Its representative metabolites are limonin and rutaevine, and limonin is another index metabolite of EF. In addition, there are high contents of metabolites, such as evodol, obacunone, rutaevine acetate, 6β-acetoxy-5-epillimonin, jangomolide, and shihulimonin A (Lacroix et al., 2011). Due to their significant solubility in fats, terpenoids are typically processed using an ethanol solvent. Research indicates that limonin levels in SEF and MEF are notably elevated, approximately 0.74% and 0.65%, respectively, in contrast to LEF’s mere 0.24% limonin content (Zhang et al., 2021c).

### 4.3 Flavonoids

Flavonoids (Supplementary Figure S5) generally refer to a series of metabolites formed by connecting two benzene rings with three carbon atoms. EF additionally has a higher content of flavonoids. It mainly includes these three metabolites: flavonoids and their glycosides, flavonols and their glycosides, and flavonones and their glycosides. Flavonoids and their glycosides are predominantly associated with *O*-glucose, *O*-xylose, *O*-galactose, *O*-rhamnose, *O*-rue sugar, and *O*-mulberry disaccharide. Flavonols and their glycosides mainly include quercetin, isorhamnetin, limocitrin glycosides, and aglycones (Xiao et al., 2023; He et al., 2024). Flavonones and their glycosides are phellodensin F, catechin, and hesperidin, respectively (Zhao Z. et al., 2015; Li and Wang, 2020).

### 4.4 Volatile oil

EF has a strong and fragrant smell because of its high content of volatile oil (Supplementary Figure S6). The volatile oil metabolites are monoterpene, sesquiterpene, aliphatic, and aromatic. Within the isolated volatile oil, the proportions of sesquiterpenes exceed 38%, monoterpenes surpass 35%, and esters exceed 13% (Liu S. S. et al., 2019). Furthermore, while EF volatile oil exhibits significant pharmacological properties, it simultaneously constitutes a toxic metabolite of EF. Its metabolites are monoterpenoids, such as myrcene and (*E*)-ocimene, and sesquiterpenes, such as β-caryophyllene and β-elemene.

### 4.5 Others

EF also contains some organic acids (Supplementary Figure S7), including caffeic acid, citric acid, isocitric acid, trans-caffeoylgluconic acid, and feruloylgluconic acid. EF also contains phenylpropanoids, mainly divided into simple phenylpropanoids, coumarins, and lignans, most of which belong to simple phenylpropanoids. Simple phenylpropanoids include p-hydroxycinnamic acid, ferulic acid, coniferin, chlorogenic acid, etc. Besides the metabolites mentioned above, EF also contains anthraquinones, such as chrysophanol, emodin, and physcion, and steroids, such as β-sitosterol, β-daucosterol, and β-stigmasterol (Supplementary Figure S8).

## 5 Processing

### 5.1 Traditional processing methods

**FIGURE 3:**
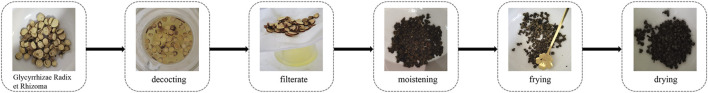
Flow chart of processing EF with licorice.

Processing is the essence of TCM application, which can increase the efficacy and reduce the toxicity of drugs. EF’s processing boasts an extensive historical background, with raw EF typically exerting a significant influence in heating the spleen and eliminating cold. The long traditional technology of processing EF with licorice is pointed out in the *Synopsis of the Golden Chamber*. *Master Lei’s Treatise on Drug Processing* (Northern and Southern Dynasties, A.D. 420–479) recorded that EF is processed with salt to enhance the analgesic effect and vinegar to correct the taste. *Materia Medica for Dietotherapy* (Tang Dynasties, A.D. 618–907) recorded that EF could enhance the antiemetic effect after processing ginger and the analgesic effect after being processed with yellow rice wine. *General Records of Holy Universal Relief* (Song Dynasties, A.D. 1,078–1,085) recorded that EF can reduce toxicity when processed with soybean products. *Prescriptions for Universal Relief* (Ming Dynasty, A.D. 1,368–1,644) recorded that EF fried with Psoraleae Fructus can enhance the antidiarrheal effect. *Wonderful Well-Tried Recipes* (Ming Dynasty, A.D. 1,368–1,644) recorded that EF processed with Coptidis Rhizoma can enhance the antiemetic effect. The process and method of processing EF with licorice are described in detail in ChP (Figure 3). In addition, different doses of licorice can affect EF’s chemical composition and pharmacological effects (Xiao et al., 2012). Certain academics have experimented with varying the EF to licorice dosage ratios, discovering the optimal 100:6.

### 5.2 Enhance efficiency and reduce toxicity

In its extended clinical application, TCM has developed distinct theories and techniques for detoxifying and improving its healing impact, encompassing both processing and compatibility (Li R. L. et al., 2021). Special processing techniques such as licorice, salt, ginger, and vinegar can reduce the toxicity of EF. The ChP stipulates that EF ought to be fried alongside licorice. Some scholars have found that processing EF with licorice can reduce the toxicity of alkaloids (Ren et al., 2023a). The cytochrome P450 (P450, CYP) enzyme activates evodiamine in EF during metabolism, which leads to liver injury and inflammation. Licorice can reduce the toxicity of EF by inhibiting the P450 enzyme and blocking the metabolic activation of EF (Ren et al., 2024). Licorice can obstruct EF protein coupling by suppressing the P450 enzyme, elevating GSH levels in human liver cells, and mitigating the GSH reduction induced by EF (Ren et al., 2023b). Salt-processed EF can introduce drugs into the kidney channel, reduce toxicity, and ensure the safety of clinical drug use (Hou et al., 2023). When EF is combined with ginger, its antiemetic properties can be amplified. Some studies found that EF processed with ginger, licorice, and salt had better antitoxic effects (Zhang M. et al., 2021). Other studies found that the three processing methods of ginger, licorice, and vinegar could reduce the content of rutaecarpine in EF, with vinegar processing increasing the content of evodiamine, and licorice processing had the most significant effect on reducing toxicity (Li H. et al., 2021). The metabolites of EF obtained by different processing methods, such as stir-frying, roasting, and steaming, are also different (Xiao et al., 2023). A comparative study of various EF-processed products revealed that the combined amounts of evodiamine, rutaecarpine, and evodol in EF for stir-frying exceed those in baking and cooking.

EF combined with other drugs can also counteract its toxicity. For example, the toxicity of EF significantly decreased when EF was used in combination with licorice and jujube (He et al., 2024). EF with Coptidis Rhizoma can enhance the anti-inflammatory effect and inhibit the inflammatory reaction in RAW264.7 cells by significantly reducing the levels of IL-6, TNF-α, and IL-1β (Wang J. et al., 2024). Additionally, it is capable of markedly reducing apoptosis and enhancing the defensive role of the gastric mucosa through the suppression of gastric acid release (Zhang Z. et al., 2024). Berberine is an important metabolite of Coptidis Rhizoma, which can counteract the side effects of evodiamine and reduce the risk of evodiamine in treating gastric cancer (Shi et al., 2013). The combination of berberine and evodiamine can synergistically inhibit the proliferation of human breast cancer cells by inducing cell cycle arrest and apoptosis (Du et al., 2017). The researchers found that EF combined with ginger, Citri Reticulatae Pericarpium, Paeoniae Radix Alba, and Angelicae Sinensis Radix can improve the efficacy of EF (Wu et al., 2021).

## 6 Quality control

### 6.1 Quality standard of EF in ChP

With the continuous updating of the edition of ChP, the identification, content evaluation, testing technology, and quality standards of EF are constantly improving (Table 4). EF was first recorded in the 1963 edition of ChP, and it was clearly recorded that EF was processed with licorice. Then, the identification method of EF appeared for the first time in the 1973 edition of ChP. In the 1985 edition of ChP, the EF identification method was officially determined as hydrochloric acid filtration, potassium mercuric iodide de-precipitation, and the formation of a reddish-brown ring zone at the interface between dimethylaminobenzaldehyde and EF solution. The ChP has been improving the quality control of EF since 2000. For the first time, the determination method and the lowest value of EF appeared in the 2000 edition of ChP, which stipulates that the total amount of evodiamine and rutaecarpine should not be less than 0.2%. Next, the total amount of evodiamine and rutaecarpine should not be less than 0.15% in the 2005 edition of ChP. Limonin content is adjusted to no less than 1.0% in the 2010 edition of ChP. Limonin content is again adjusted to no less than 0.20% in the 2015 edition of ChP. The 2015 edition of ChP is basically consistent with the 2020 edition of ChP, indicating that the metabolites of EF are relatively stable and can better represent the efficacy of EF. However, the maximum reference dose of metabolites is not specified in ChP, and the toxicity of EF has not been reasonably controlled.

**TABLE 4:** Changes of content indexes and limits of EF in ChP.

Edition	Fluidity	Wavelength	Quality markers	Content requirements
2000	Acetonitrile-water-tetrahydrofuran-acetic acid (51:48:1:0.1)	225 nm	Evodiamine	≥0.20%
			Rutaecarpine	≥0.20%
2005	Acetonitrile-0.04% sodium octane sulfonate (43:57)	225 nm	Evodiamine	≥0.15%
			Rutaecarpine	≥0.15%
2010	Acetonitrile-water-tetrahydrofuran-glacial acetic acid (41:59:1:0.2)	225 nm	Evodiamine	≥0.15%
			Rutaecarpine	≥0.15%
			Limonin	≥1.00%
2015/2020	[Acetonitrile-tetrahydrofuran (25:15)]-0.02% phosphoric acid (35:65)	215 nm	Evodiamine	≥0.15%
			Rutaecarpine	≥0.15%
			Limonin	≥0.20%

### 6.2 Exploration of modern quality control

TCM’s effectiveness can differ significantly based on the type of plant, its source, and yield. Diverse environmental factors like soil, air quality, precipitation, and sunlight play a crucial role in shaping EF growth, with each EF metabolite varying in content across regions (Liu Y. et al., 2019). Some studies have shown that the metabolite of EF varies greatly in different locations, and there is little change in the metabolite of EF in different years in the same location (Huang et al., 2008; Zhou et al., 2010). By comparing the EF of different batches of LEF, MEF, and SEF, it was found that the limonin content in SEF was higher than that of MEF and LEF, while the alkaloid content was higher in MEF and LEF. The toxicity of EF is related to the content ratio of limonin and alkaloid. The higher the ratio, the lower the toxicity of EF. In other words, MEF is more toxic than other categories, and SEF is less toxic than other categories (Zhang et al., 2021b). Furthermore, certain academics analyzed the four vital metabolites of ER, ERO, and ERB, namely evodiamine, evodiamine, dehydroevodiamine, and narcissoside, discovering comparable levels of dehydroevodiamine. The content of evodiamine, rutaecarpine, and narcissoside was the highest in ERO and the lowest in ERB (Li C. H. et al., 2021).

The ChP stipulated the detection method of EF and pointed out that evodiamine, rutaecarpine, and limonin are the crucial metabolites of EF. However, these three metabolites do not represent the overall pharmacological effects of EF. Consequently, sophisticated detection techniques are essential for qualitative and quantitative analysis of metabolites in EF. The leading analytical technologies include TLC, HPLC, HPLC-MS, GC-MS, CE, and CCC (Xia et al., 2023). At present, there are many studies on the metabolites of EF. Scholars have isolated three metabolites from EF: rutaecarpine, evodiamine, and evodiamide (Zhou et al., 2006). Then, two new-lactone derivatives, evodinoids A and B, and a new volatile oil, are separated from EF (Xin et al., 2022). In addition, five metabolites of EF were found. They are limonin, 1-methyl-2-undecyl-4(1H) quinolone, evocarpine, 1-methy-2-[(6Z,9Z)]-6,9-pentadecadienyl-4-(1H)-quinolone, and dihydroevocarpine (Zhang et al., 2013). It has been proven that evodiamine is closely related to the hepatotoxicity of EF (Zhang et al., 2021b). Limonin serves as the primary liver-protective metabolite of EF, while evodiamine is the chief liver-damaging metabolite of EF. Subsequently, eleven critical metabolites of EF underwent analysis using non-specific metabonomics and *in vitro* functional techniques (Yong et al., 2024). Up to this point, a total of 17 metabolites have undergone screening from EF, including neochlorogenic acid, caffeic acid, chlorogenic acid, 3-*O*-feruloylquinic acid, hyperoside, quercetin-3-*O*-sambubioside, rutin, dehydroevodiamine, isorhamnetin-3-*O-*β*-D*-galactoside, narcissin, isorhamnetin-3-*O-*β*-D*-glucopyranoside, diosmin, rutaevine, limonin, evodiamine, rutaecarpine, and evocarpine.

## 7 Pharmacological effects

EF is a classical plant medicine with various pharmacological effects, such as cardiovascular protection, gastrointestinal protection, neuroprotection, anti-inflammation, analgesia, anti-tumor, glucose and lipid metabolism regulation, etc. (Supplementary Table S2). Evodiamine, rutaecarpine, and limonin serve as the indicator metabolites of EF but are also abundant in phytochemistry and pharmacology, which are often used to treat diseases of the immune, nervous, digestive, circulatory, and endocrine systems. Elucidating the pharmacological effects of EF is the key to guiding the rational clinical application of drugs and ensuring the curative effect.

### 7.1 Cardiovascular protection

EF has a cardiovascular protective effect, and its aqueous extract can contract the aorta (Hibino et al., 2009b). In managing cardiovascular conditions, evodiamine, rutaecarpine, and limonin serve as crucial metabolites of EF, offering protection against myocardial ischemia-reperfusion (I/R), anti-myocardial fibrosis, anti-arrhythmia, safeguarding vascular endothelial damage, altering vascular tension, and so on. Studies have shown that evodiamine has an anti-atherosclerotic effect, can regulate energy by inhibiting the expression of the β1-adrenergic receptor, and prevents cardiac I/R injury (Xue et al., 2015). Subsequently, evodiamine has the ability to control the growth and movement of vascular smooth muscle cells by blocking the PI3K/AKT axis activation in the traditional route, thereby preventing atherosclerosis onset and progression (Zha et al., 2023). What’s more, evodiamine can prevent isoproterenol-induced cardiac fibrosis by regulating endothelial-to-mesenchymal transition (Huang et al., 2017). Moreover, rutaecarpine promotes endothelial nitric oxide synthase (eNOS) phosphorylation and NO synthesis via the Ca^2+^/calmodulin-dependent protein kinase II (CaMKII) and calmodulin-dependent protein kinase kinase β (CaMKKβ)/AMP-activated protein kinase (AMPK) signaling pathways through transient receptor potential vanilloid type 1 (TRPV1), and effectively prevent endothelial dysfunction (Lee et al., 2021). Rutaecarpine can also reduce the damage to myocardial cells caused by myocardial infarction by enhancing vascular smooth muscle calcification (Zhan et al., 2021). Some studies have shown that limonin can inhibit adriamycin-induced cardiotoxicity by activating Nrf2 and SIRT2 signal pathways (Li X. H. et al., 2022). Limonin can also inhibit the ubiquitination and degradation of SIRT6, stabilize the level of SIRT6 protein, promote its expression, reduce cardiac hypertrophy, and improve cardiac function (Liu et al., 2022).

### 7.2 Gastrointestinal protection

EF is an effective botanical drug for treating gastrointestinal diseases, especially evodiamine, rutaecarpine, and dehydroevodiamine (Chen et al., 2023). Evodiamine can act as an antioxidant by blocking the Rho/NF-κB pathway and alleviating gastric mucosal injury (Zhao Z. et al., 2015). It can also inhibit gastritis caused by *Helicobacter pylori* infection by inhibiting the NF-κB pathway (Yang et al., 2021). Then, evodiamine can effectively improve the imbalance of intestinal microflora and relieve the symptoms of ulcerative colitis by increasing the level of *lactobacillus* acidophilus and the production of acetate (Wang et al., 2020). In addition, it can suppress gastrointestinal hyperactivity caused by stress via cholecystokinin (CCK) and the CCK1 receptor (Ren et al., 2018). Moreover, rutaecarpine is effective in mitigating gastric damage caused by ethanol through the suppression of NF-κB pathway anti-inflammation, Nrf2 pathway antioxidation, stimulation of Bcl-2, suppression of Bax and caspase-3 expression, and prevention of gastric cell apoptosis (Ren et al., 2020). Some studies have shown that TRPV1/calcitonin gene-related peptide (CGRP) pathway is an important therapeutic target for gastric mucosal injury (Luo et al., 2013). Rutaecarpine can stimulate the TRPV1 receptor to release CGRP, inhibit the excessive secretion of gastric acid, and improve the symptoms of gastric ulcers (Liu et al., 2008). Moreover, Dehydroevodiamine can reduce the inflammatory injury of gastric mucosa by reducing the ERK/p38 signal pathway, down-regulating the expression of myeloperoxidase (MPO), TNF-α and IL-6, upregulating the expression of IL-10, regulating gastric pH and mucosal thickness (Wei et al., 2021). Then, dehydroevodiamine can also improve MNNG-induced gastric mucosal injury and GES-1 migration in chronic atrophic gastritis rats and treat atrophic gastritis by inhibiting hypoxia-inducible factor 1α/vascular endothelial growth factor angiogenesis pathway (Wen et al., 2021). Furthermore, EF polysaccharides have a protective effect on gastric mucosa and can alleviate the symptoms of gastric ulcers. By increasing the expression of Nrf2 and HO-1 protein, reducing the expression of Keap1 protein, activating Keap1/Nrf2/HO-1 signal pathway, and reducing oxidative stress in the stomach (Luo et al., 2023).

### 7.3 Neuroprotection

The application of EF in neurological disorders is becoming increasingly widespread. EF and its metabolites have neuroprotective effects on neurodegenerative diseases such as ischemic injury, neuropathic pain, neuroinflammation, Alzheimer’s disease (AD), and so on. Studies have shown that EF methanol extract (200 mg/kg) protects neurons and prevents ischemia-induced cognitive impairment (Lee et al., 2011). Then, evodiamine can reduce peripheral hypersensitivity and anxiety in nerve-injured mice (Zhang et al., 2020). Evodiamine can also repair memory and cognitive impairment, protect neurons *in vitro*, and inhibit glial cell activation and neuroinflammation (Lima and Hamerski, 2019). In the experimental AD mice induced by intracerebroventricular injection of streptomycin, evodiamine (50 and 100 mg/kg) was orally given daily for 21 days, the ability to recognize new targets and the score of water maze test was improved in AD mice (Wang et al., 2018a). In addition, evodiamine can improve AD mice’s learning and cognitive impairment (Wan et al., 2024) and treat AD through antioxidation and anti-apoptosis (Zhang et al., 2018). Moreover, rutaecarpine ameliorates neuronal injury in rats with cerebral I/R by regulating the expression of ERK1/2 and Nrf2/HO-1 pathway (Han et al., 2019). Rutaecarpine can affect Ca^2+^ influx and activate PI3K/AKT signal pathway by activating specific capsaicin receptor TRPV1, inhibit intracellular oxidative stress and apoptosis protease activity, and protect neurons from apoptosis induced by hypoxia-reoxygenation (Yang Y. et al., 2018). Additional research has shown that limonin and its variants are versatile in combating neuroinflammation and neuronal apoptosis by activating PI3K/AKT and reducing TLR4/NF-κB pathway activity and are effective in treating AD (Panda S. P. et al., 2024). Limonin plays a neuroprotective role by inhibiting neuronal autophagy and microglial activation in rats injected with 6-hydroxydopamine (Gao et al., 2023). Furthermore, dehydroevodiamine (10 mg/kg) can improve the symptoms of memory impairment induced by scopolamine in mice. Dehydroevodiamine has a strong protective effect on cognitive impairment through its antioxidant activity, inhibition of neurotoxicity, and intracellular calcium. Therefore, Dehydroevodiamine may be an important drug for treating memory disorders (Shin et al., 2017). Tg2576-induced AD mice were treated with dehydroevodiamine (0.5 mg/kg) for 4 months, which improved the memory impairment of Tg mice and decreased the levels of soluble amyloid-β 40 (Aβ40), soluble Aβ42 and total Aβ peptide in the cortex of Tg mice. Dehydroevodiamine can inhibit the activity of β-secretase in a dose-dependent manner, which is related to the production of Aβ and the formation of neuritis plaques. Dehydroevodiamine may have a therapeutic effect on AD as a β-secretase inhibitor (Shin et al., 2016).

### 7.4 Anti-inflammation

The anti-inflammatory activity of EF has been widely recognized. Research indicates that the 70% ethanol extract of EF can inhibit the inflammatory response in HaCaT cells. It exerts its anti-inflammatory effects by modulating the JAK-STAT and MAPK signaling pathways. This regulation suppresses inflammatory mediators, cytokines, and chemokines, alleviating symptoms associated with atopic dermatitis (Jin et al., 2024). EF has a potent anti-inflammatory and uric acid-lowering effect. The EF water extract can significantly improve the production of serum inflammatory cytokines IL-1β and TNF-α and inhibit the activation of renal NLRP3 inflammatory signal (Wang Z. et al., 2024). In addition, rutaecarpine can significantly reduce the inflammatory response induced by pseudotype severe acute respiratory syndrome coronavirus 2 by blocking the activity of 3C-like protease (Lin et al., 2023). It has been proved that rutaecarpine can inhibit inflammation by inhibiting the NF-κB signal pathway mediated by PI3K/AKT and MAPK and reduce lipopolysaccharide (LPS)-induced cell migration and number by inhibiting Src/FAK pathway (Jayakumar et al., 2021). Rutaecarpine has also reduced inflammatory responses by downregulating interferon-α, IL-23 p19, and IL-17A protein. This anti-inflammatory effect is mediated through the NF-κB and TLR7 signaling pathways (Li Y. et al., 2019). Moreover, evodiamine is anti-inflammatory by inhibiting IL-1β, IL-2, IL-6, IL-8, TNF-α, and other inflammatory factors mediated by NF-κB (Zhang Y. et al., 2022). Evodiamine can also significantly reduce the pathological damage of breast tissue, inhibit the activation of inflammation-related pathways such as AKT, NF-κB p65, ERK1/2, p38, and JNK, and significantly reduce the production of pro-inflammatory cytokines (Yang Y. et al., 2022). Evodiamine can also improve ulcerative colitis by down-regulating NF-κB signal pathway and NLRP3 inflammatory bodies (Shen et al., 2019). Furthermore, limonin can reduce hepatic steatosis, lipid accumulation, and the expression of p-STAT3/STAT3, caspase-8, and prostaglandin-endoperoxide synthase 2, and improve the inflammatory response of non-alcoholic fatty liver (Wang W. et al., 2023). Limonin can effectively regulate the inflammation mediated by CD4^+^T cells and inhibit the proliferation of CD4^+^T cells by inhibiting the nuclear translocation of NF-κB p65 in activated CD4^+^T cells (Kim W. et al., 2009). Limonin also participates in the regulation of inflammatory pathways by effectively inhibiting p38 MAP. Limonin can also counteract hypertension and vascular damage associated with metabolic syndrome by reducing inflammation and fibrosis (Hassan et al., 2018). Additionally, limonin significantly decreased TNF-α, IL-1β, and IL-6 and inhibited the expression of inflammatory factors in lipopolysaccharide LPS-induced acute lung injury in mice (Wang et al., 2018b).

### 7.5 Analgesia

The analgesic effect of EF is closely related to its anti-inflammatory effect. Oral 50% or 70% methanol extract of 200 mg/kg EF has an analgesic effect on writhing induced by acetic acid (Matsuda et al., 1997). The analgesic effect of EF is related to its metabolites, including evodiamine, rutaecarpine, dehydroevodiamine, rutin, and limonin. Evodiamine exerts analgesic effects through various mechanisms, such as inhibiting ion channels, directly suppressing pain signals, reducing neuronal inflammation, restoring the balance between excitatory and inhibitory neurotransmission, and modulating neurotransmitter release (Jiang et al., 2022). *In vitro*, evodiamine can significantly reduce capsaicin-induced current and thermal hyperalgesia in rats by activating TRPV1 and neuron desensitization (Iwaoka et al., 2016). Evodiamine can also inhibit migraine-like pain response, which may be due to the regulation of nNOS and the inhibition of α-amino-3-hydroxy-5-methyl-4-isoxazolepropionic acid receptor glutamate A1 (Lin et al., 2020). Evodiamine has been shown to inhibit neuropathic pain, improving paclitaxel-induced neuropathic pain by suppressing inflammatory responses and maintaining mitochondrial antioxidant function (Wu and Chen, 2019). In addition, limonin exhibits significant pain-relieving effects at 30 or 100 mg/kg doses. This analgesic effect is likely associated with its anti-inflammatory properties (Matsuda et al., 1998).

### 7.6 Anti-tumor

EF exhibits potent anti-cancer properties and significant healing properties against various cancers, including lung, liver, stomach, breast, and cervical. Studies have shown that 70% ethanol extract of EF can significantly reduce the vitality of human cervical cancer HeLa cells at 20–60 μg/mL and show a certain concentration correlation (Park et al., 2017). Evodiamine has anti-tumor effects by inducing apoptosis, blocking the cell cycle, regulating autophagy, and inhibiting tumor cell metastasis (Luo et al., 2021; Panda et al., 2023). Evodiamine can treat non-small cell lung cancer (NSCLC) by down-regulating the expression of SOX-9 and β-catenin and significantly inhibiting cell migration by inhibiting epithelial-mesenchymal transition (EMT) (Panda M. et al., 2024). Then, evodiamine can induce cell cycle arrest in the G2/M phase, inhibit cell migration, and inhibit the Notch3 signal pathway against NSCLC by inhibiting γ-secretase (Yang X. et al., 2020). Evodiamine can increase the expression of cleaved-caspase-3, decrease the activity of TSGF and alpha-fetoprotein, induce AKT-mediated apoptosis, and exert an anti-hepatoma effect (Yang F. et al., 2017). Moreover, evodiamine inhibits PI3K/AKT, ERK1/2, and p38 MAPKs and promotes apoptosis of ovarian cancer cells by activating caspase-9/8/3 and poly ADP-ribose polymerase cleavage (Wei et al., 2016). Evodiamine activates retinoblastoma protein through p53 and p21, which selectively inhibits breast cancer stem cells in the G1/S phase, resulting in cancer cell death (Han et al., 2016). Evodiamine inhibits the proliferation and induces apoptosis of cholangiocarcinoma cells, inhibits the migration and invasion of cholangiocarcinoma cells, suppresses IL-6/STAT3 signal transduction by upregulating the expression of SHP-2, and treats cholangiocarcinoma (Zhu et al., 2019).

Beyond that, rutaecarpine can inhibit CYP1A1 and exert an anti-tumor effect by inhibiting the binding of 2,3,7,8-Tetrachlorodibenzo-p-dioxin to its receptor (Rannug et al., 1992). Rutaecarpine can also significantly inhibit human CYP1A2 and CYP3A4 (Don et al., 2003; Iwata et al., 2005). Moreover, limonin can inhibit the cell activity of colorectal cancer cells, block STAT3 signal transduction, and inhibit the proliferation, migration, invasion, and colony formation of colorectal cancer cells (Zhang W. F. et al., 2024). Limonin exhibits properties that combat breast cancer. It has been reported that limonin has cytotoxicity on estrogen receptor-positive or negative human breast cancer cells, which may inhibit proliferation by activating caspase-7 dependent pathway (Kim et al., 2013). What’s more, dehydroevodiamine pancreatic is the activator of DNA damage-inducible transcript 3 (DDIT3) and has the ability to inhibit the AKT/mammalian target of rapamycin (mTOR) pathway. It can effectively inhibit the proliferation of pancreatic ductal adenocarcinoma cells, and the growth of prostate cancer stem cells *in vitro* and *in vivo* (Zhu et al., 2024).

### 7.7 Glucose and lipid metabolism regulation

EF can warm the stomach, invigorate the spleen, and be used in glucose and lipid metabolism. In fat metabolism, evodiamine reduced the food intake rate and weight gain rate of rats after growth by downregulating the expression of neuropeptide Y (NPY) and agouti-gene-related protein (AgRP) mRNA and peptide expression in hypothalamic arcuate nucleus (Shi et al., 2009). Evodiamine, a new non-irritant vanillic acid receptor agonist, can simultaneously induce heat loss and heat production, dissipate food energy, and prevent visceral fat accumulation and weight gain (Kobayashi et al., 2001). Then, evodiamine can also activate AMPK and adiponectin polymerization in 3T3-L1 adipocytes, which is related to the activation of Ca^2+^-dependent PI3K/Akt/CaMKII signal pathway (Liu et al., 2014). Treatment with evodiamine over 13 weeks has been shown to lower serum total cholesterol, low and high-density lipoprotein cholesterol, and triglycerides in obese rats on a high-fat diet, decrease blood lipids, and mitigate obesity-related symptoms (Zhang et al., 2017). The combination of berberine and evodiamine can affect the protein expression of PPARγ and liver X receptor α in hyperlipidemic rats and reduce the level of blood cholesterol in hyperlipidemic rats (Zhou et al., 2017b). Furthermore, evodiamine acts as a berberine promoter, and the mechanism of synergistic reduction of serum cholesterol in rats involves inhibiting the expression of Acetyl-CoA Acetyltransferase 2 (ACAT2), Niemann-Pick C1-like 1 (NPC1L1) and apolipoprotein B-48 (apoB-48) to reduce blood lipids (Zhou et al., 2017a). Moreover, ruteacarpine inhibited the expression of NPY and AgRP in the hypothalamic arcuate nucleus and the expression of these two neuropeptides in N29-4 neuronal cells. This method effectively lowers blood cholesterol, non-fasting glucose, insulin, and leptin and ameliorates obesity (Kim S. J. et al., 2009).

On top of that, EF can also contribute to glucose metabolism and improve the symptoms of diabetes. Studies have found that low-dose evodiamine can prevent increased body weight and improve glucose tolerance in mice. Enhanced phosphorylation of AMPK and reduced mTOR signal transduction, a regulator of energy metabolism, are observed in white adipose tissue and are known to avert obesity and insulin resistance (Yamashita et al., 2015). Evodiamide inhibits insulin-stimulated mTOR-S6K activation and IRS1 serine phosphorylation in adipocytes and improves glucose tolerance in obese/diabetes mice (Wang T. et al., 2013). Evodiamine and rutaecarpine can inhibit gluconeogenesis and adipogenesis by activating constitutive androstane receptor (CAR) *in vitro* and *in vivo* and have therapeutic potential for the treatment of hyperglycemia and diabetes mellitus type 2 (Yu et al., 2016). Rutaecarpine can regulate the IRS1/PI3K/AKT signal pathway in the liver and AMPK/acetyl-CoA carboxylase 2 signal pathway in skeletal muscle to improve hyperlipidemia and hyperglycemia in fat-fed and streptozotocin-treated rats (Nie et al., 2016). Beyond that, EF polysaccharides extracted by water solvent have strong antioxidant activity and α-glucosidase inhibition. It is a promising natural antioxidant and α-glucosidase inhibitor.

### 7.8 Protective effect of liver and kidney

EF can warm the liver and kidney, protect the liver and kidney, and is closely related to evodiamine, rutaecarpine, and limonin. In the liver, evodiamine can promote the translocation of Nrf2 into the nucleus, thereby reducing reactive oxygen species (ROS) levels and oxidative stress in grass carp hepatocytes. It also downregulates the MAPK pathway, alleviating DEHP-induced apoptosis and restoring the expression of antioxidant genes. By blocking the Nrf2/MAPK pathway, evodiamine inhibits DEHP-induced apoptosis in grass carp hepatocytes (Lei et al., 2023; Xiong et al., 2022). Evodiamine (15 and 25 mg/kg) has an anti-fibrotic effect on CCl4-induced hepatic fibrosis and reduces the proliferation and collagen metabolism of hepatic stellate cells *in vitro* by down-regulating the relative expressions of TGF-β1, p-Smad2/3, and α-smooth muscle actin (Yang D. et al., 2018). Additionally, rutaecarpine upregulates antioxidant enzymes through CaMKII-Akt and Nrf2/antioxidant response element (ARE) pathways, enhances the expression of HO-1 in hepatocytes, and has a protective effect on hepatotoxicity induced by TBHP (Jin et al., 2017). Rutaecarpine protects mice from acute acetaminophen-induced liver injury by activating antioxidant enzymes. It could significantly reduce the activity of serum ALT/AST and MDA induced by acetaminophen and prevent liver GSH depletion induced by acetaminophen (Choi et al., 2021). Moreover, limonins have a furan ring structure and are easily activated to form RMs, which are crucial in induced hepatotoxicity (Liu Y. et al., 2020). CYP3A4 inducer aggravated the hepatotoxicity induced by large flower germ, while limonin reduced its hepatotoxicity (Zhang et al., 2021c). Limonin can also diminish the liver toxicity caused by acetaminophen by activating the Nrf2 antioxidant signal and suppressing NF-κB inflammation by increasing SIRT1 levels. Limonin shows potential as a treatment for liver damage caused by acetaminophen (Yang R. et al., 2020).

In renal metabolism, I/R injury can lead to acute kidney failure. Owing to its antioxidant, anti-inflammatory, and anti-apoptotic properties, evodiamine can reduce the biochemical and pathological tissue of renal I/R injury in rats (Eraslan et al., 2019). Studies have shown that evodiamine can also protect against LPS-induced acute renal injury and cytotoxicity by regulating ROS NF-κB-mediated inflammation (Shi et al., 2019). What is more, rutaecarpine can prevent and treat renal I/R injury by inhibiting JNK/p38 MAPK signal pathway and interfering with oxidative stress (Wang et al., 2017). Moreover, limonin, acting as a natural ERK2 agonist, plays a role in averting ischemic acute renal damage, primarily through the activation of the ERK signal pathway, which aids in the growth of renal tubular cells and diminishes apoptosis following acute kidney injury (AKI) (Zhou et al., 2023). Limonin regulates arachidonic acid metabolism by inhibiting CYP3A4 activity, thus improving cisplatin-induced acute renal injury and ultimately protecting renal function (Zeng et al., 2023).

### 7.9 Insecticidal and antibacterial

Ancient records indicate that EF possesses insecticidal properties. The extracts and metabolites of EF showed specific insecticidal properties. Findings indicated superior deworming effects of ethyl acetate, petroleum ether, and methanol extract on Goldfish-*Gyrodactylus kobayashii* in living organisms, with EF ethyl acetate extract emerging as the most efficient and secure (Lian et al., 2019). Subsequently, EF volatile oil exhibits insect-killing properties against maize weevils, *Sitophilus zeamais*, and red flour beetle *Tribolium castaneum*, showing LC_50_ values of 36.89, 24.57, and 57.31 mg/L air, in that order (Liu and Du, 2011). In addition, evodiamine and rutaecarpine have insecticidal activity against larvae of *Drosophila melanogaster* with LC_50_ values of 0.30 and 0.28 μmol/mL diet, respectively, among which rutaecarpine has the strongest activity (Miyazawa et al., 2002). Evodiamine and rutaecarpine had strong insecticidal activity against the fourth instar larvae of *Aedes albopictus* with LC_50_ values of 12.51 and 17.02 μg/mL, respectively. EF ethanol extract, limonin, and evodiol also had insecticidal activity against Asian tiger mosquitoes, and the LC_50_ values were 43.21, 32.43, and 52.22 μg/mL, respectively (Liu et al., 2012). Moreover, the nematicidal activity of evodiamine and rutaecarpine against *Meloidogyne incognita* was stronger than the crude EF ethanol extract, and the LC_50_ values were 73.55, 120.85, and 131.54 μg/mL, respectively. Both evodiol and limonin demonstrated their ability to kill *Meloidogyne incognita*, evidenced by LC_50_ values of 155.02 and 197.37 μg/mL, respectively, yet they were less potent than the raw EF ethanol extract (Liu et al., 2013). Other studies found that limonin had effective biological activity against larvae and adults of *Schistosoma mansoni*, and its antiparasite activity was enhanced in a dose-dependent manner (Eraky et al., 2016).

EF’s long-term use in treating diarrhea and beriberi is due to its antibacterial and antifungal properties. The EF volatile oil has the strongest activity against *Bacillus subtilis* and *Staphylococcus aureus*, the maximum inhibitory zone diameter is 17.9 and 12.2 mm, respectively, and the MIC is 3.2–6.4 mg/mL (Liu S. S. et al., 2019). Furthermore, three novel quinazoline alkaloids, specifically evodiamine A, evodiamine B, and evodiamine C, were extracted from EF methanol. They prohibited excellent inhibition against *Xanthomonas oryzae* pv. o*ryzae*, *Xanthomonas oryzae* pv. o*ryzicola*, and *Xanthomonas campestris* pv. c*ampestris*, with respective EC_50_ values of 3.13, 14.32, and 32.72 nmol (Su et al., 2018). Moreover, evodiamine can enhance the activation of NLRP-3 inflammatory bodies by inducing acetylation of α-tubulin lysine 40 residues, thus enhancing innate immunity to bacterial infection (Li C. G. et al., 2019).

### 7.10 Anti-osteoporosis

Rutaecarpine, evodiamine, and limonin demonstrated obvious anti-osteoporotic effects. Rutaecarpine significantly inhibits osteoclast production and bone resorption of bone marrow-derived macrophages osteoclasts by reducing the protein level of nuclear factor of activated T cells 1 (NFATc-1) and phosphorylation of other signal pathways during osteoclast differentiation (Fukuma et al., 2018). In addition, evodiamine has been reported to inhibit osteoclast formation by blocking receptor activators for NF-κB ligand (RANKL)-induced ERK and c-Fos activation and NFATc-1 induction in a dose and time-dependent manner (Jiang et al., 2017). Evodiamine can inhibit osteoclast formation induced by RANKL through NF-κB and calcium signaling pathways and reduce bone loss in ovariectomy and ovariectomized mice by inhibiting osteoclast production (Jin et al., 2019). Subsequently, evodiamine is capable of mitigating osteoporosis in zebrafish caused by dexamethasone, by counteracting the disproportion in bone development and resorption and triggering the matrix metalloproteinase 3-osteopontin-MAPK pathway signal (Yin et al., 2019). Beyond that, limonin can increase the calcium concentration of the femur and fifth lumbar vertebra in ovariectomized rats, and the mechanism may be related to promoting bone formation (Mandadi et al., 2009). Research has found that the loss of ovarian function can lead to a lack of ovarian-related hormones, resulting in rapid loss of ovarian-related bones (Renno et al., 2006). Limonin can efficiently prevent bone mass reduction and enhance bone mineral density in rats post-ovariectomy. Moreover, limonin stimulates the activity of ALP in osteoblast MC3T3-E1 and enhances the expression of osteoblast differentiation gene markers by regulating extracellular signal-regulated kinase and P38 signal (Lee et al., 2016).

### 7.11 Other activity

EF not only offers attractive pharmacological effects but also provides antiallergic, antioxidant, antidepressant, and protection for the prostate, among others.

#### 7.11.1 Antiallergic

*In vitro* and *in vivo*, evodiamine and rutaecarpine may inhibit the biosynthesis of allergy-related cytokines (TNF-α and IL-4) in mast cells and basophils, suggesting that they may be effective against IgE-induced allergic diseases such as atopic dermatitis and rhinitis (Shin et al., 2007). Subsequently, limonin effectively manages allergies induced by IgE. This substance can significantly reduce IgE production in the peripheral blood mononuclear cells (PBMC) and B cell lines of children allergic to food, potentially owing to the suppression of ε-germ line transcript expression in PBMC (Yang et al., 2014).

#### 7.11.2 Antioxidant

In natural aging rats, limonin decreased the levels of MDA and lipofuscin in serum and brain tissue, increased the activities of superoxide dismutase (SOD) and GSH-Px in serum and brain tissue, and enhanced the total antioxidant capacity in brain tissue (Li et al., 2016). Notably, when altered by the structure of limonin, limonin glycosides can attain antioxidant properties by neutralizing free radicals (Poulose et al., 2005). However, some researchers question whether limonin has antioxidant activity (Breksa and Manners, 2006). There is a lack of research on the antioxidant mechanism of limonin, and this natural antioxidant is worthy of further exploration.

#### 7.11.3 Antidepressant

The antidepressant effect of evodiamine on chronic, unpredictable stress rats may be achieved by regulating monoamine transmitters and BDNF-TrkB signal transduction in the hippocampus (Jiang et al., 2015).

#### 7.11.4 Prostate protection

The EF ethanol extract has a strong 5α-reductase inhibitory activity. The treatment of EF ethanol extract in benign prostatic hyperplasia-1 cells inhibits cell viability through caspase-8 and cystatin-3-dependent apoptosis and effectively inhibits the growth of benign prostatic hyperplasia-1 cells (Park et al., 2018). In addition, EF volatile oil has obvious anti-inflammatory effects and inhibits the growth of prostate cancer-3 cells by directly and indirectly regulating the cytokine secretion profile of spleen cells (Yeh and Lin, 2021). Not only this, evodiamine can also inhibit prostate hyperplasia and migration through the PI3K/AKT/NFκB signal pathway, indicating that it may be a potential lead drug in the treatment of prostate cancer (Lei et al., 2022).

## 8 Toxicity

Ancient TCM texts have chronicled EF’s mild toxic effects, potentially causing eye harm, hair fall, and gastrointestinal issues if misused. In recent years, many studies have shown that overuse of EF can cause toxic symptoms such as nausea, vomiting, abdominal pain, diarrhea, and blurred vision (Ma et al., 2018) and cause liver and kidney toxicity to the human body (Teschke, 2014). EF and its metabolites have been reported to cause liver damage at high doses and induce arrhythmias, leading to cardiotoxicity (Teschke et al., 2014). *In vitro* and *in vivo* studies have shown that EF has hepatotoxicity, cardiotoxicity, and nephrotoxicity. However, there are few studies on the cardiotoxicity and nephrotoxicity of EF. The toxicity of EF is summarized as follows (Table 5).

**TABLE 5:** Toxicity of EF and its metabolites.

Toxicology	Toxicity mechanisms	Chemical compounds	Type of study	Experimental subject	Dose range	Duration	Toxic manifestations	References
Hepatotoxicity	Oxidative damage and inflammatory response	EF aqueous extract	*In vivo*	KM mice	10, 20, 30 g/kg	21 days	(−): The level of SOD/MDA, GSH-Px and NOS. (+): The level of ALT, AST, GSH, MDA, IL-1β, IL-6 and TNF-α	Zhou et al. (2013a)
	Oxidative damage and inflammatory response	EF aqueous extract	*In vivo*	Male SD rats	6, 12, 24 g/kg	15 days	(−): The level of MnSOD, GSH, ATP, and mitochondrial potential (+): The level of MDA.	Cai et al. (2014)
	Oxidative damage	EF essential oil	*In vivo*	KM mice	0.25, 1, 1.25 mL/kg	7 days	(−): The level of SOD, GSH, and GSH-Px (+): The level of MDA, NO, and NOS.	Zhang et al. (2011)
	Oxidative damage	Evodiamine and rutaecarpine	*In vitro*	HLM and P450s	10 and 50 mmol/L, respectively	60 min	(−): The level of GSH; the activation of CYP3A4	Wen et al. (2014)
	Inflammatory response	EF aqueous extract	*In vivo*	KM mice	0.65, 3.25, 6.5 g/kg	15 days	(−): Liver mitochondrial (Ca^2+^)-ATP activity; the expression of SOD and Bcl-2 protein (+): The level of ALT, AST, MDA, TNF-α, and IL-1β; the expression of Bax protein	Liu et al. (2018)
	Inflammatory response	EF aqueous and 70% ethanol extract	*In vivo*	KM mice	0.01 mL/g	15 days	(−): The expression of STAT3 and Src proteins (+): The expression of ERK, cyclin-dependent kinase 8, and casein kinase 1 ε proteins	Liao et al. (2014)
	Inflammatory response	EF aqueous extract	*In vivo*	Male C57BL/6 mice	7.5 g/kg	1 h	(−): The level of SOD and GSH-PX. (+): The level of ALT, AST, ALP, LDH, MPO, MDA, TNF-α, IL-6 and IL-1	Ren et al. (2024)
	Mitochondrial damage	EF aqueous and 70% ethanol extract	*In vivo*	KM mice	0.2 mL/10 g	14 days	(−): Mitochondria function and the ratio of AST/ALT.	Yang et al. (2024b)
	Mitochondrial damage	Evodiamine	*In vitro*	L-02 cells	6.25–200 μmol/L	14 days	(−): The cell counts and MMP.	Yang et al. (2024b)
	Mitochondrial damage	Evodiamine	*In vitro*	HepG2 cells	0.2, 1, 5 μmol/L	48 h	(−): The SOD activity and MMP. (+): The ALT, AST, LDH, ALP activities; the content of total bilirubin and MDA; cells apoptosis	Gao et al. (2021)
	Formation of drug-protein adducts	Rutaecarpine	*In vitro*	Primary male SD rat hepatocyte	10, 30, 100, 300 mmol/L	24 h	(−): Hepatocyte survival; mitochondrial membrane potential; the acitivity of CYPs (+): The level of ROS and LDH; cellular stress and membrane damage	Cai et al. (2014)
	Formation of drug-protein adducts	Rutaecarpine	*In vivo*	KM mice	10, 20, 30 mg/kg	7 days	(+): The expression of hepatic transporters, CYP, and phase-2 enzyme genes	Labbe et al. (2008)
	Formation of drug-protein adducts	Evodiamine	*In vivo*	Male SD rats	50 mg/kg	7 days	(−): The expression of CYP1A2, CYP2C9 and CYP2D6	Zhang et al. (2016)
Cardiotoxicity	Oxidative damage	Evodiamine	*In vitro*	SD Rat cardiomyocytes	31.3, 62.5, 125, 250 μg/mL	24 h	(−): Cardiomyocyte viability; heart rate; the level of SOD. (+): The level of MDA and LDH.	Yang et al. (2017b)
	Oxidative damage	Evodiamine	*In vivo*	Wild-type zebrafish and the transgenic strain zebrafish	31.3, 62.5, 125, 250 μg/mL	24 h	(+): The straight-line distance between the venous sinus and arterial bulb	Yang et al. (2017b)
	Oxidative damage	Evodiamine and rutaecarpine	*In vitro*	H9C2 and NRCMs	5, 10, 25, and 60, 80, 100 μmol/L, respectively	24 h	(+): The levels of LDH and CK; MMP.	Zhang et al. (2022a)
	Oxidative damage	EF aqueous extract	*In vivo*	Male SD rats	0.525 g/mL	15 days	(−): The protein expression of the cyclic guanosine monophosphate-protein kinase G pathway; frequency of spontaneous beat in NRCMs (+): The intensity of calcium fluorescence	Zhang et al. (2022a)
	Oxidative damage	EF aqueous extract	*In vivo*	Wild-type zebrafish and the transgenic strain zebrafish	0.4 mg/mL	48 h	(+): The morphological abnormalities in the liver, myocardial concentrations, and pericardial edema	Fan et al. (2024)
	Inhibition of the cardiac hERG channel	Hydroxyrutaecarpine	*In vitro*	HEK 293 cells	10 μmol/L	24 h	(−): The expression of transcription factor Sp1 and hERG protein; the activation of hERG channel	Li et al. (2022c)
	Inhibition of the cardiac hERG channel	Rutaecarpine	*In vitro*	HEK 293 cells	1, 10 μmol/L	24 h	(−): The expression of transcription factor Sp1 and hERG protein	Zhan et al. (2021)
	Inhibition of the cardiac hERG channel	Rutaecarpine	*In vivo*	Male guinea pigs	25 mg/kg/d	2 weeks	(+): The QT/QTc intervals; the induction rate of ventricular fibrillation	Zhan et al. (2021)
	Inhibition of the cardiac hERG channel	Dehydroevodiamine and hortiamine	*In vitro*	HEK 293 cells	0.01, 0.1, 1, 10 μmol/L	−	(+): The action potential duration and early afterdepolarizations	Baburin et al. (2018)
	Inhibition of the cardiac hERG channel	Dehydroevodiamine and hortiamine	*In vivo*	Anesthetized rabbits and CAVB dogs	0.05, 0.5, and 0.33 mg/kg, respectively	5 min	(+): The QT interval	Baburin et al. (2018)
Nephrotoxicity	Renal apoptosis	EF 70% ethanol extract	*In vivo*	SD rats	2.5, 6.6, 20.83 g/kg/d	28 days	(+): Glomerular mesangial curvature, swelling of renal podocytes and glomerular vascular endothelial cells	Liu et al. (2015)
	Renal apoptosis	Evodiamine	*In vitro*	HK-2 cells	0.1, 0.2, 0.4, 0.8, 1.6, 3.2 μmol/L	48 h	(+): The calcium overload caused by activation of the TRPV1 protein; PI3K pathway-mediated apoptosis	Yang et al. (2024a)
	Renal apoptosis	Evodiamine	*In vitro*	HK-2 cells	0.1, 0.2, 0.4, 0.8, 1.6, 3.2 μmol/L	24 h	(−): The mitochondrial membrane potential (+): The cell membrane permeability and Cytochrome C release; the level of LDH; the expression of apoptosis-related proteins Bax and Bcl-2	Yang et al. (2022a)
	Renal apoptosis	Evodiamine and evodine	*In vitro*	HEK 293 cells	4.15, 8.3, 16.6, 33.2, and 25, 50, 100, 200 μg/mL, respectively	24 h	(+): Varying degrees of shrinkage, reduction, and even death of renal cells	Zhou et al. (2013b)

### 8.1 Hepatotoxicity

EF causes hepatocyte cytotoxicity, attributed to oxidative stress, mitochondrial damage, endoplasmic reticulum stress, liver metabolic disorder, and apoptosis (Cai et al., 2014). The toxicological mechanisms are peroxidation, inflammatory factors, mitochondrial damage, and the formation of drug-protein adducts. Research revealed that various EF extracts might lead to sudden liver damage, with volatile oil exhibiting the highest level of hepatotoxicity, succeeded by total extract and ethanol extract, and water extract showing minimal hepatotoxicity.

#### 8.1.1 Oxidative damage

EF may cause oxidative harm by impacting vital elements of the body’s oxidation-antioxidant mechanism. Studies have shown that EF aqueous extraction can cause liver injury in mice after continuous intragastric administration for 21 days. It was found that the content of MDA in liver tissue increased, the ratio of SOD/MDA and the activity of GSH-Px decreased significantly, and the pathomorphology showed focal hepatocyte necrosis (Zhou L. et al., 2013). The hepatotoxicity of EF is related to the oxidative stress in the liver and has a certain dose-effect relationship. Following 15 days of orally administering EF via aqueous extraction, there was a notable reduction in SOD activity in the mice’s liver tissues, with SOD levels rising in each dosage group as the dose was increased (Cai et al., 2014). In addition, liver injury occurred after continuous intragastric administration of EF volatile oil for 7 days, resulting in increased activities of MDA and NOS in blood and liver tissue, decreased GSH content, SOD, and GSH-Px activities (Zhang et al., 2011). Research indicates that the presence of 3-alkylindoles in evodiamine and rutaecarpine leads to the creation of highly electrophilic intermediates, namely iminoquinone and 3-methyleneindolenine, via P450-driven oxidation in liver microsomes (primarily driven by CYP3A4 and to a smaller degree by CYP1A2 and CYP2D6), causing harmful effects on hepatocytes when GSH is depleted (Wen et al., 2014).

#### 8.1.2 Inflammatory response

Inflammatory injury is one of the causes of liver injury caused by EF. IL-1β, IL-6, and TNF-α are inflammatory transmitters closely related to inflammatory response. These inflammatory transmitters can further amplify the signal of inflammatory response and promote apoptosis and necrosis of hepatocytes. Findings indicate that mice experienced liver damage and elevated levels of TNF-α and IL-1β in their liver tissue 15 days following the EF aqueous extraction (Liu et al., 2018). After continuous intragastric administration of EF aqueous extraction for 21 days, the high, middle, and low dose groups of EF could significantly increase the contents of TNF-α, IL-1 β, and IL-6 in liver tissue of mice, with a certain dose-effect relationship (Zhou L. et al., 2013). After continuous intragastric administration of EF aqueous and ethanol extraction for 15 days, the expression of phosphorylated ERK1/2 in the liver of mice was significantly upregulated. Activation of ERK1/2 can induce cells to produce TNF-α, which mediates inflammatory response and apoptosis-related transcriptional regulatory factors (Liao et al., 2014). During metabolic processes, the P450 enzyme activates evodiamine in EF aqueous extraction, resulting in liver damage and inflammation, primarily due to elevated levels of ALT, AST, ALP, LDH, MPO, MDA, TNF-α, IL-6, and IL-1 (Ren et al., 2024).

#### 8.1.3 Mitochondrial damage

Mitochondria are the main sites of cell biological oxidation, which mainly synthesize ATP. Mitochondria are essential targets of drug toxicity in drug-induced liver injury. Studies have shown that intragastric administration of EF aqueous extracts of 6, 12, and 24 g/kg for 15 days can cause hepatocyte mitochondrial swelling and vacuolation, and eventually lead to apoptosis due to ATP depletion and cytochrome C release (Cai et al., 2014). Moreover, both ethanol and aqueous extracts of EF have hepatotoxicity, and the cytotoxicity of EF ethanol extract is stronger. In addition, evodiamine has the strongest toxicity among the extracts of EF, which can significantly reduce the number of cells and increase the mitochondrial membrane potential (MMP) *in vitro* (Yang et al., 2024b). Evodiamine (0.04–25 μmol/L) decreased the survival rate of HepG2 cells, increased MMP, and induced apoptosis in a time- and dose-dependent manner (Gao et al., 2021). Mitochondrial permeability transition plays an important role in mediating hepatocyte injury (Labbe et al., 2008; Pessayre et al., 2010). Limonin has hepatotoxicity, which can cause oxidative damage to rat mitochondria, lead to mitochondrial swelling, mitochondrial permeability transition pore opening, mitochondrial potential decrease, and finally trigger cell death signal pathway (Fan et al., 2019).

#### 8.1.4 Formation of drug-protein adducts

The alkaloids easily combine with proteins to form drug-protein adducts. Drug-protein adducts may cause toxicity by damaging the physiological function of the modified protein or through an immune-mediated mechanism (Zhou et al., 2005). The role of RMs is significant in liver damage caused by drugs. The secondary amine configuration of Rutaecarpine enables its activation into RMs via the CYPs enzyme, leading to a covalent bond with CYPs and proteins in rat liver cells, resulting in drug-protein complexes and subsequent liver damage (Zhang et al., 2015). RMs can consume GSH, leading to excessive ROS production, respiratory chain dysfunction, cell stress, mitochondrial damage, cell membrane damage, and hepatocyte damage (Akbulut et al., 2014). CYPs are the primary enzymes involved in drug metabolism within the human body. Certain medications transform into RMs via the biological actions of CYPs (Yan et al., 2023). It has been found that rutaecarpine can inhibit many types of CYP activity, such as CYP1A2, CYP2C9, CYP2C19, and CYP2E1 (Zhang et al., 2015). The induction of cytochrome P450 enzyme gene, liver transport protein, and phase 2 enzyme gene are involved in the interaction between evodiamine and drugs (Zhu et al., 2013). Evodiamine and rutaecarpine can cause toxicity through P450-mediated dehydrogenation, produce highly electrophilic intermediates, and lead to drug-drug interaction mainly through the inactivation of CYP3A4 (Wen et al., 2014). In addition, evodiamine can inhibit CYP1A2, CYP2C9, and CYP2D6 in rats (Zhang et al., 2016). Evodiamine is easily oxidized to an epoxy structure that binds to GSH. When GSH is depleted, some liver damage will occur (Zhang et al., 2015).

### 8.2 Cardiotoxicity

The cardiotoxicity of EF is mainly caused by oxidative damage and inhibition of human ether-a-go-go-related gene (hERG) channels in the heart. Its primary connections are in alkaloids with evodiamine, rutaecarpine, dehydroevodiamine, and hydroxyrutaecarpine.

#### 8.2.1 Oxidative damage

The heart is the most oxygen-consuming organ, and many basic studies have confirmed the cardiotoxicity mediated by oxidative stress (Shen et al., 2024). Oxidative damage is closely related to evodiamine and rutaecarpine. Studies have shown that evodiamine at the concentration of 31.3–250 μg/mL for 24 h can significantly reduce the level of MDA and the activity of superoxide dismutase, resulting in oxidative stress injury of cardiomyocytes. Subsequently, evodiamine could induce oxidative stress by generating free radicals, potentially harming the architecture and functionality of cardiomyocytes. After being treated with 28.44 μg/mL evodiamine for 24 h, cardiomyocytes reached 50% inhibitory concentration, which significantly decreased the activity of SOD in rat cardiomyocytes. In the zebrafish model, the mortality rate of zebrafish treated with 354 ng/mL evodiamine was 10%, causing cardiac dysfunction and pericardial malformations (Yang W. et al., 2017). The findings imply that evodiamine could lead to heart-related side effects, including oxidative stress. Determining LDH and creatine kinase (CK) activity is one of the biochemical indexes for evaluating and diagnosing heart disease. The level of LDH in serum reflects the injury of myocardial cell permeability. The activity level of CK is directly related to the consumption and supply of myocardial oxygen and energy, muscle contraction, and mitochondrial function (Zervou et al., 2016; Bak and Schousboe, 2017; Klein et al., 2020). Evodiamine and rutaecarpine have toxic effects on rat cardiomyocytes H9c2 and neonatal rat cardiomyocytes (NRCMs), mainly by reducing the protein expression of cyclic guanosine monophosphate-protein kinase G pathway in H9c2 cells and changing the spontaneous beating frequency in NRCMs (Zhang D. et al., 2022). What is more, a high dose of evodiamine will lead to severe morphological abnormalities of the liver, pericardial edema, and increased myocardial concentration.

#### 8.2.2 Inhibition of cardiac hERG channel

The hERG channel is the ion channel on the myocardial cell membrane, which is very important to maintain the normal electrophysiological activity of the heart. Abnormal opening or closing of the hERG channel will lead to arrhythmia (Liao et al., 2024). *In vitro*, rutaecarpine can reduce the threonine/tyrosine phosphorylation of Sp1 and the expression of the hERG channel through the PI3K/AKT pathway in HEK 293 cells. Subsequently, administering rutaecarpine for 2 weeks may extend the QT/QTc interval intervals and enhance the rate of ventricular fibrillation induction in the hearts of guinea pigs (Zhan et al., 2021). In addition, dehydroevodiamine can inhibit the hERG channel, change the myocardial excitation process, and lead to arrhythmia and even ventricular fibrillation in severe cases (Zhang et al., 2023). Depending on the dose, dehydroevodiamine, and hortiamine can prolong action potential duration and early afterdepolarizations of cardiomyocytes, eventually leading to arrhythmias (Baburin et al., 2018). There are also studies indicating that hydroxyrutaecarpine inhibits hERG current by binding to F656 and Y652 sites in the hERG channel. It can shorten the inactivation time constant, accelerate the process of channel inactivation, and inhibit the function of the hERG channel (Li X. H. et al., 2022).

### 8.3 Nephrotoxicity

EF’s nephrotoxic effects primarily stem from renal cell death and oxidative stress. Its similarity to evodiamine and limonin in EF is notable. Mice were administered the EF ethanol extract in groups of low, medium, and high dosages. In the group receiving a high dosage, there was a noticeable flexing of the glomerular Mesangium and an enlargement of both glomerular podocytes and endothelial cells (Liu et al., 2015). *In vivo* experiments showed that evodiamine could induce renal cell death and regulate the PI3K/AKT/mTOR pathway by inducing intracellular calcium overload (Yang et al., 2024a). Rutaecarpine can significantly reduce the level of cortisol and regulate glucocorticoid metabolism. Renal injury may be related to the induction of apoptosis-related protein Bax and Bcl2 expression (Yang C. Q. et al., 2022). Evodiamine and evodiolide in alkaloids can damage mitochondria, lead to mitochondrial dysfunction, produce a large number of free radicals from mitochondria, further aggravate oxidative stress, induce apoptosis, and promote renal damage (Zhou Q.j. et al., 2013). In addition, limonin also has a certain toxicity to kidney cells. Comprehensive analysis of animal experiments and chromatographic analysis showed that hydroxyl or acetoxy limonoid derivatives and coumarin in EF may be the leading causes of toxicity (Shan et al., 2021). Studies have shown that limonin (50–200 μg/mL) can significantly inhibit the viability of HEK 293 cells in a dose-dependent manner. A concentration ranging from 100–200 μg/mL may lead to atrophy, reduction, or even fatality of renal cells (Fan et al., 2019).

## 9 Monitoring and prevention of toxicity of EF

### 9.1 Surveillance and prevention of hepatotoxicity

The typical oral dose of EF is 2–5 g, taken by water decoction or pill powder. It is generally non-toxic within a reasonable dose range, but it needs to be monitored if it is overused. The liver function test, coagulation function test, serum bilirubin level test, liver ultrasound, and liver biopsy can monitor the hepatotoxicity of EF. Liver function tests can detect transaminase, bilirubin, alkaline thrombin, cholinesterase, and other indexes in blood and evaluate the functional status of the liver (Shiihara et al., 2024). Coagulation function tests can determine prothrombin time, thrombin time, and activated thrombin time and help to judge whether the liver is abnormal (Kumagai et al., 2023). A high serum bilirubin level may indicate liver injury or biliary obstruction disease (Poynard et al., 2023). Liver ultrasound can observe liver structural abnormalities or steatosis (Vardar et al., 2022). Liver biopsies help to confirm whether there is hepatocyte injury or inflammation (Palmer et al., 2024). Therefore, patients using EF should regularly check their liver function and blood coagulation function, pay close attention to the indexes of transaminase, bilirubin, alkaline thrombin, and cholinesterase, and adjust the dosage of EF in time to avoid drug-induced liver injury.

### 9.2 Surveillance and prevention of cardiotoxicity

The cardiotoxicity of EF can be evaluated by physical monitoring and measurement of specific biomarkers, including electrocardiogram changes, blood electrolyte levels, and myocardial enzyme activity (Qiu et al., 2023). An electrocardiogram is a direct indicator of the heart’s electrophysiological function, and irregular waveforms could signal the presence of an arrhythmia (Henriksen et al., 2023). Disproportionate levels of electrolytes in the blood, particularly irregular potassium ion concentrations, frequently lead to arrhythmias (Sauer and Porter, 2021). Increased myocardial enzyme activity, such as creatine kinase and lactate dehydrogenase, may damage heart tissue (Gai et al., 2021). Therefore, patients using EF should have regular ambulatory electrocardiogram monitoring. The global cardiac activity was measured for 24 h, and the heart rate variability and myocardial perfusion indexes were observed. It assists patients in adjusting their medication dosage promptly to reduce the occurrence of cardiotoxicity.

### 9.3 Surveillance and prevention of nephrotoxicity

The nephrotoxicity of EF can be monitored by urinalysis, renal function tests, biochemical assays, and renal biopsy (Canki et al., 2024). Urine tests can reflect proteinuria, hematuria, hemoglobinuria, cylindruria, urinary calcium, and alkaline urine (Wu and Fenton, 2018). Renal function tests assess the endogenous creatinine clearance rate, blood urea nitrogen, creatinine, and uric acid levels and evaluate the glomerular filtration rate through a radionuclide renogram (Nieto et al., 2020). Biochemical tests can monitor serum liver and renal function-related enzyme levels (Sumer et al., 2020). A renal biopsy is capable of directly monitoring the pathological alterations in kidney tissue, including the deterioration of tubular epithelial cells, necrosis, congestion in the interstitial space, swelling, infiltration of inflammatory cells, fibrosis in the renal interstitial area, and the expansion or shrinkage of the renal tubules (Xu et al., 2024). Therefore, patients taking EF should have regular urinalysis and blood tests and pay attention to the levels of urinary protein, red blood cells, serum creatinine, and urea nitrogen to ensure timely detection of issues and implementation of appropriate treatment measures, thereby reducing the incidence of renal toxicity. Concurrently, it is crucial to rigorously regulate the dosage while administering EF to prevent excessive use. Regular monitoring of kidney function during usage is crucial for early identification of kidney toxicity.

## 10 Conclusion and prospect

EF ranks among the most prevalent and extensively utilized TCM, with a history of use in China spanning millennia. The latest research achievements of EF in standardized cultivation, traditional application, processing methods, quality control, phytochemistry, pharmacology, and toxicology were reviewed in this paper. EF has cardiovascular protection, anti-inflammation, analgesia, gastrointestinal protection, anti-tumor, neuroprotection, glucose and lipid metabolism regulation, and other pharmacological effects. Frequently, it serves as a treatment for abdominal discomfort, vomiting, diarrhea, dyspepsia, high blood pressure, eczema, and oral ulcers. Despite EF’s diverse pharmacological effects, ancient texts document its mild toxicity. The harmful effects of TCM have persistently been a worry in both its clinical usage and formulation. Therefore, it is suggested that the following areas should be considered in future research.

From a botanical perspective, it is evident that ER, ERO, and ERB are the trio of EF sources, with nearly mature fruits primarily categorized into LEF, MEF, and SEF. MEF produced in Jiangxi is a genuine medicinal material mainly distributed in Sichuan, Guizhou, and other places south of the Qinling Mountains in China. However, most current studies only distinguish the varieties of *Euodia rutaecarpa*, and few studies have demonstrated the effect of EF size on pharmacological action. From a conventional standpoint, EF’s initial documentation appears in *Shen Nong’s Herbal Classic*, known for its customary properties of alleviating cold and pain, diminishing nausea and vomiting, enhancing yang, and halting diarrhea. Currently, numerous clinical prescriptions and formulations have been developed, primarily using EF, for treating conditions like abdominal pain, diarrhea, chronic non-atrophic gastritis, irritable bowel syndrome, primary dysmenorrhea, and more. However, the specific mechanism is not completely clear, and the clinical application of EF needs more extensive verification. Moreover, modern pharmacological studies mainly focus on EF or its metabolites, seriously ignoring the effects caused by drug interaction. According to the traditional application, exploring the synergistic effect of drugs to improve clinical efficacy and safety is of great significance. In terms of processing methods, ancient classical works have recorded the processing of EF with licorice, salt, ginger, vinegar, and other methods, which can increase efficiency and reduce toxicity. Modern research has proved that the best result can be obtained when the ratio of EF to licorice is 100: 6. However, few articles currently study this aspect. The metabolites of EF were widely studied. About 300 metabolites were isolated and identified from the plant, including alkaloids, terpenoids, flavonoids, volatile oils, and others. Indole alkaloids, especially evodiamine, and rutaecarpine, have been studied for many years. However, studying terpenoids, flavonoids, and volatile oils in EF is not deep enough, which seriously limits people’s understanding of the pharmacological and toxicological mechanisms. Therefore, further study of other metabolites is a priority for the future. The quality assurance of EF is closely related to evodiamine, rutaecarpine, and limonin. It is stipulated that the content of evodiamine and rutaecarpine is not less than 0.15%, and the content of limonin is not less than 0.20%. However, there is no maximum limit for using these three ingredients. According to modern research, the content of rutaecarpine and limonin should not exceed 100 mg/kg/d, and the content of evodiamine should not exceed 300 mg/kg/d. Although researchers have done a lot of research on the minimum and maximum dose, a unified standard has not been formed, so it is necessary to conduct an in-depth study.

The research on toxicology and its mechanisms of EF *in vivo* and *in vitro* is insufficient, limiting its more extensive application. Contemporary studies extensively focus on EF’s hepatotoxic effects, with lesser emphasis on cardiotoxicity and nephrotoxicity and a lack of comprehensive and clear understanding of the toxicity of EF. After analyzing the metabolites of EF, it is concluded that evodiamine, rutaecarpine, limonin, and dehydroevodiamine are related to cardiotoxicity and nephrotoxicity. In a specific range, the toxicity of EF to the heart and kidney is proportional to the dose, and long-term overuse will aggravate the toxicity. Attention must be paid to the dosage of EF in clinical application. The research on drug dosage is also very scarce, and the dosage of 2–5 g stipulated in ChP is not exactly the same as the actual clinical dose. Therefore, the research on the dose of EF should be further strengthened. *In vitro* and vivo experiments showed that EF may be toxic to the liver through peroxidation injury, inflammatory response factor mediation, mitochondrial damage, and the formation of drug-protein adducts. EF may cause cardiotoxicity through oxidative damage and inhibition of hERG channels in the heart. EF could react to kidney toxicity by inducing apoptosis and oxidative stress. However, EF’s specific toxic risks and potential disadvantages must be further studied, especially cardiotoxicity and nephrotoxicity. The existing studies mainly focus on the preliminary stage of animal experiments and clinical trials. The specific application effect of EF in different diseases needs to be further verified, and its safety needs to be evaluated more comprehensively. Therefore, the clinical use of EF should be closely monitored to ensure its safety.

TCM stands as a repository of abundant resources and distinctive processing techniques. However, the phytochemistry, pharmacology, and toxicology of TCM are complex. Therefore, we should further strengthen the research on the pharmacological and toxicological mechanisms of EF, further expand and optimize its application in medicine, and better meet the clinical needs. EF is a valuable plant medicine with various uses, pharmacological activities, and reliable clinical efficacy. It has significant medicinal value and potential to develop new therapeutic drugs. Therefore, systematic and comprehensive research on its metabolism and pharmacological and toxicological mechanisms is a hot spot.

## Glossary

ACAT2: Acetyl-CoA Acetyltransferase 2
AD: Alzheimer’s disease
AgRP: Agouti-gene related protein
AKI: Acute kidney injury
apoB-48: Apolipoprotein B-48
ARE: Antioxidant response element
Aβ40: amyloid-β 40
CaMKII: Ca2+/calmodulin-dependent protein kinase kinase II
CAR: Constitutive androstane receptor
CCK: Cholecystokinin
CGRP: Calcitonin gene related peptide
CK: Creatine kinase
CP: Chinese Pharmacopoeia
CYP: Cytochrome P450
DDIT3: DNA damage inducible transcript 3
DSS: Dextran Sulfate Sodium Salt
EF: Euodiae Fructus
EMT: Epithelial-mesenchymal transition
eNOS: Endothelial nitric oxide synthase
ER: *Euodia rutaecarpa* (Juss.) Benth.
ERB: ER var. *bodinieri* (Dode) Huang
ERO: ER var. *officinalis* (Dode) Huang
hERG: Human ether-a-go-go related gene
I/R: Ischemia/reperfusion
LEF: Medium flowers of EF
LPS: Lipopolysaccharide
MAP: Mitogen-activated protein
MEF: Large flowers of EF
MMP: Mitochondrial membrane potential
MPO: Myeloperoxidase
MPT: Mitochondrial permeability transition
mTOR: Mammalian target of rapamycin
NFATc-1: Nuclear factor of activated T cells 1
Notch3: Notch receptor 3
NPC1L1: Niemann-Pick C1-like 1
NPY: Neuropeptide Y
NRCMs: Neonatal rat cardiomyocytes
NSCLC: Non-small cell lung cancer
PBMC: Peripheral blood mononuclear cells
RANKL: Receptor activator for nuclear factor-κB ligand
RMs: Reactive metabolites
ROS: Reactive oxygen species
SEF: Small flowers of EF
SOD: Superoxide dismutase
TCM: Traditional Chinese Medicine
UC: Ulcerative colitis
