# Supplementary figures and images for: Euodiae Fructus: a review of botany, application, processing, phytochemistry, quality control, pharmacology, and toxicology
Source: Front Pharmacol. 2025 Jan 29;16:1509032. doi: 10.3389/fphar.2025.1509032 (PMC11813794; doi:10.3389/fphar.2025.1509032)

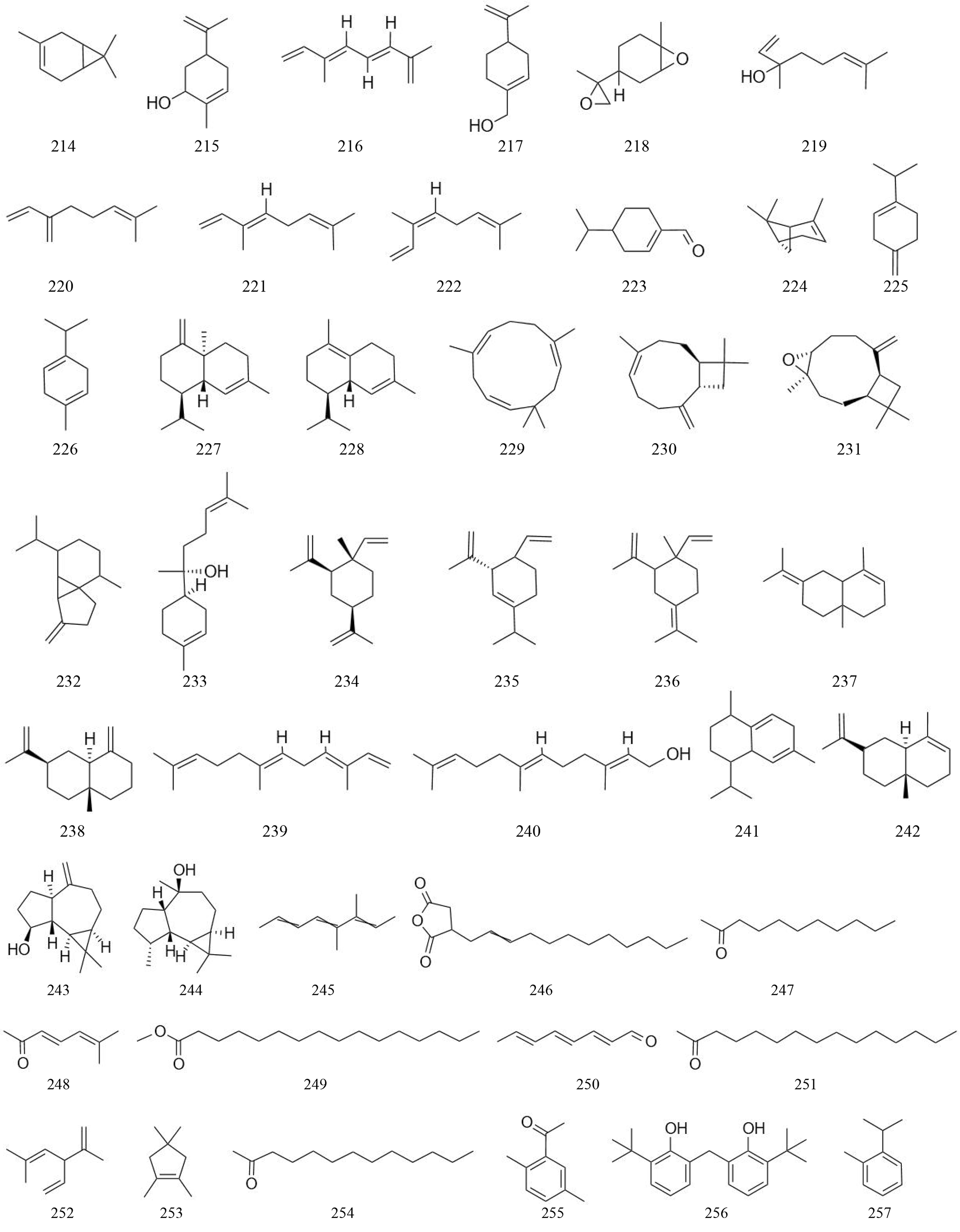

Supplement: Supplementary file 1 [file Image6.tif]

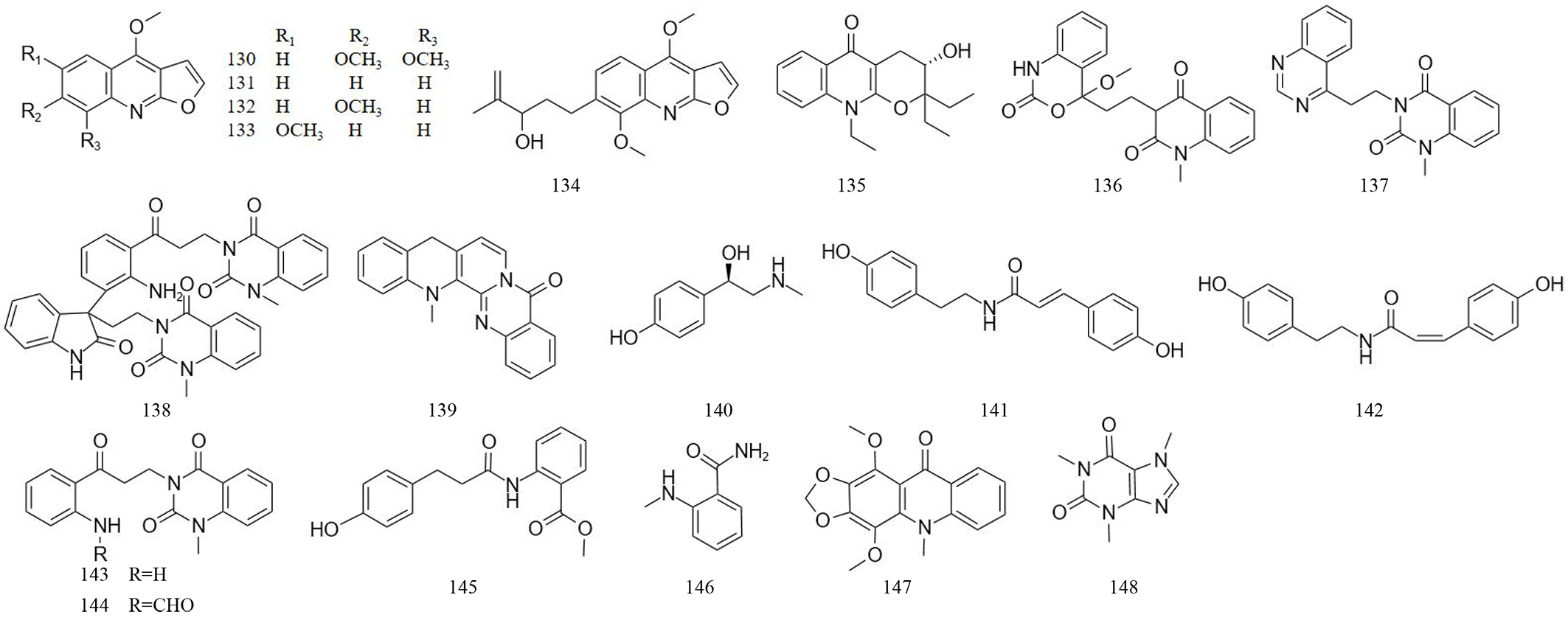

Supplement: Supplementary file 3 [file Image3.tif]

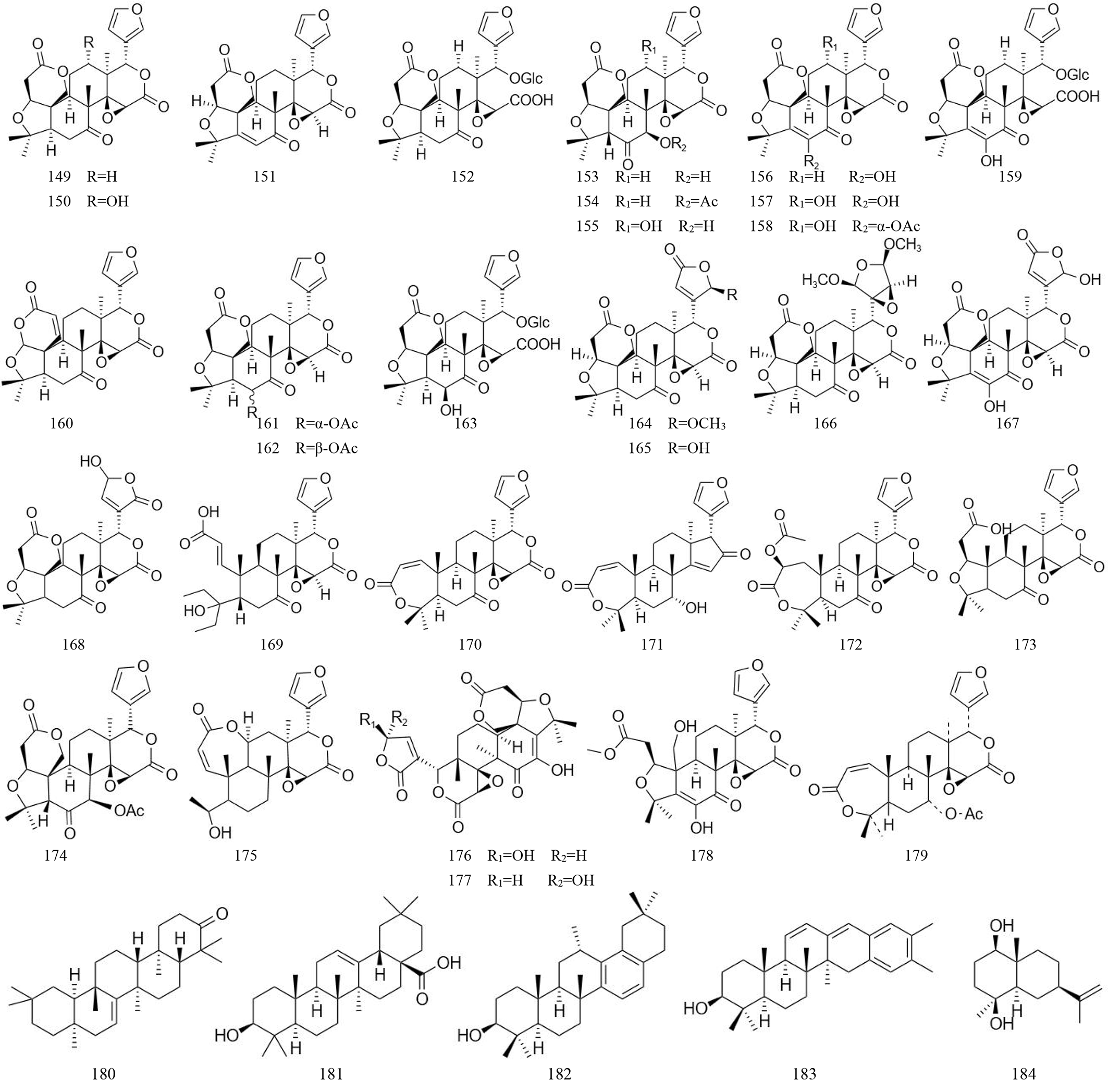

Supplement: Supplementary file 4 [file Image4.tif]

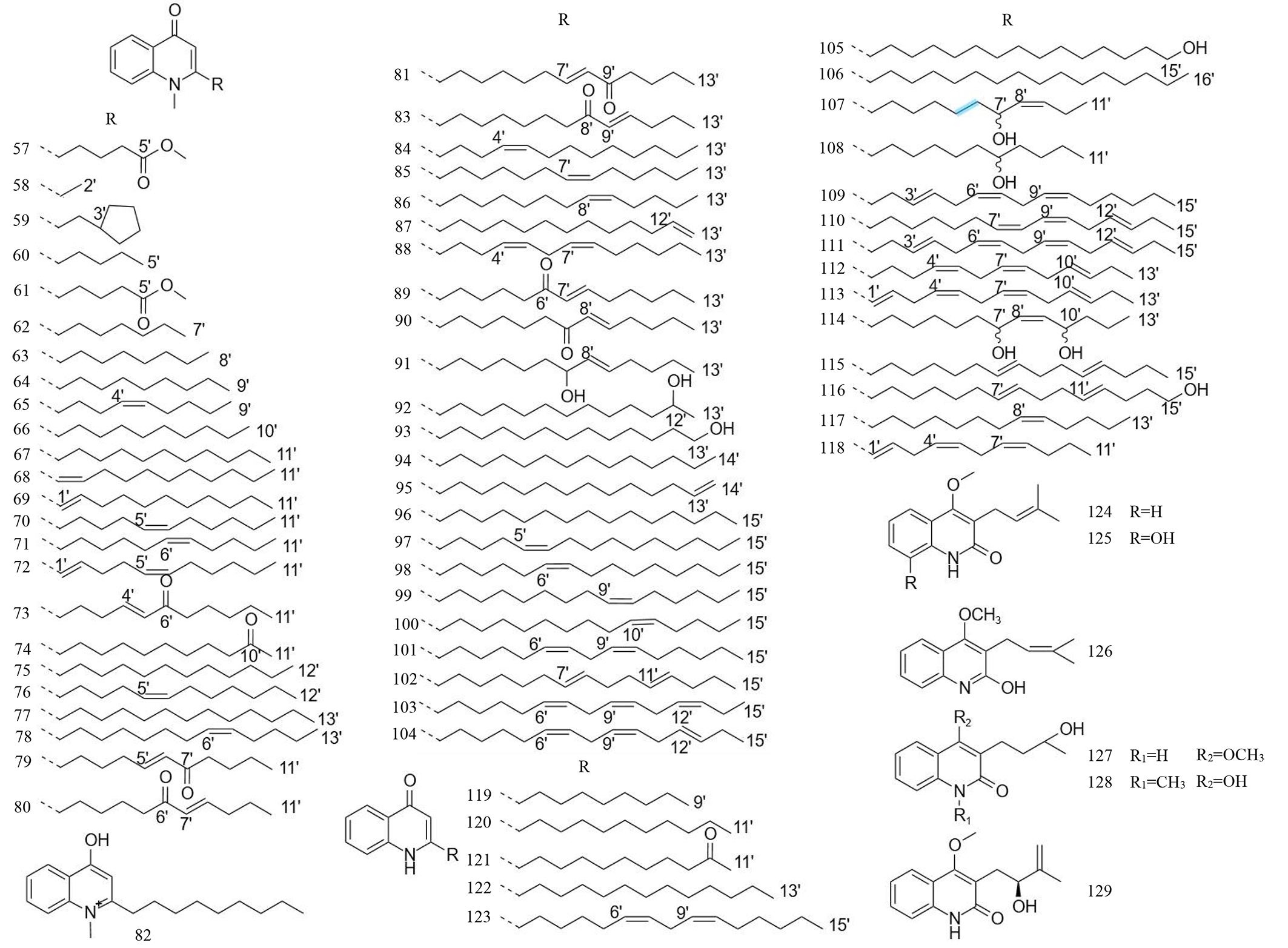

Supplement: Supplementary file 5 [file Image2.tif]

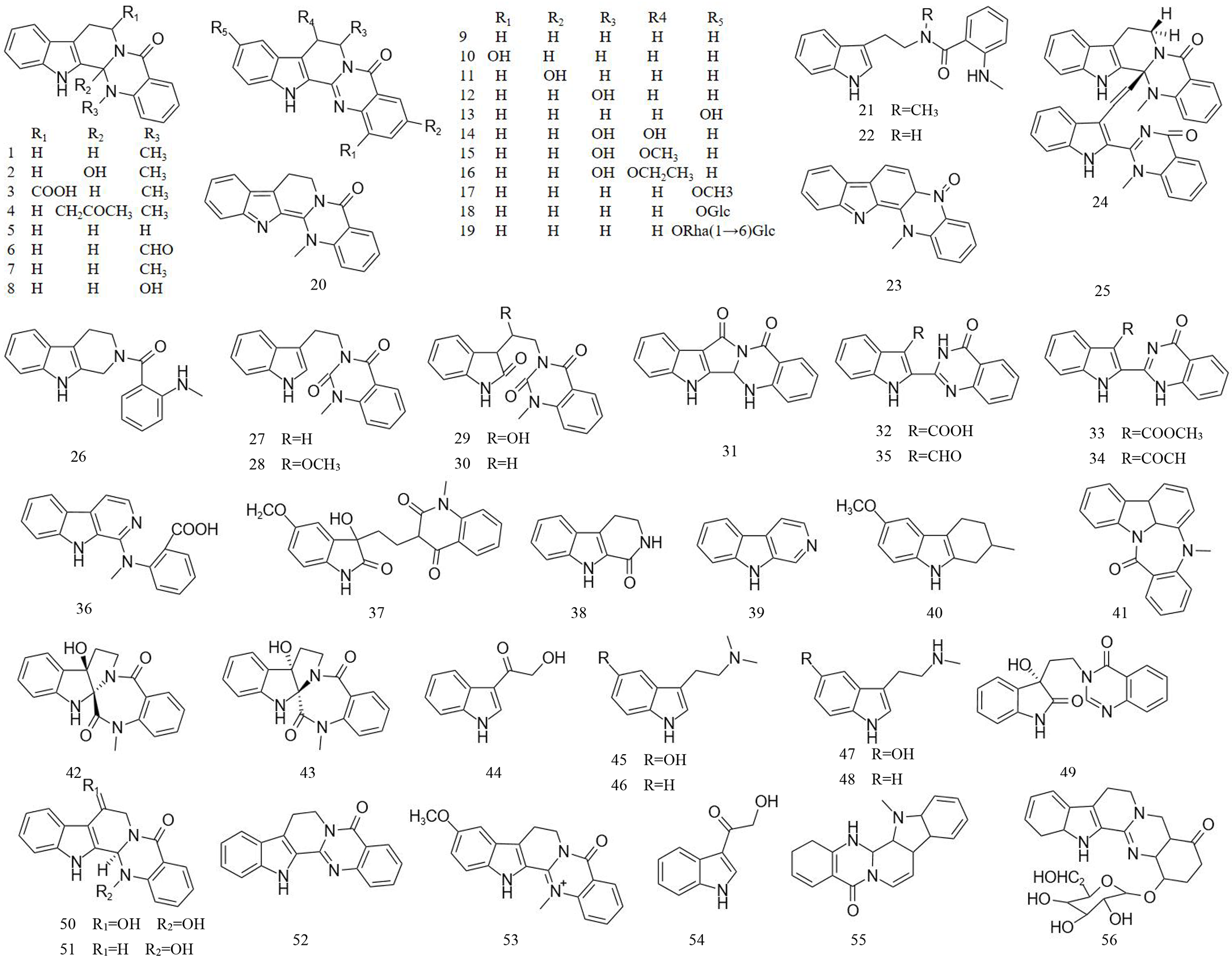

Supplement: Supplementary file 6 [file Image1.tif]

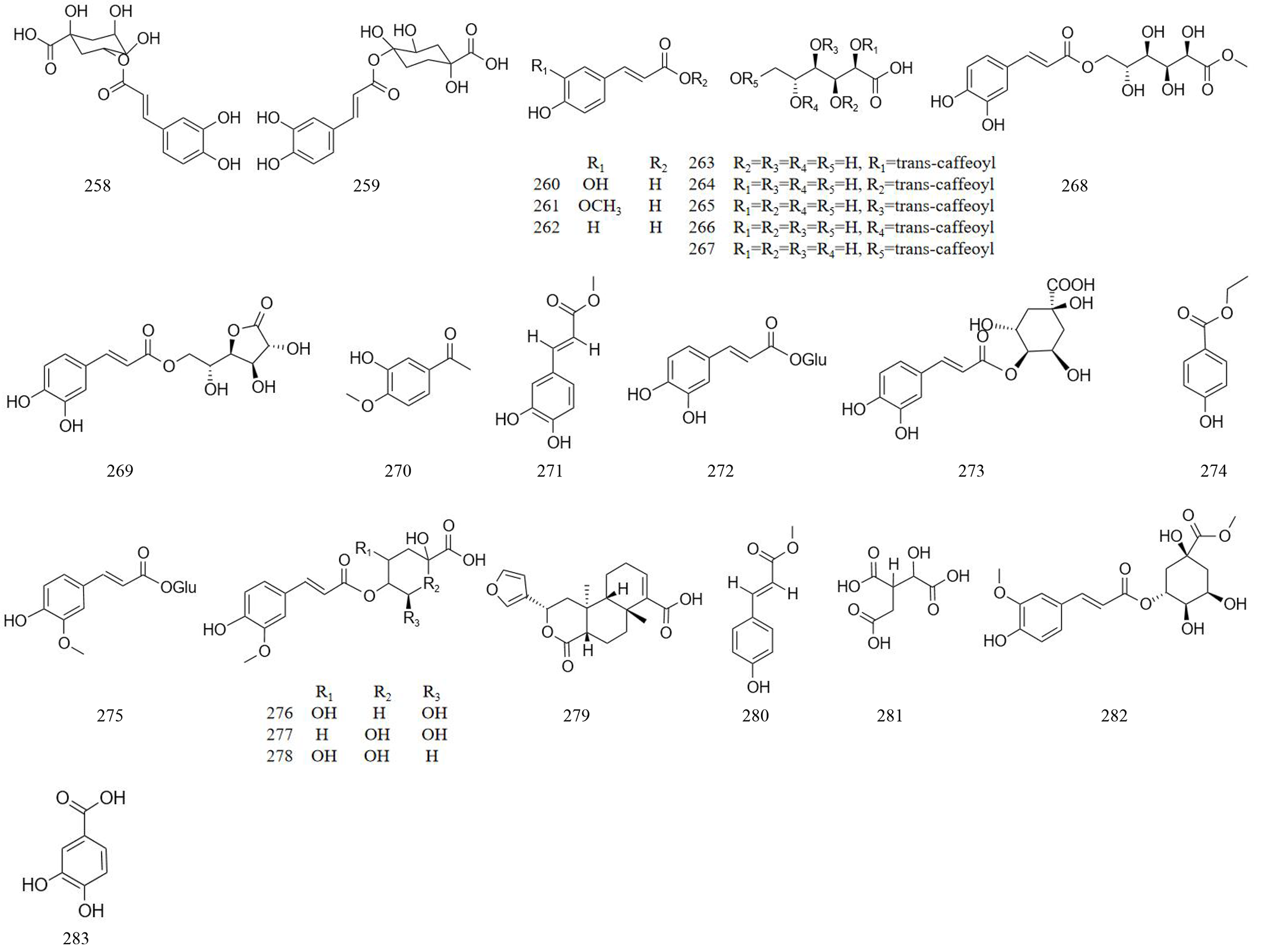

Supplement: Supplementary file 7 [file Image7.tif]

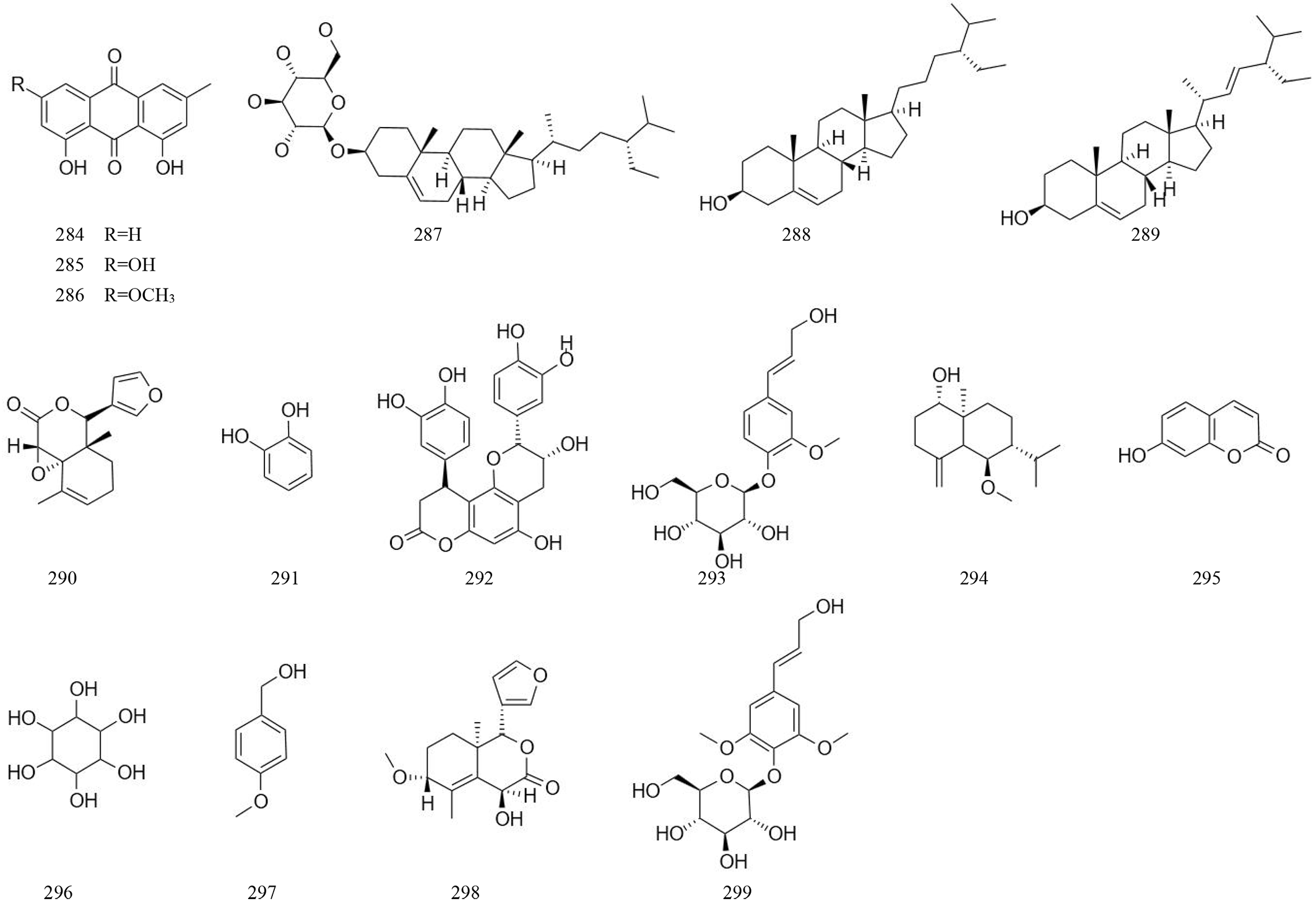

Supplement: Supplementary file 9 [file Image8.tif]

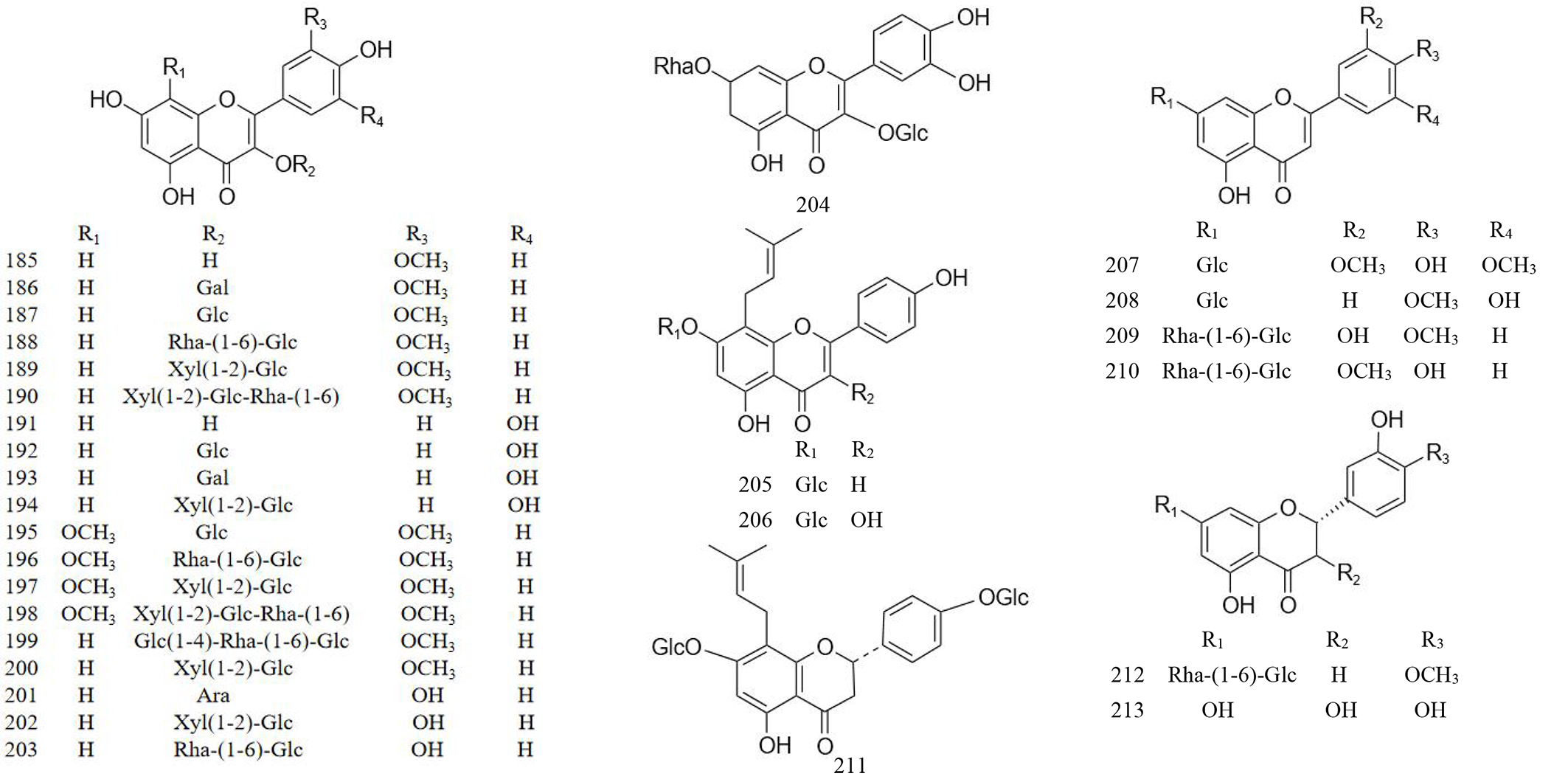

Supplement: Supplementary file 10 [file Image5.tif]
