# Supplementary material for: Euodiae Fructus: a review of botany, application, processing, phytochemistry, quality control, pharmacology, and toxicology
Source: Front Pharmacol. 2025 Jan 29;16:1509032. doi: 10.3389/fphar.2025.1509032 (PMC11813794; doi:10.3389/fphar.2025.1509032)
Supplement: Supplementary file 2 [file Table1.docx]

**Supplementary Table 1** Metabolites of EF.

| Classification | Number | Name | Molecular formula | Molecular weight | References |
| --- | --- | --- | --- | --- | --- |
| Alkaloids |  |  |  |  |  |
| Indoles | 1 | Evodiamine | C_19_H_17_N_3_O | 303.4 | ([Li and Wang, 2020](#_ENREF_70)) |
|  | 2 | 10-Hydroxyevodiamine | C_19_H_17_N_3_O_2_ | 319.4 | ([Li and Wang, 2020](#_ENREF_70)) |
|  | 3 | Carboxyevodiamine | C_20_H_17_N_3_O_3_ | 347.4 | ([Li and Wang, 2020](#_ENREF_70)) |
|  | 4 | Acetonylevodiamine | C_22_H_21_N_3_O_2_ | 359.4 | ([Li and Wang, 2020](#_ENREF_70)) |
|  | 5 | Dihydrorutaecarpine | C_18_H_15_N_3_O | 289.3 | ([Li and Wang, 2020](#_ENREF_70)) |
|  | 6 | 14-Formyldihydrorutaecarpine | C_20_H_18_N_2_O_2_ | 318.4 | ([Wang et al., 2010b](#_ENREF_158)) |
|  | 7 | 13b-Hydroxymethylevodiamine | C_20_H_19_N_3_O_2_ | 333.4 | ([Li and Wang, 2020](#_ENREF_70)) |
|  | 8 | 13b-Hydroxyevodiamine | C_19_H_17_N_3_O_2_ | 319.4 | ([Li and Wang, 2020](#_ENREF_70)) |
|  | 9 | Rutaecarpine | C_18_H_13_N_3_O | 287.3 | ([Li and Wang, 2020](#_ENREF_70)) |
|  | 10 | 1-Hydroxyrutaecarpine | C_18_H_13_N_3_O_2_ | 303.3 | ([Li and Wang, 2020](#_ENREF_70)) |
|  | 11 | 3-Hydoxyrutaecarpine | C_18_H_13_N_3_O | 303.3 | ([Li and Wang, 2020](#_ENREF_70)) |
|  | 12 | 7β-Hydroxyrutaecarpine | C_18_H_13_N_3_O | 303.3 | ([Li and Wang, 2020](#_ENREF_70)) |
|  | 13 | 10-Hydroxyrutaecarpine | C_18_H_13_N_3_O | 303.3 | ([Li and Wang, 2020](#_ENREF_70)) |
|  | 14 | (7*R*,8*S*)-7,8-Dihydroxy-rutaecarpine | C_18_H_13_N_3_O_3_ | 319.3 | ([Li and Wang, 2020](#_ENREF_70)) |
|  | 15 | (7*R*,8*S*)-7-Hydroxy-8-methoxy-rutaecarpine | C_19_H_15_N_3_O_3_ | 333.3 | ([Li and Wang, 2020](#_ENREF_70)) |
|  | 16 | (7*R*,8*S*)-7-Hydroxy-8-ethoxy-rutaecarpine | C_20_H_17_N_3_O_3_ | 347.4 | ([Li and Wang, 2020](#_ENREF_70)) |
|  | 17 | Hortiacine | C_19_H_15_N_3_O_2_ | 317.3 | ([Zhao et al., 2015a](#_ENREF_219)) |
|  | 18 | Rutaecarpine-10-*O*-β-*D*-glucopyranoside | C_24_H_23_N_3_O_7_ | 465.5 | ([Li and Wang, 2020](#_ENREF_70)) |
|  | 19 | Rutaecarpine-10-*O*-rutinoside | C_30_H_33_N_3_O | 451.6 | ([Li and Wang, 2020](#_ENREF_70)) |
|  | 20 | Dehydroevodiamine | C_19_H_15_N_3_O | 301.3 | ([Li and Wang, 2020](#_ENREF_70)) |
|  | 21 | Evodiamide | C_19_H_21_N_3_O | 307.3 | ([Li and Wang, 2020](#_ENREF_70)) |
|  | 22 | *N*-(2-methylaminobenzoyl)tryptamine | C_18_H_19_N_3_O | 293.37 | ([Zhao et al., 2015a](#_ENREF_219)) |
|  | 23 | Evodianinine | C_19_H_13_N_3_O | 299.3 | ([Li and Wang, 2020](#_ENREF_70)) |
|  | 24 | Dievodiamine | C_38_H_30_N_6_O_2_ | 602.7 | ([Li and Wang, 2020](#_ENREF_70)) |
|  | 25 | Rhetsinine | C_19_H_17_N_3_O_2_ | 319.4 | ([Li and Wang, 2020](#_ENREF_70)) |
|  | 26 | Goshuyuamide I | C_19_H_19_N_3_O | 305.4 | ([Li and Wang, 2020](#_ENREF_70)) |
|  | 27 | Goshuyuamide II | C_19_H_17_N_3_O₂ | 319.36 | ([Li and Wang, 2020](#_ENREF_70)) |
|  | 28 | 10-Methoxygoshuyuamide-II | C_20_H_19_N_3_O_3_ | 349.4 | ([Li and Wang, 2020](#_ENREF_70)) |
|  | 29 | Wuchuyuamide I | C_19_H_17_N_3_O_4_ | 351.4 | ([Li and Wang, 2020](#_ENREF_70)) |
|  | 30 | Wuchuyuamide II | C_19_H_17_N_3_O_3_ | 335.4 | ([Zuo et al., 2000](#_ENREF_234)) |
|  | 31 | Wuzhuyurutine A | C_17_H_11_N_3_O₂ | 289.29 | ([Li and Wang, 2020](#_ENREF_70)) |
|  | 32 | Wuzhuyurutine B | C_17_H_11_N_3_O_3_ | 305.29 | ([Li and Wang, 2020](#_ENREF_70)) |
|  | 33 | Wuzhuyurutine C | C_18_H_13_N_3_O_3_ | 319.3 | ([Li and Wang, 2020](#_ENREF_70)) |
|  | 34 | Wuzhuyurutine D | C_17_H_11_N_3_O_3_ | 305.29 | ([Li and Wang, 2020](#_ENREF_70)) |
|  | 35 | Bouchardatine | C_17_H_11_N_3_O_2_ | 289.29 | ([Li and Wang, 2020](#_ENREF_70)) |
|  | 36 | Evollionine A | C_19_H_15_N_3_O_2_ | 317.3 | ([Li and Wang, 2020](#_ENREF_70)) |
|  | 37 | Evollionine B | C_20_H_19_N_3_O_5_ | 381.4 | ([Li and Wang, 2020](#_ENREF_70)) |
|  | 38 | β-Carboline | C_11_H_8_N_2_ | 168.19 | ([Xiao et al., 2023](#_ENREF_172)) |
|  | 39 | 1,2,3,4-Tetrahydro-1-oxo-carboline | C_11_H_10_N_2_O | 186.21 | ([Xiao et al., 2023](#_ENREF_172)) |
|  | 40 | 6-Methoxy-*N*-methyl-1,2,3,4-tetrahydro-*β*-carboline | C_13_H_16_N_2_O | 216.28 | ([Li and Wang, 2020](#_ENREF_70)) |
|  | 41 | Evodiagenine | C_19_H_13_N_3_O | 299.3 | ([Wang et al., 2010b](#_ENREF_158)) |
|  | 42 | (-)-Evodiakine | C_19_H_17_N_3_O_3_ | 335.4 | ([Li and Wang, 2020](#_ENREF_70)) |
|  | 43 | (+)-Evodiakine | C_19_H_17_N_3_O_3_ | 335.4 | ([Li and Wang, 2020](#_ENREF_70)) |
|  | 44 | 3-Hydroxyacetylindole | C_10_H_9_NO_2_ | 175.18 | ([Xiao et al., 2023](#_ENREF_172)) |
|  | 45 | *N*-methyltryptamine | C_11_H_14_N_2_ | 174.24 | ([Xiao et al., 2023](#_ENREF_172)) |
|  | 46 | 5-Methoxy-*N*-methyltryptamine | C_12_H_16_N_2_O | 204.27 | ([Li and Wang, 2020](#_ENREF_70)) |
|  | 47 | *N*, *N*-Dimethyltryptamine | C_12_H_16_N_2_ | 218.29 | ([Xiao et al., 2023](#_ENREF_172)) |
|  | 48 | 5-Methoxy-*N*, *N*-dimethyltryptamine | C_13_H_18_N_2_O | 218.29 | ([Li and Wang, 2020](#_ENREF_70)) |
|  | 49 | (*S*)-7-Hydroxysecorutaecarpine | C_18_H_15_N_3_O_3_ | 321.3 | ([Li and Wang, 2020](#_ENREF_70)) |
|  | 50 | Evodamide A | C_19_H_15_N_3_O_2_ | 317.3 | ([Xiao et al., 2023](#_ENREF_172)) |
|  | 51 | 13,14-Dihydrorutecarpine | C_18_H_15_N_3_O | 289.3 | ([Li et al., 2020a](#_ENREF_67)) |
|  | 52 | *N*-formyldihydrorutaecarpine | C_19_H_15_N_3_O_2_ | 317.3 | ([Li and Wang, 2020](#_ENREF_70)) |
|  | 53 | Hortiamine | C_20_H_17_N_3_O_2_ | 331.4 | ([Li and Wang, 2020](#_ENREF_70)) |
|  | 54 | 2-Hydroxy-1-(1H-indol-3-yl)-ethanone | C_10_H_9_NO_2_ | 175.18 | ([He et al., 2024](#_ENREF_28)) |
|  | 55 | 13-Methyl-13*H*-indolo[2',3':3,4]pyrido[2,1-b]quinazolin-5-one | C_19_H_13_N_3_O | 299.3 | ([Li and Wang, 2020](#_ENREF_70)) |
|  | 56 | Rutaecarpine-1-*O*-β-*D*-glucopyranoside | C_24_H_23_N_3_O_7_ | 465.5 | ([Li and Wang, 2020](#_ENREF_70)) |
| Quinolones | 57 | Evollionine C | C_16_H_19_NO_3_ | 273.33 | ([Li et al., 2014](#_ENREF_75)) |
|  | 58 | 1-Methyl-2-ethyl-4(1*H*)-quinolone | C_12_H_13_NO | 187.24 | ([Wang et al., 2013a](#_ENREF_157)) |
|  | 59 | 1-Methyl-2-(2-cyclopentylethyl)-4(1*H*)-quinolinone | C_17_H_21_NO | 255.35 | ([Xiao et al., 2023](#_ENREF_172)) |
|  | 60 | 1-Methyl-2-pentyl-4-(1*H*)-quinolone | C_15_H_19_NO | 229.32 | ([Li and Wang, 2020](#_ENREF_70)) |
|  | 61 | 1-Methyl-2-heptyl-4(1*H*)-quinolone | C_17_H_23_NO | 257.37 | ([Li and Wang, 2020](#_ENREF_70)) |
|  | 62 | 1-Methyl-2-octyl-4(1*H*)-quinolone | C_18_H_25_NO | 271.4 | ([Li and Wang, 2020](#_ENREF_70)) |
|  | 63 | 1-Methyl-2-nonyl-4(1*H*)-quinolone | C_19_H_27_NO | 285.4 | ([Li and Wang, 2020](#_ENREF_70)) |
|  | 64 | 1-Methyl-2-[(*Z*)-4-nonenyl]-4(1*H*)-quinolone | C_19_H_25_NO | 283.4 | ([Xiao et al., 2023](#_ENREF_172)) |
|  | 65 | 1-Methyl-2-decyl-4(1*H*)-quinolone | C_20_H_29_NO | 299.4 | ([Li and Wang, 2020](#_ENREF_70)) |
|  | 66 | 1-Methyl-2-undecyl-4(1*H*)-quinolone | C_21_H_31_NO | 313.5 | ([Li and Wang, 2020](#_ENREF_70)) |
|  | 67 | 1-Methyl-2-[(*Z*)-1-undecenyl]-4(1*H*)-quinolone | C_21_H_29_NO | 311.5 | ([Xiao et al., 2023](#_ENREF_172)) |
|  | 68 | 1-Methyl-2-[(*E*)-1-undecenyl]-4(1*H*)-quinolone | C_21_H_29_NO | 311.5 | ([Li and Wang, 2020](#_ENREF_70)) |
|  | 69 | 1-Methyl-2-[(*Z*)-5-undecenyl]-4(1*H*)-quinolone | C_21_H_29_NO | 311.5 | ([Li and Wang, 2020](#_ENREF_70)) |
|  | 70 | 1-Methyl-2-[(*Z*)-6-undecenyl]-4(1*H*)-quinolone | C_21_H_29_NO | 311.5 | ([Li and Wang, 2020](#_ENREF_70)) |
|  | 71 | 1-Methyl-2-[(1*E*,5*Z*)-1,5-undecadienyl]-4(1*H*)-quinolone | C_21_H_27_NO | 309.4 | ([Li and Wang, 2020](#_ENREF_70)) |
|  | 72 | 1-Methyl-2-[6-carbonyl-(*E*)-4-undecenyl]-4(1*H*)-quinolone | C_21_H_29_NO_2_ | 327.5 | ([Li and Wang, 2020](#_ENREF_70)) |
|  | 73 | 1-Methyl-2-undecanone-10'-4(1*H*)-quinolone | C_21_H_31_NO_2_ | 329.5 | ([Xiao et al., 2023](#_ENREF_172)) |
|  | 74 | 1-Methyl-2-dodecyl-4-(1*H*)-quinolone | C_22_H_33_NO | 327.5 | ([Li and Wang, 2020](#_ENREF_70)) |
|  | 75 | 1-Methyl-2-[(*Z*)-5'-dodecenyl]-4(1*H*)-quinolone | C_22_H_31_NO | 325.5 | ([Li and Wang, 2020](#_ENREF_70)) |
|  | 76 | Dihydroevocarpine | C_23_H_35_NO | 341.5 | ([Li and Wang, 2020](#_ENREF_70)) |
|  | 77 | Evocarpine | C_22_H_33_NO | 327.5 | ([Li and Wang, 2020](#_ENREF_70)) |
|  | 78 | Euocarpine A | C_21_H_27_NO_2_ | 325.4 | ([Li and Wang, 2020](#_ENREF_70)) |
|  | 79 | Euocarpine B | C_21_H_27_NO_2_ | 325.4 | ([Li and Wang, 2020](#_ENREF_70)) |
|  | 80 | Euocarpine C | C_23_H_31_NO_2_ | 353.5 | ([Li and Wang, 2020](#_ENREF_70)) |
|  | 81 | Euocarpine D | C_23_H_31_NO_2_ | 353.5 | ([Li and Wang, 2020](#_ENREF_70)) |
|  | 82 | Euocarpine E | C_19_H_28_NO+ | 286.4 | ([Li and Wang, 2020](#_ENREF_70)) |
|  | 83 | 1-Methyl-2-[(*Z*)-4-tridecenyl]-4(1*H*)-quinolone | C_23_H_33_NO | 339.5 | ([Li and Wang, 2020](#_ENREF_70)) |
|  | 84 | 1-Methyl-2-[(*Z*)-7-tridecenyl]-4(1H)-quinolone | C_23_H_33_NO | 339.5 | ([Li and Wang, 2020](#_ENREF_70)) |
|  | 85 | 1-Methyl-2-[(*Z*)-8-tridecenyl]-4(1*H*)-quinolone | C_23_H_33_NO | 339.5 | ([Xiao et al., 2023](#_ENREF_172)) |
|  | 86 | 1-Methyl-2-[12-tridecenyl]-4(1*H*)-quinolone | C_22_H_33_NO | 339.5 | ([Li and Wang, 2020](#_ENREF_70)) |
|  | 87 | 1-Methyl-2-[(4*Z*,7*Z*)-4,7-tridecadienyl]-4(1*H*)-quinolone | C_23_H_31_NO | 337.5 | ([Li and Wang, 2020](#_ENREF_70)) |
|  | 88 | 1-Methyl-2-[6-carbonyl-(*E*)-7-tridecenyl]-4(1*H*)-quinolone | C_23_H_31_NO_2_ | 353.5 | ([Zhao et al., 2015a](#_ENREF_219)) |
|  | 89 | 1-Methyl-2-[7-carbonyl-(*E*)-9-tridecenyl]-4(1*H*)-quinolone | C_23_H_31_NO_2_ | 353.5 | ([Li and Wang, 2020](#_ENREF_70)) |
|  | 90 | 1-Methyl-2-[7-hydroxy-(*E*)-9-tridecenyl]-4(1*H*)-quinolone | C_23_H_33_NO_2_ | 355.5 | ([Xiao et al., 2023](#_ENREF_172)) |
|  | 91 | 1-Methyl-2-[12-hydroxy-tridecyl]-4(1H)-quinolone | C_23_H_35_NO_2_ | 357.5 | ([Li and Wang, 2020](#_ENREF_70)) |
|  | 92 | 1-Methyl-2-[13-hydroxyl-tridecenyl]-4(1*H*)-quinolone | C_23_H_35_NO_2_ | 357.5 | ([Li and Wang, 2020](#_ENREF_70)) |
|  | 93 | 1-Methyl-2-tetradecyl-4-(1*H*)-quinolone | C_24_H_37_NO | 355.6 | ([Li and Wang, 2020](#_ENREF_70)) |
|  | 94 | 1-Methyl-2-[13-tetradecenyl]-4-(1*H*)-quinolone | C_24_H_35_NO | 353.6 | ([Li and Wang, 2020](#_ENREF_70)) |
|  | 95 | 1-Methyl-2-pentadecyl-4(1*H*)-quinolone | C_25_H_39_NO | 369.6 | ([Li and Wang, 2020](#_ENREF_70)) |
|  | 96 | 1-Methyl-2-[(*Z*)-5'-pentadecenyl]-4(1*H*)-quinolone | C_25_H_37_NO | 367.6 | ([Li and Wang, 2020](#_ENREF_70)) |
|  | 97 | 1-Methyl-2-[(*Z*)-6-pentadecenyl]-4(1*H*)-quinolone | C_25_H_37_NO | 367.6 | ([Li and Wang, 2020](#_ENREF_70)) |
|  | 98 | 1-Methyl-2-[(*Z*)-9-pentadecenyl]-4(1*H*)-quinolone | C_25_H_37_NO | 367.6 | ([Li and Wang, 2020](#_ENREF_70)) |
|  | 99 | 1-Methyl-2-[(*Z*)-10-pentadecenyl]-4(1*H*)-quinolone | C_25_H_37_NO | 367.6 | ([Li and Wang, 2020](#_ENREF_70)) |
|  | 100 | 1-Methyl-2-[(6*Z*,9*Z*)-6,9-pentadecadienyl]-4(1*H*)-quinolone | C_25_H_35_NO | 365.6 | ([Li and Wang, 2020](#_ENREF_70)) |
|  | 101 | 1-Methyl-2-[(9*E*,13*E*)-heptadecadienyl]-4(1*H*)-quinolone | C_25_H_35_NO | 365.6 | ([Xiao et al., 2023](#_ENREF_172)) |
|  | 102 | 1-Methyl-2-[(6*Z*,9*Z*,12*Z*)-6,9,12-pentadecatriene]-4(1*H*)-quinolone | C_25_H_33_NO | 363.6 | ([Xiao et al., 2023](#_ENREF_172)) |
|  | 103 | 1-Methyl-2-[(6Z,9Z,12E)-pentadecatriene]-4(1*H*)-quinolone | C_25_H_33_NO | 363.6 | ([Li and Wang, 2020](#_ENREF_70)) |
|  | 104 | 1-Methyl-2-[15-hydroxyl-pentadecenyl]-4(1*H*)-quinolone | C_25_H_39_NO_2_ | 385.6 | ([Qin et al., 2021](#_ENREF_122)) |
|  | 105 | 1-Methyl-2-hexadecylol-4-(1*H*)-quinolone | C_26_H_41_NO | 383.6 | ([Li and Wang, 2020](#_ENREF_70)) |
|  | 106 | 1-Methyl-2-[7-hydroxy-(*E*)-9-undecenyl]-4(1*H*)-quinolone | C_21_H_29_NO_2_ | 327.5 | ([Zhao et al., 2021a](#_ENREF_218)) |
|  | 107 | 1-Methyl-2-[7-hydroxy-undecyl]-4(1*H*)-quinolone | C_21_H_31_NO_2_ | 329.5 | ([Zhao et al., 2021a](#_ENREF_218)) |
|  | 108 | 1-Methyl-2-[(3*E*,6*Z*,9*Z*)-3.6,9-pentadecenyl]-4(1*H*)-quinolone | C_25_H_33_NO | 363.6 | ([Zhao et al., 2021a](#_ENREF_218)) |
|  | 109 | 1-Methyl-3-[(7*E*,9*E*,12*Z*)-7,9,12-pentadecenyl]-4(1*H*)-quinolone | C_25_H_33_NO | 363.6 | ([Qin et al., 2021](#_ENREF_122)) |
|  | 110 | 1-Methyl-3-[(7*E*,9*E*,11*E*)-7,9,11-pentadecenyl]-4(1*H*)-quinolone | C_25_H_33_NO | 363.6 | ([Qin et al., 2021](#_ENREF_122)) |
|  | 111 | 1-Methyl-2-[(3*E*,6*Z*,9*Z*,12*E*)-3,6,9,12-pentadecenyl]-4(1*H*)-quinolone | C_25_H_31_NO | 361.6 | ([Zhao et al., 2021a](#_ENREF_218)) |
|  | 112 | 1-Methyl-2-[(4*Z*,7*Z*,10*E*-4,7,10-tridecenyl]-4(1*H*)-quinolone | C_23_H_29_NO | 335.5 | ([Zhao et al., 2021a](#_ENREF_218)) |
|  | 113 | 1-Methyl-2-[(1*E*,4*Z*,7*Z*,10*E*)-1,4,7,10-tridecenyl]-4(1*H*)-quinolone | C_23_H_27_NO | 333.5 | ([Zhao et al., 2021a](#_ENREF_218)) |
|  | 114 | 1-Methyl-2-[7,9-dihydroxy-(*Z*)-8-tridecenyl]-4(1*H*)-quinolone | C_23_H_33_NO_3_ | 339.5 | ([Zhao et al., 2021a](#_ENREF_218)) |
|  | 115 | 1-Methyl-2-[(9*E*,13*E*)-9,13-heptadecadienyl]-4(1*H*)-quinolone | C_27_H_39_NO | 393.6 | ([Li and Wang, 2020](#_ENREF_70)) |
|  | 116 | 1-Methyl-2-[15-hydroxyl-pentadecyl]-4(1*H*)- quinolinone | C_25_H_39_NO₂ | 369.6 | ([Li and Wang, 2020](#_ENREF_70)) |
|  | 117 | 1-Methyl-2-[(*Z*)-8-undecenyl]-4(1*H*)-quinolone | C_21_H_29_NO | 311.5 | ([Ma et al., 2021](#_ENREF_99)) |
|  | 118 | 1-Methyl-2-[(1*E*,4*Z*,7*Z*)-1,4,7-undecenyl]-4(1*H*)-quinolone | C_21_H_25_NO | 307.5 | ([Zhao et al., 2021a](#_ENREF_218)) |
|  | 119 | 2-Nonyl-4(1*H*)-quinolone | C_18_H_25_NO | 271.4 | ([Xiao et al., 2023](#_ENREF_172)) |
|  | 120 | 2-Undecyl-4(1*H*)-quinolone | C_20_H_29_NO | 299.4 | ([Li and Wang, 2020](#_ENREF_70)) |
|  | 121 | 2-Undecanone-10'-4(1*H*)-quinolone | C_20_H_27_NO_2_ | 313.4 | ([Li and Wang, 2020](#_ENREF_70)) |
|  | 122 | 2-Tridecyl-4(1*H*)-quinolone | C_22_H_31_NO | 325.5 | ([Xiao et al., 2023](#_ENREF_172)) |
|  | 123 | 2-[(6*Z*,9*Z*)-Pentadeca-6,9-dienyl]-quinolin-4(1*H*)-one | C_24_H_31_NO | 349.5 | ([Xiao et al., 2023](#_ENREF_172)) |
|  | 124 | Atanine | C_15_H_17_NO_2_ | 243.3 | ([Li and Wang, 2020](#_ENREF_70)) |
|  | 125 | 8-Hydroxy-4-methoxy-3-(3-methylbut-2-en-1-yl)quinolin-2(1*H*)-one | C_15_H_17_NO_3_ | 259.32 | ([Li et al., 2020a](#_ENREF_67)) |
|  | 126 | 2-Hydroxy-4-methoxy-3(3'-methyl-2'-butenyl)-quinolin | C_15_H_17_NO_2_ | 243.3 | ([Xiao et al., 2023](#_ENREF_172)) |
|  | 127 | 3-(3-Hydroxy-3-methylbutyl)-4-methoxyquinolin-2(1*H*)-one | C_15_H_19_NO_3_ | 261.32 | ([He et al., 2024](#_ENREF_28)) |
|  | 128 | 4-Hydroxy-3-(3-hydroxy-3-methylbutyl)quinolin-2(1*H*)-one | C_15_H_19_NO_3_ | 261.32 | ([He et al., 2024](#_ENREF_28)) |
|  | 129 | (*S*)-3-(2-Hydroxy-3-methylbut-3-en-1-yl)-4-methoxyquinolin-2(1*H*)-one | C_15_H_17_NO_3_ | 259.32 | ([Li et al., 2020a](#_ENREF_67)) |
| Quinolines | 130 | Skimmianine | C_14_H_13_NO_4_ | 259.26 | ([Li and Wang, 2020](#_ENREF_70)) |
|  | 131 | Dictamnine | C_12_H_9_NO_2_ | 199.2 | ([Xiao et al., 2023](#_ENREF_172)) |
|  | 132 | Evolitrine | C_13_H_11_NO_3_ | 229.23 | ([Xiao et al., 2023](#_ENREF_172)) |
|  | 133 | 6-Methoxydictamnine | C_13_H_11_NO_3_ | 229.23 | ([Xiao et al., 2023](#_ENREF_172)) |
|  | 134 | Evodine | C_18_H_19_NO_5_ | 329.3 | ([Xiao et al., 2023](#_ENREF_172)) |
|  | 135 | Ribalinine | C15H17NO3 | 259.3 | ([Xiao et al., 2023](#_ENREF_172)) |
| Organic amines | 136 | Evodiamide A | C_20_H_19_N_3_O_5_ | 381.4 | ([Li and Wang, 2020](#_ENREF_70)) |
|  | 137 | Evodiamide B | C_19_H_16_N_4_O_2_ | 332.4 | ([Li and Wang, 2020](#_ENREF_70)) |
|  | 138 | Evodiamide C | C_37_H_32_N_6_O_6_ | 656.7 | ([Xiao et al., 2023](#_ENREF_172)) |
|  | 139 | Evodiaxinine | C_20_H_15_N_3_O | 313.4 | ([Li and Wang, 2020](#_ENREF_70)) |
|  | 140 | Synephrine | C_9_H_13_NO_2_ | 167.2 | ([Li and Wang, 2020](#_ENREF_70)) |
|  | 141 | *N*-(*trans*-*p*-Coumaroyl)-tyramine | C_17_H_17_NO_3_ | 283.32 | ([Xiao et al., 2023](#_ENREF_172)) |
|  | 142 | *N*-(*cis*-*p*-Coumaroyl)-tyramine | C_17_H_17_NO_3_ | 283.32 | ([Xiao et al., 2023](#_ENREF_172)) |
|  | 143 | Wuchuyuamide III | C_18_H_17_NO_3_ | 295.3 | ([Li and Wang, 2020](#_ENREF_70)) |
|  | 144 | Wuchuyuamide IV | C_19_H_17_NO_4_ | 323.3 | ([Li and Wang, 2020](#_ENREF_70)) |
|  | 145 | Evodileptin B | C_17_H_17_NO_4_ | 299.32 | ([Kim et al., 2022](#_ENREF_50)) |
|  | 146 | 2-Methylamino-benzamide | C_8_H_10_N_2_O | 150.18 | ([Li and Wang, 2020](#_ENREF_70)) |
| Acridons | 147 | Melicopidine | C_17_H_15_NO_5_ | 313.3 | ([He et al., 2024](#_ENREF_28)) |
| Purines | 148 | Caffeine | C_8_H_10_N_4_O_2_ | 194.19 | ([He et al., 2024](#_ENREF_28)) |
| Terpenoids |  |  |  |  |  |
| Limonoids | 149 | Limonin | C_26_H_30_O_8_ | 470.5 | ([Li and Wang, 2020](#_ENREF_70)) |
|  | 150 | 12α-Hydroxylimonin | C_26_H_30_O_9_ | 486.5 | ([Li and Wang, 2020](#_ENREF_70)) |
|  | 151 | Dehydrolimonin | C_26_H_30_O_8_ | 470.5 | ([Li and Wang, 2020](#_ENREF_70)) |
|  | 152 | Limonin 17-β-*D*-glucopyranoside | C_32_H_42_O_14_ | 650.7 | ([Xiao et al., 2023](#_ENREF_172)) |
|  | 153 | Rutaevin | C_26_H_30_O_9_ | 486.5 | ([Li and Wang, 2020](#_ENREF_70)) |
|  | 154 | Rutaevin acetate | C_28_H_32_O_10_ | 528.5 | ([Li and Wang, 2020](#_ENREF_70)) |
|  | 155 | 12α-Hydroxyrutaevin | C_26_H_30_O_10_ | 502.5 | ([Li and Wang, 2020](#_ENREF_70)) |
|  | 156 | Evodol | C_26_H_28_O_9_ | 484.5 | ([Li and Wang, 2020](#_ENREF_70)) |
|  | 157 | 12α-Hydroxyevodol | C_26_H_28_O_10_ | 500.5 | ([Li and Wang, 2020](#_ENREF_70)) |
|  | 158 | 6α-Acetoxyl-12α-hydroxyevodol | C_28_H_32_O_11_ | 544.5 | ([He et al., 2024](#_ENREF_28)) |
|  | 159 | Limonin diosphenol 17-β-*D*-glucopyranoside | C_32_H_40_O_15_ | 664.6 | ([Zhao et al., 2015a](#_ENREF_219)) |
|  | 160 | Jangomolide | C_26_H_28_O_8_ | 468.5 | ([Li and Wang, 2020](#_ENREF_70)) |
|  | 161 | 6α-Acetoxy-5-epilimonin | C_28_H_32_O_10_ | 528.5 | ([Li and Wang, 2020](#_ENREF_70)) |
|  | 162 | 6β-Acetoxy-5-epilimonin | C_28_H_32_O_10_ | 528.5 | ([Li and Wang, 2020](#_ENREF_70)) |
|  | 163 | 6β-Hydroxy-5-epilimonin-17-β-*D*-glucopyranoside | C_32_H_42_O_15_ | 666.7 | ([Li and Wang, 2020](#_ENREF_70)) |
|  | 164 | Evorubodinin | C_27_H_32_O_10_ | 516.5 | ([Xiao et al., 2023](#_ENREF_172)) |
|  | 165 | Shihulimonin A | C_26_H_30_O_10_ | 502.5 | ([Li and Wang, 2020](#_ENREF_70)) |
|  | 166 | Evolimorutanin | C_28_H_36_O_11_ | 548.6 | ([Xiao et al., 2023](#_ENREF_172)) |
|  | 167 | Evodirutaenin | C_26_H_28_O_11_ | 516.5 | ([Li and Wang, 2020](#_ENREF_70)) |
|  | 168 | Isolimonexic acid | C_26_H_30_O_11_ | 518.5 | ([He et al., 2024](#_ENREF_28)) |
|  | 169 | Obacunonsaeure | C_26_H_32_O_8_ | 472.5 | ([He et al., 2024](#_ENREF_28)) |
|  | 170 | Obacunone | C_26_H_30_O_7_ | 454.5 | ([Li and Wang, 2020](#_ENREF_70)) |
|  | 171 | 7-Deacetylproceranone | C_26_H_31_O_5_ | 423.5 | ([Xiao et al., 2023](#_ENREF_172)) |
|  | 172 | Nomilin | C_28_H_34_O_9_ | 514.6 | ([Li and Wang, 2020](#_ENREF_70)) |
|  | 173 | Isoobacunoic acid | C_26_H_32_O_8_ | 472.5 | ([He et al., 2024](#_ENREF_28)) |
|  | 174 | 7β-Acetoxy-5-epilimonin | C_28_H_32_O_10_ | 528.5 | ([Qin et al., 2021](#_ENREF_122)) |
|  | 175 | Clauemargine L | C_26_H_30_O_8_ | 470.5 | ([Qin et al., 2021](#_ENREF_122)) |
|  | 176 | Euodirutaecin A | C_26_H_28_O_11_ | 516.5 | ([Qian et al., 2014](#_ENREF_121)) |
|  | 177 | Euodirutaecin B | C_26_H_28_O_11_ | 516.5([Qin et al., 2021](#_ENREF_122)) | ([Qian et al., 2014](#_ENREF_121)) |
|  | 178 | 19-Hydroxy methyl isoobacunoate diospheno | C_27_H_32_O_10_ | 516.5 | ([Qin et al., 2021](#_ENREF_122)) |
|  | 179 | 7α-Obacunyl acetate | C_28_H_34_O_9_ | 514.6 | ([Lacroix et al., 2011](#_ENREF_57)) |
| Others | 180 | Taraxerone | C_30_H_48_O | 424.7 | ([Xiao et al., 2023](#_ENREF_172)) |
|  | 181 | Oleanolicacid | C_30_H_48_O_3_ | 456.7 | ([Li and Wang, 2020](#_ENREF_70)) |
|  | 182 | Evoditrilone A | C_29_H_44_O | 408.7 | ([Li and Wang, 2020](#_ENREF_70)) |
|  | 183 | Evoditrilone B | C_29_H_44_O | 408.7 | ([Li and Wang, 2020](#_ENREF_70)) |
|  | 184 | 1β,4β-Dihydroxyeudesman-11-ene | C_15_H_26_O_2_ | 238.37 | ([Xiao et al., 2023](#_ENREF_172)) |
| Flavonoids |  |  |  |  |  |
| Flavonols | 185 | Isorhamnetin | C_16_H_12_O_7_ | 316.26 | ([Li and Wang, 2020](#_ENREF_70)) |
|  | 186 | Isorhamnetin-3-*O*-β-*D*-galactoside | C_22_H_12_O_12_ | 468.3 | ([Li and Wang, 2020](#_ENREF_70)) |
|  | 187 | Isorhamnetin-3-*O*-β-*D*-glucopyranoside | C_22_H_12_O_12_ | 468.3 | ([Li and Wang, 2020](#_ENREF_70)) |
|  | 188 | Isorhamnetin-3-*O*-rutinoside | C_28_H_32_O_16_ | 624.5 | ([Xiao et al., 2023](#_ENREF_172)) |
|  | 189 | Isorhamnetin-3-*O*-β-*D*-xylopyranosyl(1 → 2)-β-*D*-glucopyranoside | C_27_H_30_O_16_ | 610.5 | ([Li and Wang, 2020](#_ENREF_70)) |
|  | 190 | Isorhamnetin-3-*O*[2-*O*-β-*D*-xylopyranosyl-6-*O*-α-*L*-rhamnopyranosyl]-β-*D*-glucopyranoside | C_33_H_40_O_20_ | 756.7 | ([Xiao et al., 2023](#_ENREF_172)) |
|  | 191 | Quercetin | C_15_H_10_O_7_ | 302.23 | ([Li and Wang, 2020](#_ENREF_70)) |
|  | 192 | Isoquercitrin | C_21_H_20_O_12_ | 464.4 | ([Li and Wang, 2020](#_ENREF_70)) |
|  | 193 | Quercetin-3-*O*-β-*D*-galactoside (hyperoside) | C_21_H_20_O_12_ | 464.4 | ([Xiao et al., 2023](#_ENREF_172)) |
|  | 194 | Quercetin-3-*O*-β-*D*-xylopyranosyl(1 → 2)-β-*D*-glucopyranoside | C_26_H_38_O_17_ | 622.6 | ([Li and Wang, 2020](#_ENREF_70)) |
|  | 195 | Limocitrin-3-*O*-*β*-*D*-glucopyranoside | C_23_H_24_O_13_ | 508.4 | ([Li and Wang, 2020](#_ENREF_70)) |
|  | 196 | Limocitrin-3-*O*-rutinoside | C_29_H_34_O_17_ | 654.6 | ([Li and Wang, 2020](#_ENREF_70)) |
|  | 197 | Limocitrin-3-*O*-β-*D*-xylopyranosyl (1 → 2)-β-*D*-glucopyranoside | C_28_H_32_O_17_ | 640.5 | ([Li and Wang, 2020](#_ENREF_70)) |
|  | 198 | Limocitrin-3-*O*[2-*O*-β-*D*-xylopyranosyl-6-*O*-α-*L*-rhamnopyranosyl]-β-*D*-glucopyranoside | C_34_H_42_O_21_ | 786.7 | ([Xiao et al., 2023](#_ENREF_172)) |
|  | 199 | Isorhamnetin-3-rutinoside-4'-glucoside | C_34_H_42_O_21_ | 786.7 | ([Zhao et al., 2015a](#_ENREF_219)) |
|  | 200 | Isorhamnetin-3-*O*-sambubioside | C_27_H_30_O_16_ | 610.5 | ([Li and Wang, 2020](#_ENREF_70)) |
|  | 201 | Quercetin-3-*O*-α-*D*-arabinopyranoside | C_20_H_18_O_11_ | 434.3 | ([Zhao et al., 2015a](#_ENREF_219)) |
|  | 202 | Quercetin-3-*O*-sambubioside | C_26_H_28_O_16_ | 596.5 | ([Li and Wang, 2020](#_ENREF_70)) |
|  | 203 | Rutin | C_27_H_30_O_16_ | 610.5 | ([He et al., 2024](#_ENREF_28)) |
|  | 204 | Quercetin-3-*O*-β-*D*-ghucoside-7-*O*-α-*L*-thammanoside | C_27_H_30_O_16_ | 610.5 | ([Zhao et al., 2015a](#_ENREF_219)) |
|  | 205 | Phellodensin F | C_26_H_30_O_10_ | 502.5 | ([Xiao et al., 2023](#_ENREF_172)) |
|  | 206 | Epimedoside C | C_26_H_28_O_11_ | 516.5 | ([Xiao et al., 2023](#_ENREF_172)) |
| Flavonoids | 207 | Tricin-7-*O*-β-*D*-glucopyranoside | C_23_H_24_O_12_ | 492.4 | ([He et al., 2024](#_ENREF_28)) |
|  | 208 | Diosmetin-7-*O*-β-*D*-glucopyranoside | C_22_H_22_O_11_ | 462.4 | ([Li and Wang, 2020](#_ENREF_70)) |
|  | 209 | Diosmin | C_28_H_32_O_15_ | 608.5 | ([Li and Wang, 2020](#_ENREF_70)) |
|  | 210 | Chrysoeriol-7-*O*-rutinoside | C_28_H_32_O_15_ | 608.5 | ([Li and Wang, 2020](#_ENREF_70)) |
| Flavonones | 211 | Evodioside B | C_32_H_40_O_15_ | 664.6 | ([Xiao et al., 2023](#_ENREF_172)) |
|  | 212 | Hesperidin | C_28_H_34_O_15_ | 610.6 | ([Xiao et al., 2023](#_ENREF_172)) |
| Flavanols | 213 | Catechin | C_15_H_14_O_6_ | 290.27 | ([Xiao et al., 2023](#_ENREF_172)) |
| Volatile oils |  |  |  |  |  |
| Monoterpenoids | 214 | 3-Carene | C_10_H_16_ | 136.23 | ([He et al., 2024](#_ENREF_28)) |
|  | 215 | (*Z*)-carveol | C_10_H_16_O | 152.23 | ([He et al., 2024](#_ENREF_28)) |
|  | 216 | Cosmene | C_10_H_14_ | 134.22 | ([He et al., 2024](#_ENREF_28)) |
|  | 217 | Isocarveol | C_10_H_16_O | 152.23 | ([He et al., 2024](#_ENREF_28)) |
|  | 218 | Limonene dioxide | C_10_H_16_O_2_ | 168.23 | ([He et al., 2024](#_ENREF_28)) |
|  | 219 | Linalool | C_10_H_18_O | 154.25 | ([He et al., 2024](#_ENREF_28)) |
|  | 220 | Myrcene | C_10_H_16_ | 136.23 | ([He et al., 2024](#_ENREF_28)) |
|  | 221 | (*E*)-ocimene | C_10_H_16_ | 136.23 | ([Xiao et al., 2023](#_ENREF_172)) |
|  | 222 | (*Z*)-ocimene | C_10_H_16_ | 136.23 | ([Xiao et al., 2023](#_ENREF_172)) |
|  | 223 | Phellandra | C_10_H_16_O | 152.23 | ([He et al., 2024](#_ENREF_28)) |
|  | 224 | α-Pinene | C_10_H_16_ | 136.23 | ([He et al., 2024](#_ENREF_28)) |
|  | 225 | β-Terpinene | C_10_H_16_ | 136.23 | ([He et al., 2024](#_ENREF_28)) |
|  | 226 | g-Terpinene | C_10_H_16_ | 136.23 | ([He et al., 2024](#_ENREF_28)) |
| Sesquiterpenes | 227 | (+)-α-Bisabolol | C_15_H_26_O | 222.37 | ([He et al., 2024](#_ENREF_28)) |
|  | 228 | γ-Cadinene | C_15_H_24_ | 204.35 | ([He et al., 2024](#_ENREF_28)) |
|  | 229 | δ-Cadinene | C_15_H_24_ | 204.35 | ([He et al., 2024](#_ENREF_28)) |
|  | 230 | α-Caryophyllene | C_15_H_24_ | 204.35 | ([He et al., 2024](#_ENREF_28)) |
|  | 231 | β-Caryophyllene | C_15_H_24_ | 204.35 | ([He et al., 2024](#_ENREF_28)) |
|  | 232 | Caryophyllene oxide | C_15_H_24_O | 220.35 | ([He et al., 2024](#_ENREF_28)) |
|  | 233 | Cubebene | C_15_H_24_ | 204.35 | ([He et al., 2024](#_ENREF_28)) |
|  | 234 | β-Elemene | C_15_H_24_ | 204.35 | ([He et al., 2024](#_ENREF_28)) |
|  | 235 | δ-Elemene | C_15_H_24_ | 204.35 | ([He et al., 2024](#_ENREF_28)) |
|  | 236 | Elixene | C_15_H_24_ | 204.35 | ([He et al., 2024](#_ENREF_28)) |
|  | 237 | 3,7(11)-Eudesmadiene | C_15_H_24_ | 204.35 | ([He et al., 2024](#_ENREF_28)) |
|  | 238 | β-Eudesmene | C_15_H_24_ | 204.35 | ([He et al., 2024](#_ENREF_28)) |
|  | 239 | α-Farnesene | C_15_H_24_ | 204.35 | ([He et al., 2024](#_ENREF_28)) |
|  | 240 | Farnesyl alcohol | C_15_H_26_O | 222.37 | ([He et al., 2024](#_ENREF_28)) |
|  | 241 | 1,2,3,4,4a,7-Hexahydro-1,6-dimethyl-4-(1-methylethyl)-naphthalene | C_15_H_24_ | 204.35 | ([He et al., 2024](#_ENREF_28)) |
|  | 242 | α-Selinene | C_15_H_24_ | 204.35 | ([He et al., 2024](#_ENREF_28)) |
|  | 243 | Spathulenol | C_15_H_24_O | 220.35 | ([He et al., 2024](#_ENREF_28)) |
|  | 244 | Viridiflorol | C_15_H_26_O | 222.37 | ([He et al., 2024](#_ENREF_28)) |
| Aliphaties | 245 | 3,4-Dimethyl-2,4.6-octatriene | C_10_H_14_ | 134.22 | ([He et al., 2024](#_ENREF_28)) |
|  | 246 | 2-Dodecen-1-ylsuccinic-anhydride | C_16_H_26_O_3_ | 266.38 | ([He et al., 2024](#_ENREF_28)) |
|  | 247 | 2-Hendecanone | C_11_H_22_O | 170.29 | ([He et al., 2024](#_ENREF_28)) |
|  | 248 | 6-Methylhepta-3,5-dien-2-one | C_8_H_12_O | 124.18 | ([He et al., 2024](#_ENREF_28)) |
|  | 249 | Methylpalmitate | C_17_H_34_O₂ | 270.5 | ([He et al., 2024](#_ENREF_28)) |
|  | 250 | 2.4,6-Octatrienal | C_8_H_10_O | 122.16 | ([He et al., 2024](#_ENREF_28)) |
|  | 251 | 2-Pentadecanone | C_15_H_30_O | 226.4 | ([He et al., 2024](#_ENREF_28)) |
|  | 252 | Santolina triene | C_10_H_16_ | 136.23 | ([He et al., 2024](#_ENREF_28)) |
|  | 253 | 1,2,4,4-Tetramethyl-1-cyclopentene | C_9_H_16_ | 124.22 | ([He et al., 2024](#_ENREF_28)) |
|  | 254 | 2-Tridecanone | C_13_H_26_O | 198.34 | ([He et al., 2024](#_ENREF_28)) |
|  | 255 | 2,5-Dimethylacetophenone | C_10_H_12_O | 148.2 | ([He et al., 2024](#_ENREF_28)) |
|  | 256 | 2,2'-Methylenebis(4-methyl-6-tert-butylphenol) | C_23_H_33_O_2_ | 341.5 | ([He et al., 2024](#_ENREF_28)) |
|  | 257 | *O*-cymene | C_10_H_14_ | 134.22 | ([He et al., 2024](#_ENREF_28)) |
| Other categories |  |  |  |  |  |
| Organic acids | 258 | Neochlorogenic acid | C_16_H_18_O_9_ | 354.31 | ([He et al., 2024](#_ENREF_28)) |
|  | 259 | Chlorogenic acid | C_16_H_18_O_9_ | 354.31 | ([Zhao et al., 2015a](#_ENREF_219)) |
|  | 260 | Caffeic acid | C_9_H_8_O_4_ | 180.16 | ([He et al., 2024](#_ENREF_28)) |
|  | 261 | Ferulic acid | C_10_H_10_O_4_ | 194.18 | ([He et al., 2024](#_ENREF_28)) |
|  | 262 | *p*-Hydroxycinnamic acid | C_9_H_8_O_3_ | 164.16 | ([Li and Wang, 2020](#_ENREF_70)) |
|  | 263 | 2-*O*-*trans*-caffeoylgluconic acid | C_15_H_18_O_10_ | 358.3 | ([Li and Wang, 2020](#_ENREF_70)) |
|  | 264 | 3-*O*-*trans*-caffeoylgluconic acid | C_15_H_18_O_10_ | 358.3 | ([Li and Wang, 2020](#_ENREF_70)) |
|  | 265 | 4-*O*-*trans*-caffeoylgluconic acid | C_15_H_18_O_10_ | 358.3 | ([Li and Wang, 2020](#_ENREF_70)) |
|  | 266 | 5-*O*-*trans*-caffeoylgluconic acid | C_15_H_18_O_10_ | 358.3 | ([Li and Wang, 2020](#_ENREF_70)) |
|  | 267 | 6-*O*-*trans*-caffeoylgluconic acid | C_15_H_18_O_10_ | 358.3 | ([Li and Wang, 2020](#_ENREF_70)) |
|  | 268 | *trans*-Caffeoyl-6-*O*-*D*-gluconic acid methyl ester | C_16_H_20_O_10_ | 372.32 | ([Li and Wang, 2020](#_ENREF_70)) |
|  | 269 | *trans*-Caffeoyl-6-*O*-*D*-glucono-γ-lactone | C_15_H_16_O_9_ | 340.28 | ([Li and Wang, 2020](#_ENREF_70)) |
|  | 270 | Citric acid | C_6_H_8_O_7_ | 192.12 | ([Li and Wang, 2020](#_ENREF_70)) |
|  | 271 | Caffeic acid methyl ester | C_10_H_10_O_4_ | 194.18 | ([Li and Wang, 2020](#_ENREF_70)) |
|  | 272 | 1-*O*-caffeoyl-*D*-glucoside | C_15_H_18_O_9_ | 342.3 | ([Li and Wang, 2020](#_ENREF_70)) |
|  | 273 | Cryptochlorogenic acid | C_16_H_18_O_9_ | 354.31 | ([Zhao et al., 2015a](#_ENREF_219)) |
|  | 274 | Ethylparaben | C_9_H_10_O_3_ | 166.17 | ([Li and Wang, 2020](#_ENREF_70)) |
|  | 275 | *trans*-Feruloylgluconic acid | C_16_H_20_O_10_ | 372.32 | ([Zhao et al., 2015a](#_ENREF_219)) |
|  | 276 | 3-*O*-Feruloylquinic acid | C_17_H_20_O_9_ | 368.3 | ([Zhao et al., 2015a](#_ENREF_219)) |
|  | 277 | 4-*O*-Feruloylquinic acid | C_17_H_20_O_9_ | 368.3 | ([Zhao et al., 2015a](#_ENREF_219)) |
|  | 278 | 5-*O*-Feruloylquinic acid | C_17_H_20_O_9_ | 368.3 | ([Zhao et al., 2015a](#_ENREF_219)) |
|  | 279 | Floribundic acid | C_20_H_24_O_5_ | 344.4 | ([Zhao et al., 2015a](#_ENREF_219)) |
|  | 280 | trans-4-Hydroxycinnamic acid methyl ester | C_10_H_10_O_3_ | 178.18 | ([Xiao et al., 2023](#_ENREF_172)) |
|  | 281 | Isocitric acid | C_6_H_8_O_7_ | 192.12 | ([He et al., 2024](#_ENREF_28)) |
|  | 282 | Methyl-3-*O*-feruloylquinate | C_18_H_22_O_9_ | 382.4 | ([Zhao et al., 2015a](#_ENREF_219)) |
|  | 283 | Protocatechuic acid | C_7_H_6_O_4_ | 154.12 | ([He et al., 2024](#_ENREF_28)) |
| Anthraquinones | 284 | Chrysophanol | C_15_H_10_O_4_ | 254.24 | ([Li and Wang, 2020](#_ENREF_70)) |
|  | 285 | Emodin | C_15_H_10_O_5_ | 270.24 | ([Li and Wang, 2020](#_ENREF_70)) |
|  | 286 | Physcion | C_16_H_12_O_5_ | 284.26 | ([Li and Wang, 2020](#_ENREF_70)) |
| Steroid | 287 | β-Daucosterol | C_35_H_60_O_6_ | 576.8 | ([Li and Wang, 2020](#_ENREF_70)) |
|  | 288 | β-Sitosterol | C_25_H_50_O | 366.7 | ([Li and Wang, 2020](#_ENREF_70)) |
|  | 289 | β-Stigmasterol | C_29_H_48_O | 412.7 | ([Li and Wang, 2020](#_ENREF_70)) |
| Others | 290 | Calodendrolide | C_15_H_16_O_4_ | 260.28 | ([Li and Wang, 2020](#_ENREF_70)) |
|  | 291 | Catechol | C_6_H_6_O_2_ | 110.11 | ([He et al., 2024](#_ENREF_28)) |
|  | 292 | Cinchonain | C_24_H_20_O_9_ | 452.4 | ([He et al., 2024](#_ENREF_28)) |
|  | 293 | Coniferin | C_16_H_22_O_8_ | 342.34 | ([Li and Wang, 2020](#_ENREF_70)) |
|  | 294 | Hiiranlactone E | C_16_H_28_O_2_ | 252.39 | ([Xiao et al., 2023](#_ENREF_172)) |
|  | 295 | 7-Hydroxycoumarin | C_9_H_6_O_3_ | 162.14 | ([He et al., 2024](#_ENREF_28)) |
|  | 296 | *myo*-Inositol | C_6_H_12_O_6_ | 180.16 | ([He et al., 2024](#_ENREF_28)) |
|  | 297 | 4-Methoxybenzyl alcohol | C_8_H_10_O_2_ | 138.16 | ([He et al., 2024](#_ENREF_28)) |
|  | 298 | 9α-Methoxyl dictamdio | C_16_H_20_O_5_ | 292.33 | ([Qin et al., 2021](#_ENREF_122)) |
|  | 299 | Syringin | C_17_H_24_O_9_ | 372.4 | ([Li and Wang, 2020](#_ENREF_70)) |
