# Supplementary material for: Euodiae Fructus: a review of botany, application, processing, phytochemistry, quality control, pharmacology, and toxicology
Source: Front Pharmacol. 2025 Jan 29;16:1509032. doi: 10.3389/fphar.2025.1509032 (PMC11813794; doi:10.3389/fphar.2025.1509032)
Supplement: Supplementary file 8 [file Table2.docx]

**Supplementary Table 2** Pharmacological study of EF and its metabolites.

| Pharmacological action | Type of study | Drug | Experimental model | Mode of drug administration | Dose range tested | Duration | Results (-: upward; +: downward) | References |  |
| --- | --- | --- | --- | --- | --- | --- | --- | --- | --- |
| Cardiovascular protection | In vitro | EF aqueous extract | Isolated male Wistar rat's thoracic aortas | - | 1-300 μg/mL | - | (-): The expression of adrenergic and serotonergic receptors. | ([Hibino et al., 2009b](#_ENREF_31)) |  |
|  | In vivo | Evodiamine | Male Wistar rats | Lp | 0.1 mL/kg | 120 min | (-): The expression of PPARα protein.  (+): The level of ATP and (Ca^2+^)-ATPase activity. | ([Xue et al., 2015](#_ENREF_176)) | |
|  | In vitro | Evodiamine | MOVAS cells | - | 0.5 μmol/L | 3 days | (-): The expression of p-PI3K and p-AKT protein. | ([Zha et al., 2023](#_ENREF_198)) | |
|  | In vitro | Evodiamine | Neonatal rat's colon tissues | - | 2.3 mg | 24 h | (+): The level of DNMT1, DNMT3A and DNMT3B; the expression of miR-152, miR-429 and miR-29a. | ([Huang et al., 2017](#_ENREF_34)) | |
|  | In vitro | Rutaecarpine | Human endothelial EA. hy926 cells | - | 10 μmol/L | 90 min | (+): The level of NO, eNOS, CaMKKβ, AMPK, CaMKII; Ca^2+^ concentration. | ([Lee et al., 2021](#_ENREF_60)) | |
|  | In vitro | Rutaecarpine | HEK 293 cells | - | 25 mg/kg/d | 24 h | (-): Threonine/tyrosine phosphorylation of Sp1. | ([Zhan et al., 2021](#_ENREF_199)) | |
|  | In vitro | Limonin | HEK 293 cells | - | 1 mL | 24 h | (-): The expression of Sp1 and hERG protein; hERG current. (+): The hERG channel inactivation. | ([Li et al., 2022c](#_ENREF_73)) | |
|  | In vivo | Limonin | Pressure overload-induced cardiac hypertrophy in C57BL/6 mice | Oral | 30, 80 mg/kg | 28 days | (+): The cardiac function and heart weight. | ([Liu et al., 2022](#_ENREF_85)) | |
|  | In vitro | Limonin | NRCMs | - | 5, 10, 25, 50, 100, 150, 200 μmol/L | 24 h | (-): The degradation of SIRT6 protein. | ([Liu et al., 2022](#_ENREF_85)) | |
| Gastrointestinal protection | In vivo | Evodiamine | Ethanol-induced gastric ulcer in BALB/c mice | Oral | 20, 40 mg/kg | 1 h | (-): MDA content and MPO activity; the protein expressions of Ras homology, Ras homology-kinase 1 and 2, cytosolic and nucleic NF-κB p65. (+): The levels of GSH, SOD and catalase. | ([Zhao et al., 2015b](#_ENREF_222)) | |
|  | In vitro | Evodiamine | Helicobacter pylori ATCC 49503 strain and AGS cells | - | 0.5-40 μmol/L | 72 h | (-): The NF-κB and MAPK pathway. | ([Yang et al., 2021](#_ENREF_184)) | |
|  | In vivo | Evodiamine | Dextran Sulfate Sodium Salt (DSS)-induced ulcerative colitis (UC) in rats | Oral | 20 mg/kg | 7 days | (-): Destruction of epithelial integrity; inflammatory response. | ([Wang et al., 2020](#_ENREF_155)) | |
|  | In vivo | Evodiamine | Water avoidance stress and sham water avoidance stress rats | Oral | 0.67, 2, 6 mg/kg | 7 days | (+): The release of CCK and activation of the CCK1 receptor. | ([Ren et al., 2018](#_ENREF_125)) | |
|  | In vivo | Rutaecarpine | Ethanol-induced gastric mucosal injury in mice | Oral | 450, 900 μg/kg | 3 days | (-): The expression of NF-κB p65, TNF-α, IL-6, IL-1β, MPO, Bax, and caspase-3; the level of MDA; the gross gastric damage, ulcer index, and the histopathology damage. (+): The expression of NQO1, HO-1 and Bcl-2; the level of SOD, GSH and catalase. | ([Ren et al., 2020](#_ENREF_129)) | |
|  | In vivo | Rutaecarpine | Ethanol-induced gastric mucosal injury in rats | Lp | 0.6, 1.2 mg/kg | 30 min | (-): The level of asymmetric dimethylarginine. | ([Liu et al., 2008](#_ENREF_93)) | |
|  | In vitro | Rutaecarpine | Isolated SD rats gastric tissues | - | 1, 10 μmol/L | 10 min | (+): The release of CGRP and NO. | ([Liu et al., 2008](#_ENREF_93)) | |
|  | In vivo | Dehydroevodiamine | Indoleamine 2,3-dioxygenase-induced gastric injury in rats | Oral | 10, 20, 40 mg/kg | 7 days | (-): The food intake reduction, weight loss, and gastric injury; the expression of MPO, TNF-α, and IL-6; the phosphorylation of ERK and p38. (+): The expression of IL-10. | ([Wei et al., 2021](#_ENREF_164)) | |
|  | In vivo | Dehydroevodiamine | MNNG-induced GES-1 | Oral | 5, 10 mg/kg | 4 weeks | (-): Serum biochemical indices; histological damage of gastric tissue; migration and invasion of GES-1. (+): GES-1 proliferation. | ([Wen et al., 2021](#_ENREF_166)) | |
|  | In vivo | EF polysaccharide | Ethanol-induced gastric ulcer in mice | Oral | 100, 200, 400 mg/kg BW | 2 weeks | (-): The expression of Keap1. (+): SOD and GSH-Px activities; GSH content and IL-10 level; the expression of Nrf2 and HO-1 protein. | ([Luo et al., 2023](#_ENREF_97)) | |
| Neuroprotection | In vivo | EF methanol extract | Ischemia-induced neuronal and cognitive impairment in rats | Oral | 200 mg/kg | 2 weeks | (-): The loss of cholinergic immunoreactivity; neuronal and cognitive impairment. | ([Lee et al., 2011](#_ENREF_58)) | |
|  | In vivo | Evodiamine and rutaecarpine | C57BL/6 mice | Lp | 0.30 and 0.29 mg/kg, respectively | 30 min | (-): The peripheral hypersensitivity and anxiety. | ([Zhang et al., 2020](#_ENREF_210)) | |
|  | In vivo | Evodiamine | Intracerebroventricular injection of streptomycin-induced AD in mice | Oral | 50, 100 mg/kg | 21 days | (-): Acetylcholinesterase activity, GSH and SOD activities; the level of TNF-α, IL-1β, IL-6 and malondialdehyde level. | ([Wang et al., 2018a](#_ENREF_150)) | |
|  | In vivo | Evodiamine | The wild-type C57BL/6 J and 3×Tg AD mice | Lp | 200 μg/kg every other day | 4 weeks | (-): Tau protein hyperphosphorylation. (+): The working memory, spatial learning, and memory; the expression of glutamate N1, glutamate A2. | ([Wan et al., 2024](#_ENREF_148)) | |
|  | In vitro | Evodiamine | SH-SY5Y and HepaG2 cells | - | 0.0001, 0.001, 0.01, 0.1, 1.0, 10, 50, 100 μg/mL | 24 h | (-): The cell death induced by H2O2; the cytotoxicity induced by Aβ oligomers. | ([Wan et al., 2024](#_ENREF_148)) | |
|  | In vitro | Evodiamine | L-glutamate-induced HT22 cells | - | 5, 10, 20, 40 μmol/L | 3 h | (-): The accumulation of ROS; the expression levels of Bax and cleaved-caspase-8/3. (+): The cell viability; the Bcl-2 protein content; the phosphorylation activities of PKB and mTOR. | ([Zhang et al., 2018](#_ENREF_214)) | |
|  | In vivo | Evodiamine | D-galactose and aluminum trichloride-developed AD in mice | Lp | 10, 40 mg/kg | 42 days | (-): The aimless and chaotic movements; the deposition of Aβ42; the level of acetylcholinesterase.  (+): The serum level of Aβ42, acetylcholine and choline acetyltransferase. | ([Zhang et al., 2018](#_ENREF_214)) | |
|  | In vivo | Rutaecarpine | Middle cerebral artery occlusion rats | Oral | 5, 10, 20 mg/kg | 4 weeks | (-): Neuronal injury induced by cerebral I/R; the activation of caspase-3 and the expression of Bax; the phosphorylation of ERK1/2. (+): The expression of Nrf2, HO-1 and NQO1. | ([Han et al., 2019](#_ENREF_25)) | |
|  | In vitro | Rutaecarpine | Dorsal root ganglia obtained from SD rats | - | 0.1-10 μmol/L | - | (-): The TRPV1 receptor antagonist capsazepine. (+): The mRNA expression of CGRPα and CGRPβ. | ([Yang et al., 2018b](#_ENREF_189)) | |
|  | In vitro | Rutaecarpine | 293 cells | - | 0.1, 1, 10 μmol/L | 4 weeks | (+): The level of Ca^2+^; the activation of TRPV1. | ([Yang et al., 2018b](#_ENREF_189)) | |
|  | In vivo | Limonin | 6-hydroxydopamine-injected rats | Oral | 100 mg/kg | 6 weeks | (-): Motor activity and parkinson disease-related pathological damage. | ([Gao et al., 2023](#_ENREF_22)) | |
|  | In vitro | Limonin | BV2 and PC12 cells | - | 2, 4, 8 μmol/L | 18-24 h | (-): The excessive autophagy of neurons; microglial inflammation. | ([Gao et al., 2023](#_ENREF_22)) | |
|  | In vivo | Dehydroevodiamine | Scopolamine-induced amnesia and Aβ-infused in rats | Oral | 10 mg/kg | 21 days | (-): Neurotoxicity; the level of ROS; intracellular calcium levels. | ([Shin et al., 2017](#_ENREF_139)) | |
|  | In vivo | Dehydroevodiamine | Tg2576 and wild-type mice | Lp | 0.5 mg/kg | 4 weeks | (-): Cortical levels of soluble Aβ40, soluble Aβ42 and total Aβ peptides. | ([Shin et al., 2016](#_ENREF_140)) | |
| Anti-inflammation | In vivo | EF 70% ethanol extract | House dust mite-induced atopic dermatitis in NC/Nga mice | Oral | 1, 3 mg | 4 weeks | (-): The dermatitis score; epidermal hyperplasia and thickening; mast cell infiltration; plasma levels of histamine and corticosterone; the expression of thymic stromal lymphopoietin, CD4^+^ T cells and IL-4. | ([Jin et al., 2024](#_ENREF_47)) | |
|  | In vitro | EF 70% ethanol extract | HaCaT cells | - | 2.5, 10 μg/mL | - | (-): The activation of JAK-STAT and MAPK signaling pathways. | ([Jin et al., 2024](#_ENREF_47)) | |
|  | In vivo | ER aqueous extract | Hyperuricemia and gouty arthritis in mice with potassium oxonate and monosodium urate crystals | Oral | 0.375, 0.75, 1.5 g/kg/d | 1 week | (-): The level of IL-1β and TNF-α, the expression of renal urate anion transporter 1 and glucose transporter 9. (+): The activation of NLRP3 inflammasome signaling. | ([Wang et al., 2024b](#_ENREF_162)) | |
|  | In vitro | EF 70% ethanol extract and rutaecarpine | Angiotensin-converting enzyme 2-overexpressing HEK 293 cells | - | 0.1, 1, 10, 25, 50, 100, and 1, 10, 100 µmol/L, respectively | 24 h | (-): The 3C-like protease activity. | ([Lin et al., 2023](#_ENREF_83)) | |
|  | In vitro | Rutaecarpine | LPS-induced RAW 264.7 cells | - | 10, 20 µmol/L | 20 min | (-): The expression of iNOS protein, COX-2, TNF-α and IL-1β. | ([Jayakumar et al., 2021](#_ENREF_41)) | |
|  | In vitro | Rutaecarpine | Imiquimod-induced psoriasis-like dermatitis in BALB/c mice | - | 0.1%, 0.5%, 1% | 5 days | (-): TLR7 signaling activation; The levels of IL-23 p19, IL-17 A, IL-6 and interferon-α; the protein levels of TLR7, TNF receptor associated factor 6 and p65. | ([Li et al., 2019b](#_ENREF_74)) | |
|  | In vivo | Evodiamine | DSS-induced UC in C57BL/6 mice | Oral | 10, 30 mg/kg/d | 3 weeks | (-): The level of IL-1β, IL-2, IL-6, IL-8 and TNF-α. | ([Zhang et al., 2022c](#_ENREF_215)) | |
|  | In vivo | Evodiamine | Adenomatous polyposis coli /Gpt C57BL/6 mice with colorectal cancer | Oral | 10 mg/kg every other day | 8 weeks | (-): The level of IL-17 and IL-22. (+): The level of IL-15. | ([Zhang et al., 2022c](#_ENREF_215)) | |
|  | In vivo | Evodiamine | LPS-induced mastitis in BALB/c mice | Oral | 50 mg/kg/d | 1 h | (-): The level of IL-1β, TNF-α, iNOS and NF‑κB; MAPK signaling pathways phosphorylation. | ([Yang et al., 2022b](#_ENREF_190)) | |
|  | In vitro | Evodiamine | Mouse mammary epithelial cells | - | 5, 10 mmol/L | 4 h | (-): The expression of AKT, NF-κB p65, ERK1/2, p38 and JNK. | ([Yang et al., 2022b](#_ENREF_190)) | |
|  | In vivo | Evodiamine | DSS-induced UC in C57BL/6 mice | Oral | 20, 40, 80 mg/kg | 5 days | (-): The level of TNF-α, IL-1β and IL-6; MPO activity; the expression of NF-κB p65 and NLRP3 inflammasome. (+): The expression of zonula occludens-1 and occludin protein. | ([Shen et al., 2019](#_ENREF_133)) | |
|  | In vivo | Limonin | High-fat diet mice | Oral | 30, 60, 90 mg/kg | - | (-): Steatosis and lipid accumulation of liver; the expression of p-STAT3/STAT3, caspase-8 and prostaglandin-endoperoxide synthase 2. | ([Wang et al., 2023b](#_ENREF_159)) | |
|  | In vivo | Limonin | TCR transgenic DO11.10 Rag2−/− mice | Oral | 0.02% | 2 weeks | (-): NF-κB p65 nuclear translocation in activated CD4^+^ T-cells. | ([Wang et al., 2023b](#_ENREF_159)) | |
|  | In vivo | Limonin | Metabolic syndrome was induced in rats by 10% fructose in water and 3% salt in diet | Oral | 50 mg/kg | 4 weeks | (-): The level of TGF-β1; excessive aortic collagen deposition; vascular fibrosis. | ([Hassan et al., 2018](#_ENREF_27)) | |
|  | In vivo | Limonin | LPS-induced acute lung injury in ICR mice | Oral | 10 mg/kg | 2 h | (-): The level of TNF-α, IL-1β and IL-6; the expression of TNF-α mRNA, IL-1β mRNA, IL-6 mRNA, TLR4 and NF- κB p65 protein. | ([Wang et al., 2018b](#_ENREF_151)) | |
| Analgesia | In vivo | EF methanol extract | Male Wistar rats and male ddY mice | Oral | 50, 200 mg/kg | 5 h | (+): Nociceptive threshold of the inflamed paw. | ([Matsuda et al., 1997](#_ENREF_102)) | |
|  | In vivo | Evodiamine | Male adult SD rats | Lp | 50 µl | 10 min | (-): Capsaicin-induced currents; thermal hyperalgesia. | ([Iwaoka et al., 2016](#_ENREF_39)) | |
|  | In vivo | Evodiamine | Nitroglycerin-induced acute migraine in rats | Oral | 45, 90 mg/kg | 9 days | (-): Serum NO levels; the migraine-like pain response. | ([Lin et al., 2020](#_ENREF_82)) | |
|  | In vivo | Evodiamine | Paclitaxel-induced peripheral neuropathy rats | Lp | 5 mg/kg | 3 days | (-): The level of interleukin IL-1β, IL-6 and TNF-α. (+): The expression of PPAR-gamma coactivator-1α, uncoupling protein 2 and SOD2. | ([Wu and Chen, 2019](#_ENREF_168)) | |
|  | In vivo | Limonin | Male Wistar rats and male ddY mice | Oral | 30, 100 mg/kg | 30 min | (-): The rise of vascular permeability, paw edema, and ear swelling. | ([Matsuda et al., 1998](#_ENREF_103)) | |
| Anti-tumor | In vitro | EF 70% ethanol extract | HeLa human cervical carcinoma cells | - | 5, 10, 20, 30, 40, 50, 60 μg/ml | 24 h | (-): Anti-apoptotic Bcl-2 expression; the cell viability of HeLa cells. (+): Activity of caspase-9/3. | ([Park et al., 2017](#_ENREF_116)) | |
|  | In vitro | Evodiamine | A549 and NCI-H522 cells | - | 5, 10, 15, 20, 25, 30 μg/ml | 72 h | (-): EMT; the expression of SOX9 and β-catenin. | ([Panda et al., 2024a](#_ENREF_112)) | |
|  | In vitro | Evodiamine | A549 and H1299 NSCLC cells | - | 1, 2, 4, 8, 16 μmol/L | 72 h | (-): Thr cell viability and migration; the expression of γ-secretase; Notch3 activation; tumor growth. | ([Yang et al., 2020b](#_ENREF_188)) | |
|  | In vitro | Evodiamine | HepG2, SMMC-7721 and H22 cells | - | 8, 16, 32, 64, 128 μmol/L | 24 and 48 h | (-): Tumor specific growth factor and alpha-fetoprotein activities; cell viability; the level of p-AKT.  (+): The number of apoptotic cells; the expression of bax/bcl-2 and cleaved-caspase-3. | ([Yang et al., 2017a](#_ENREF_183)) | |
|  | In vitro | Evodiamine | Human ovarian cancer cells HO-8910PM | - | 1.25, 2.5, 5 μmol/L | 4 days | (-): The expression of Bcl-2, PI3K, AKT, ERK1/2 MAPK and p38 MAPK.  (+): The level of cyclin B1 and Bax. | ([Wei et al., 2016](#_ENREF_163)) | |
|  | In vitro | Evodiamine | MCF-7 and MDA-MB-231 cells | - | 100, 200, 500, 1000, and 0.4, 0.8, 1.6, 3.2, 6.4 μmol/L, respectively | 48 h | (+): The expression of p53 and p21. | ([Han et al., 2016](#_ENREF_26)) | |
|  | In vitro | Evodiamine | HuCCT-1 and TFK-1 cells | - | 5, 10, 20, 40 μmol/L | 72 h | (-): Cholangiocarcinoma cell migration and invasion; IL-6/STAT3 signaling. (+): The expression levels of SHP-2. | ([Zhu et al., 2019](#_ENREF_231)) | |
|  | In vitro | Limonin | HCT116 and SW480 cells | - | 10-60 μmol/L | 24 h | (-): Cell proliferation, migration, invasion, colony formation, and tumor formation; the phosphorylation of STAT3 at Y705. | ([Zhang et al., 2024b](#_ENREF_211)) | |
|  | In vivo | Dehydroevodiamine | PANC-1 human pancreatic cancer xenografts in female BALB/c nude mice | Lp | 30, 50 mg/kg every three days | 30 days | (-): The self-renewal capabilities of prostate cancer stem cells; the expression of stemness-associated proteins. | ([Zhu et al., 2024](#_ENREF_233)) | |
|  | In vitro | Dehydroevodiamine | PANC-1 and MIA PaCa-2 cells | - | 100, 200, 300 μmol/L | 72 h | (-): Pancreatic ductal adenocarcinoma cell proliferation and tumor growth. | ([Zhu et al., 2024](#_ENREF_233)) | |
| Regulating glucose and lipid metabolism | In vivo | Evodiamine | Male SD rats | Oral | 4, 40 mg/kg | 25 days | (-): The rate of food intake and body weight increase, as well as orexigenic NPY and AgRP mRNA levels and NPY peptide level.  (+): The circulating level of leptin | ([Shi et al., 2009](#_ENREF_136)) | |
|  | In vivo | Evodiamine and EF 70 % ethanol extract | Male C3H mice and male SD rats | Oral | 0.03% and 0.02%, respectively | 12 and 21 days, respectively | (-): The body weight; the perirenal and epididymal fat weight; the levels of serum free fatty acid, cholesterol level, total lipids, and triglyceride in the liver. | ([Kobayashi et al., 2001](#_ENREF_54)) | |
|  | In vitro | Evodiamine | 3T3-L1 adipocytes and 293T cells | - | 50, 100, 150, 200 μmol/L | 48 and 1 h, respectively | (+): Activation of AMPK, adiponectin polymerization and the (Ca^2+^)-dependent PI3K/AKT/CaMKII signaling pathway | ([Liu et al., 2014](#_ENREF_86)) | |
|  | In vivo | Evodiamine | HFD-induced KM mice | Oral | 10, 30 mL/kg/d | 4 weeks | (-): The level of TG, TC, LDL-C, and blood viscosity. (+): The level of HDL-C. | ([Zhang et al., 2017](#_ENREF_207)) | |
|  | In vivo | Evodiamine | HFD and HCD-induced SD rats | Oral | 178.4 mg/kg/d | 4 weeks | (-): The level of TC, TG, LDL-C, and β-sitosterol; the expression of ACAT2, apoB-48, and NPC1L1. | ([Zhou et al., 2017b](#_ENREF_227)) | |
|  | In vivo | Evodiamine | HFD-induced male SD rats | Oral | 16.6 mg/kg | 4 weeks | (-): The level of TC, TG, LDL-C; the expressions of intestinal NPC1L1, ACAT2 and apoB-48. | ([Zhou et al., 2017a](#_ENREF_226)) | |
|  | In vivo | Rutaecarpine | HFD-induced C57BL/6 and leptin-deficient (ob/ob) obese mice | Oral | 20 mg/kg BW and 0.01%, respectively | 2 d and 4 weeks, respectively | (-): The level of blood cholesterol, non-fasting glucose, insulin, and leptin; the expression of the orexigenic neuropeptides NPY and AgRP. | ([Kim et al., 2009a](#_ENREF_51)) | |
|  | In vivo | Evodiamine | Male C57BL/6J mice | Oral | 1, 10 mg/kg | - | (-): The body weight gain. (+): The glucose tolerance. | ([Yamashita et al., 2015](#_ENREF_177)) | |
|  | In vivo | Evodiamine | Obese/diabetic KK-Ay mice | Oral | 3 mg/kg BW | 7 days | (-): The mTOR-S6K signaling and IRS1 serine phosphorylation. | ([Wang et al., 2013a](#_ENREF_157)) | |
|  | In vitro | Evodiamine | 3T3-L1 adipocytes cells | - | 20 μmol/L | 4 h | (-): The mTOR-S6K signaling and IRS1 serine phosphorylation. | ([Wang et al., 2013a](#_ENREF_157)) | |
|  | In vivo | Evodiamine and rutaecarpine | Male C57BL/6J, leptin receptor-mutated (db/db), leptin-deficient (ob/ob), and CAR knockout (CAR-/-) mice | Oral | 1, 2, 5, and 1, 2, 10 μmol/L, respectively | 48 h | (-): Hepatic steatosis and lipogenic; The expression of gluconeogenic, phosphoenolpyruvate carboxykinase and glucose-6-phosphatase. | ([Yu et al., 2016](#_ENREF_195)) | |
|  | In vitro | Evodiamine and rutaecarpine | Human hepatoma HepG2 cells | - | 1, 2, 5, and 1, 2, 10 μmol/L, respectively | 48 h | (-): Hepatic steatosis and lipogenic; The expression of gluconeogenic, phosphoenolpyruvate carboxykinase and glucose-6-phosphatase. | ([Yu et al., 2016](#_ENREF_195)) | |
|  | In vivo | Rutaecarpine | The fat-fed/STZ-treated rats | Oral | 25 mg/kg/d | 7 weeks | (-): Obesity, visceral fat accumulation, water consumption, the level of TC, TG, and LDL-C, NF-κB, TNF-α, and IL-6; IRS1 phosphorylation. (+): PI3K p85 subunit levels and AKT/PKB phosphorylation. | ([Nie et al., 2016](#_ENREF_108)) | |
|  | In vitro | Rutaecarpine | Cultured skeletal muscle cells | - | 20-180 μmol/L | 24 and 48 h | (+): The phosphorylation of AMPK and acetyl-CoA carboxylase 2; glucose uptake. | ([Nie et al., 2016](#_ENREF_108)) | |
| Protective effect of liver and kidney | In vitro | Evodiamine | L8824 cells | - | 10 μmol/L | 24 h | (-): The level of ROS and the occurrence of oxidative stress; Nrf2/MAPK pathway; apoptosis of grass carp hepatocytes. (+): The dissociation of Nrf2. | ([Xiong et al., 2022](#_ENREF_174)) | |
|  | In vivo | Evodiamine | CCl-induced liver fibrosis in rats | Oral | 15, 25 mg/kg | 8 weeks | (-): The level of AST, ALT and total bilirubin; Concentrations of IL-6, TNF-α collagen-I and collagen-III; the expression of TGF-β1, p-Smad 2/3 and α-smooth muscle actin. | ([Yang et al., 2018a](#_ENREF_182)) | |
|  | In vitro | Rutaecarpine | Human hepatoma HepG2 cells | - | 1-10 μmol/L | 1 h | (-): ROS production, cytotoxicity and apoptosis. | ([Jin et al., 2017](#_ENREF_48)) | |
|  | In vivo | Rutaecarpine | TBHP-induced male ICR mice | Oral | 5 mg/kg | 3 days | (-): The serum levels of AST, ALT, and lipid peroxidation. (+): The phosphorylation of AKT and CaMKII; the expression of HO-1. | ([Jin et al., 2017](#_ENREF_48)) | |
|  | In vivo | Rutaecarpine | Acetaminophen-induced hepatotoxicity in mice | Oral | 5, 20 mg/kg | 7 days | (-): ALT/AST activities and hepatic MDA content; the expression of CYP2E1; NF-κB activation by JNK1/2. (+): Nrf2-mediated activation of the antioxidant enzymes HO-1 and NQO1. | ([Choi et al., 2021](#_ENREF_11)) | |
|  | In vivo | Limonin | Male C57BL/6 mice | Oral | 7.5, 15, 30 g/kg | 24 h | (-): The level of ALT, AST, ALP, LDH, MDA, MPO, TNF-α, IL-1β and IL-6. (+): The level of GSH and SOD. | ([Zhang et al., 2021c](#_ENREF_209)) | |
|  | In vitro | Limonin | Acetaminophen-induced liver injury L-02 cells | - | 10, 25, 50 μmol/L | 2 h | (-): The ratio of Bax/Bcl-2; ROS production and cleavage of caspase-3. (+): MMP. | ([Yang et al., 2020a](#_ENREF_186)) | |
|  | In vivo | Limonin | Acetaminophen-induced liver injury in C57BL/6 mice | Oral | 40, 80 mg/kg | 1 h | (-): NF-κB inflammatory response. (+): Nrf2 antioxidative signals. | ([Yang et al., 2020a](#_ENREF_186)) | |
|  | In vivo | Evodiamine | I/R induced renal injury in male Wistar albino rats | Lp | 10 mg/kg | 24 h | (-): The level of IL-10, TAS, caspase-3 and NF-κB. | ([Eraslan et al., 2019](#_ENREF_16)) | |
|  | In vivo | Evodiamine | LPS-induced acute kidney injury in rats | Lp | 100, 200 mg/kg | 24 h | (-): The levels of blood urea nitrogen, creatinine, TNF-α, and IL-1; the expression of NF-κB p65; ROS production. | ([Shi et al., 2019](#_ENREF_137)) | |
|  | In vivo | Rutaecarpine | I/R injury rats | Lp | 30, 60 mg/kg | 2 and 24 h | (-): The serum creatinine content, urea nitrogen, neutrophil gelatinase-associated lipocalin and inflammatory cytokines. (+): The serum SOD levels. | ([Wang et al., 2017](#_ENREF_149)) | |
|  | In vivo | Limonin | IRI mice | Oral | 40, 80 mg/kg | 4 days | (-): The cell apoptosis. (+): The tubular cell proliferation. | ([Zhou et al., 2023](#_ENREF_228)) | |
|  | In vivo | Limonin | Cisplatin-induced AKI in mice | Oral | 80 mg/kg | 3 days | (-): The level of linoleic acid and arachidonic acid; the activity of CYP3A4. | ([Zeng et al., 2023](#_ENREF_196)) | |
|  | In vitro | Evodiamine | L8824 cells | - | 10 μmol/L | 24 h | (-): The level of ROS and the occurrence of oxidative stress; Nrf2/MAPK pathway; apoptosis of grass carp hepatocytes. (+): The dissociation of Nrf2. | ([Xiong et al., 2022](#_ENREF_174)) | |
| Insecticidal and antibacterial | In vivo | EF ethyl acetate extract | Goldfish-*Gyrodactylus kobayashii* Mode | Oral | 100 mg/L | 48 h | EC_50_ = 24.0 mg/L. | ([Lian et al., 2019](#_ENREF_78)) | |
|  | In vivo | EF petroleum ether extract | Goldfish-*Gyrodactylus kobayashii* Mode | Oral | 100 mg/L | 48 h | EC_50_ = 71.9 mg/L. | ([Lian et al., 2019](#_ENREF_78)) | |
|  | In vivo | EF methanol extract | Goldfish-*Gyrodactylus kobayashii* Mode | Oral | 100 mg/L | 48 h | EC_50_ = 40.9 mg/L. | ([Lian et al., 2019](#_ENREF_78)) | |
|  | In vitro | EF essential oil | Maize weevils, *Sitophilus zeamais*, and red flour beetle *Tribolium castaneum* | - | - | 1 week | LC_50_ = 36.89, 24.57, and 57.31 mg/L air, respectively. | ([Liu and Du, 2011](#_ENREF_94)) | |
|  | In vitro | Evodiamine and rutaecarpine | The larvae of *Drosophila melanogaster* Meigen | - | 0.11, 0.28, 0.84, 1.40 μmol/mL | 7 days | LC_50_ = 0.30 and 0.28 μmol/L, respectively. | ([Miyazawa et al., 2002](#_ENREF_105)) | |
|  | In vitro | Evodiamine and rutaecarpine | The early fourth instar larvae of *Aedes albopictus* | - | 6.3, 12.5, 25, 50, 100 μg/mL | - | LC_50_ = 12.51 and 17.02 μg/mL, respectively. | ([Liu et al., 2012](#_ENREF_95)) | |
|  | In vitro | Limonin, evodol, and EF ethanol extract | The Asian tiger mosquitoes | - | 6.3, 12.5, 25, 50, 100 μg/mL | - | LC_50_ = 32.43, 52.22, and 43.21 μg/mL, respectively. | ([Liu et al., 2012](#_ENREF_95)) | |
|  | In vitro | Evodiamine, rutaecarpine and EF 70% ethanol extract | *Meloidogyne incognita* | - | 31.2-500 μg/mL | 72 h | LC_50_ = 73.55, 120.85, and 131.54 μg/mL, respectively. | ([Liu et al., 2013](#_ENREF_87)) | |
|  | In vitro | Evodol and limonin | *Meloidogyne incognita* | - | 31.2-500 μg/mL | 72 h | LC_50_ = 155.02 and 197.37 μg/mL, respectively. | ([Liu et al., 2013](#_ENREF_87)) | |
|  | In vivo | Limonin | *Schistosoma mansoni*-infected mice | Oral | 50, 100 mg/kg | 21 and 56 days | (-): Worm burden; the hepatic and intestinal tissue egg loads. | ([Eraky et al., 2016](#_ENREF_15)) | |
|  | In vivo | EF essential oil | *Bacillus subtilis* and *Staphylococcus aureus* | - | 2 mg/mL | - | MIC = 3.2-6.4 mg/mL; MBC = 12.8 mg/mL. | ([Liu et al., 2019a](#_ENREF_89)) | |
|  | In vitro | EF methanol extract | *Xanthomonas oryzae* pv. *oryzae, Xanthomonas* *oryzae* pv. *oryzicola*, and *Xanthomonas campestris* pv. *campestris* | - | 50 µl | - | EC_50_ = 3.13, 14.32, and 32.72 nmol, respectively. | ([Su et al., 2018](#_ENREF_142)) | |
|  | In vitro | Evodiamine | LPS-primed J774A.1 and BMDM cells | - | 1.25, 2.5, 5 μmol/L | 1 h | (+): The NLRP3 inflammasome activation; innate immunity against bacterial infection | ([Li et al., 2019a](#_ENREF_64)) | |
|  | In vivo | Evodiamine | MSU-induced peritonitis in C57BL/6 mice | Oral | 20 mg/kg | 1 h | (-): The bacterial load and liver inflammation. (+): The survival of mice with lethal bacterial infection; production of IL-1β and interferon-γ. | ([Li et al., 2019a](#_ENREF_64)) | |
| Anti-osteoporosis | In vitro | Rutaecarpine | Bone marrow-derived macrophages | - | 0.1, 1, 5, 10 μmol/L | 72 h | (-): The protein level of NFATc1. | ([Fukuma et al., 2018](#_ENREF_19)) | |
|  | In vitro | Rutaecarpine | Isolated mice bone marrow macrophage | - | 0-30 μg/mL | 48 h | (-): The osteoclast formation; the expression of osteoclast marker genes and protein. | ([Jiang et al., 2017](#_ENREF_42)) | |
|  | In vitro | Evodiamine | Isolated C57BL/6 mice bone marrow macrophage‐derived osteoclast | - | 1, 2.5, 5, 10 μmol/L | 5 days | (-): The osteoclast formation; hydroxyapatite resorption; the expression of osteoclast marker gene and protein. | ([Jin et al., 2019](#_ENREF_46)) | |
|  | In vivo | Evodiamine | Ovariectomized mice | Oral | 10 mg/kg every other day | 8 weeks | (-): Osteoclastogenesis. | ([Jin et al., 2019](#_ENREF_46)) | |
|  | In vivo | Evodiamine | Dexamethasone-induced osteoporosis in zebrafish | Oral | 50, 100 mg/kg | 6 days | (+): The content of calcium, phosphorus, and hydroxyproline; the calcium sink of horse fish; the area of bone formation; the level of ALP and TRAP. | ([Yin et al., 2019](#_ENREF_192)) | |
|  | In vivo | Limonin | Orchidectomized male rats | Oral | 200 mg/kg | 60 days | (+): The bone density and strength. | ([Mandadi et al., 2009](#_ENREF_101)) | |
|  | In vitro | Limonin | Osteoblastic MC3T3-E1 cells | - | 5, 10, 20, 40 μmol/L | 24 h | (+): The ALP activity, mineralization, and the expression of genes encoding osteoblast differentiation marker. | ([Lee et al., 2016](#_ENREF_59)) | |
|  | In vivo | Limonin | Ovariectomized rats | Oral | 250 mg/kg | 12 weeks | (-): The body weight. (+): The bone mineral density and content regulation. | ([Lee et al., 2016](#_ENREF_59)) | |
| Antiallergic | In vivo | Evodiamine and rutaecarpine | Male ICR mice and male SD rats | Oral | 10, 50 mg/kg | 1 h | (-): The protein expressions of proinflammatory cytokines; passive cutaneous anaphylaxis reaction and scratching behaviors. | ([Shin et al., 2007](#_ENREF_141)) | |
|  | In vitro | Evodiamine and rutaecarpine | IgE-antigen complex-induced RBL-2H3 cells and compound 48/80-induced rat peritoneal mast cells | - | 2, 5, 25 μmol/L | 4 h | (-): The level of TNF-α and IL-4. | ([Shin et al., 2007](#_ENREF_141)) | |
|  | In vitro | Limonin | PBMCs from food-allergic patients | - | 20 mg/mL | 10 days | (-): The level of IL-4. | ([Yang et al., 2014](#_ENREF_185)) | |
| Antioxidant | In vivo | Limonin | Male SD rats | Oral | 50, 150 mg/kg | 6 weeks | (-): The level of MDA. (+): SOD, GSH-Px activities, and the ability of total antioxidant capacity. | ([Li et al., 2016](#_ENREF_69)) | |
|  | In vitro | Limonin | Undifferentiated human SH-SY5Y neuroblastoma cells | - | 5, 10, 50 mol/L | 48 h | (-): The rate of cell survival. | ([Poulose et al., 2005](#_ENREF_119)) | |
|  | In vitro | Limonin | DPPH radical | - | 0.001-1 mg/mL | 120 min | - | ([Breksa and Manners, 2006](#_ENREF_5)) | |
| Antidepressant | In vivo | Evodiamine | Chronic unpredictable mild stress-induced rats | Oral | 10, 20 mg/kg | 2 weeks | (-): The corticosterone hypersecretion. (+): The expression of brain-derived neurotrophic factor-tropomyosin receptor kinase B. | ([Jiang et al., 2015](#_ENREF_43)) | |
| Prostate protection | In vitro | EF 70% ethanol extract | Benign prostatic hyperplasia-1 cells | - | 6.25, 12.5, 25. 50, 100, 200 μg/mL | 48 h | (-): The activity of 5α-reductase; cell viability; the expression of phosphor-ERK1/2 proteins, caspase-8/3. | ([Park et al., 2018](#_ENREF_115)) | |
|  | In vitro | EF essential oil | Prostate cancer-3 cells | - | 0.5, 2, 10, 50 μg/mL | 48 h | (+): The level of IL-2, IL-10 and IL-10/IL-2 ratios. | ([Yeh and Lin, 2021](#_ENREF_191)) | |
|  | In vitro | EF essential oil | Isolated BALB/c mice peritoneal macrophages and splenocytes | - | 0.5, 2, 10, 50 μg/mL | 48 h | (+): The level of IL-10 and IL-10/IL-2 by splenocytes; The level of IL-1β, TNF-α, IL-6, IL-10 and IL-10/TNF-α secreted by macrophages. | ([Yeh and Lin, 2021](#_ENREF_191)) | |
|  | In vitro | Evodiamine | DU145 human prostate cancer cells | - | 1.25, 2.5, 5.0 μmol/L | 48 h | (-): The cell migration. (+): The mitochondrial apoptosis. | ([Lei et al., 2022](#_ENREF_62)) | |
